# Inferring mammalian tissue-specific regulatory conservation by predicting tissue-specific differences in open chromatin

**DOI:** 10.1186/s12864-022-08450-7

**Published:** 2022-04-11

**Authors:** Irene M. Kaplow, Daniel E. Schäffer, Morgan E. Wirthlin, Alyssa J. Lawler, Ashley R. Brown, Michael Kleyman, Andreas R. Pfenning

**Affiliations:** 1 Department of Computational Biology, Carnegie Mellon University, Pittsburgh, PA USA; 2 Neuroscience Institute, Carnegie Mellon University, Pittsburgh, PA USA; 3 Department of Biology, Carnegie Mellon University, Pittsburgh, PA USA

**Keywords:** Gene expression evolution, Open chromatin prediction, Machine learning, Enhancers

## Abstract

**Background:**

Evolutionary conservation is an invaluable tool for inferring functional significance in the genome, including regions that are crucial across many species and those that have undergone convergent evolution. Computational methods to test for sequence conservation are dominated by algorithms that examine the ability of one or more nucleotides to align across large evolutionary distances. While these nucleotide alignment-based approaches have proven powerful for protein-coding genes and some non-coding elements, they fail to capture conservation of many enhancers, distal regulatory elements that control spatial and temporal patterns of gene expression. The function of enhancers is governed by a complex, often tissue- and cell type-specific code that links combinations of transcription factor binding sites and other regulation-related sequence patterns to regulatory activity. Thus, function of orthologous enhancer regions can be conserved across large evolutionary distances, even when nucleotide turnover is high.

**Results:**

We present a new machine learning-based approach for evaluating enhancer conservation that leverages the combinatorial sequence code of enhancer activity rather than relying on the alignment of individual nucleotides. We first train a convolutional neural network model that can predict tissue-specific open chromatin, a proxy for enhancer activity, across mammals. Next, we apply that model to distinguish instances where the genome sequence would predict conserved function versus a loss of regulatory activity in that tissue. We present criteria for systematically evaluating model performance for this task and use them to demonstrate that our models accurately predict tissue-specific conservation and divergence in open chromatin between primate and rodent species, vastly out-performing leading nucleotide alignment-based approaches. We then apply our models to predict open chromatin at orthologs of brain and liver open chromatin regions across hundreds of mammals and find that brain enhancers associated with neuron activity have a stronger tendency than the general population to have predicted lineage-specific open chromatin.

**Conclusion:**

The framework presented here provides a mechanism to annotate tissue-specific regulatory function across hundreds of genomes and to study enhancer evolution using predicted regulatory differences rather than nucleotide-level conservation measurements.

**Supplementary Information:**

The online version contains supplementary material available at 10.1186/s12864-022-08450-7.

## Background

The study of conservation has had a tremendous impact in multiple areas of mammalian biology. When a new genome is sequenced, conservation is applied to provide high-quality annotations of candidate exons, introns, promoters, and other likely functional genomic regions [1]. Regions of the human genome conserved across other primates or mammals show a stronger enrichment for disease-associated loci than any other evaluated category of regions [2]. Conversely, regions of the human genome that display accelerated evolution have been implicated in human-specific adaptation [3]. In endangered species, molecular conservation has been applied predict which regions of low heterozygosity may be impacting fitness [4]. Conservation has also been applied to find regions of the genome associated with the evolution of complex phenotypes across mammals and vertebrates more broadly, including the loss of limb function [5], loss of eyesight [6], and longevity [7]. The powers of these studies are still growing, with many consortia, including the Vertebrate Genomes Project [8], the Genome 10 K Project [9], the Bat1K Project [10], and the Zoonomia Consortium [4], sequencing, assembling, and aligning [11] genomes from hundreds of mammals, including endangered species and species that live in remote parts of the world. Using these data, we can investigate conservation by comparing the DNA sequences of species whose most recent common ancestors lived tens of millions of years ago.

The methods used to infer conservation across species, including those applied to many of the challenges described above, typically rely on nucleotide-level constraint. They generally begin by aligning the nucleotides of multiple genomes together [12, 13]. From those alignments, PhyloP calculates nucleotide-level constraint relative a neutral model of evolution, which can be aggregated across broader regions to identify signatures of conservation or acceleration [3, 13, 14]. To study the diversity of mammalian phenotypes, nucleotide alignments are often modeled in the context of a tree structure to look for signatures of positive or negative selection [15–18]. Once nucleotide-level selection has been inferred, additional techniques have been applied to link those patterns of selection to convergent evolution, instances where a specific phenotype has evolved independently in multiple lineages [6, 18, 19].

As new genomic resources have become available and computational techniques have advanced, it has become clear that a large component of phenotypic evolution is mediated by differences in *cis*-regulatory elements, many of which are enhancers that control gene expression [20–22]. Within the human population, enhancers show a strong enrichment for disease-associated genetic variants [23, 24]. Across species, nucleotide-level selection in enhancers has been associated with the loss of eyesight, hindlimbs, and external testes [6, 19, 25, 26], and, in the last few years, with craniofacial development [27], response to ocean cooling [28], and vocal learning [29]. Numerous other complex phenotypes have been linked to gene expression differences across species, including domestication [30, 31], longevity [32–34], brain size [35], echolocation [36], and monogamy [37]. While nucleotide-level selection in enhancers is being applied to study the evolution of some of these phenotypes [38], recent studies of enhancers across species suggest a model of evolution in which nucleotide-level conservation of enhancers can be low in spite of enhancers maintaining their function [39, 40].

Much of our knowledge of enhancers comes from regulatory genomics measurements that are associated with enhancer activity, especially the Assay for Transposase-Accessible Chromatin using sequencing (ATAC-seq) and the DNase hypersensitivity assay for open chromatin and chromatin immunoprecipitation sequencing (ChIP-Seq) for the histone modifications H3K27ac and H3K4me1 [41–44]. Studies involving these assays have demonstrated that enhancers, relative to genes, are substantially more tissue- or cell type-specific and generally less conserved across species [45–48]. Within a given cell type or tissue, a combinatorial code of transcription factor (TF) binding motifs and other sequence patterns determines the ultimate regulatory activity of enhancers [49–51]. In a striking example of this, a recent study found that an Islet enhancer’s developmental function remains remarkably conserved across the 700 million years of evolution between mammals and sponges by maintaining a similar set of TF motifs despite negligible detectable conservation at the nucleotide level [39]. This understanding is further supported by studies of TF binding across species that display a large turnover in individual binding sites [52–54], even though gene expression is often highly conserved, and genes with highly conserved expression often have conservation in the TFs whose motifs occur within their candidate enhancers [55]. Therefore, to study conservation of many enhancers, new methods are required that link genome sequence differences at candidate enhancers to differences in enhancer function.

Advances in the application of machine learning techniques to regulatory genomics enable us to evaluate conservation at the level of the regulatory code rather than the alignment of individual nucleotides. In one recent study, support vector machines (SVMs) and convolutional neural networks (CNNs) were able to predict which 3 kb regions have the enhancer-associated histone modification H3K27ac in brain, liver, and limb tissue of human, macaque, and mouse [56]. Importantly, the study found that models trained in one mammal achieved high accuracy in another mammal in the same clade and in another mammal in a different clade, suggesting that the regulatory code in all three of these tissues is highly conserved across mammals [57]. Another study obtained a similar result for regions associated with H3K27ac [58], and two other studies have obtained similar results using another proxy for enhancer activity – open chromatin regions (OCRs) [59, 60]. One of these studies found that training CNNs on OCRs from multiple mammals had better performance than training CNNs on OCRs from a single mammal, albeit using 131,072 bp sequences as input. The boost in power from incorporating multiple species generalized to predicting TF binding strength from ChIP-seq data and gene expression from RNA-seq data [59]. An additional study found that a combined CNN-recurrent neural network [61, 62] trained on sequences underlying 500 bp OCRs from melanoma cell lines in one species can accurately predict melanoma cell line open chromatin in other species at a wide range of genetic distances from the training species, including in parts of the genome with low sequence conservation between the training and evaluation species [60].

While these studies represent major advances in cross-species enhancer prediction, they have yet to comprehensively demonstrate an ability to identify sequence differences between species that are associated with differences in regulatory genomic measurements of enhancer activity, which is crucial for their application as a conservation metric (Table 1). In fact, an additional study trained SVMs to predict liver enhancers using dinucleotide-shuffled candidate enhancers as negatives. While the overall performance was good, human enhancers whose orthologs are active in Old World monkeys but not New World monkeys were predicted to have consistent activity across all primates, showing that models with good overall performance do not always work well on enhancer orthologs whose activities differ between species [63].

**Table 1:** Comparison of evaluation criteria used to evaluate models in candidate enhancer activity conservation prediction papers

Evaluation Criterium	Chen et al. 2018 [57]	Huh et al. 2018 [58]	Kelley 2020 [59]	Minnoye et al. 2020 [60]	This Paper
Evaluation on Genomic Regions not Used in Training	✓	✓	✓	✓	✓
Evaluation on Species not Used in Training	✓	✓	✓	✘	✓
Comparison of Model Trained on One Species to Model Trained on Multiple Species	✘	✓	✓	✘	✓
Evaluation of Lineage-Specific Enhancer Accuracy	✘	✘	✘	✘	✓
Evaluation of Tissue-Specific Enhancer Accuracy	✘	✘	✘	✘	✓
Evaluation of Phylogeny-Matching Correlations	✘	✘	✘	✘	✓
Comparison to Conservation Scores	✘	✘	✘	✘	✓
Identification of Biologically Relevant Enhancers with Correctly Predicted Lineage-Specificity	✘	✘	✘	✓	✓

**Fig. 1:**
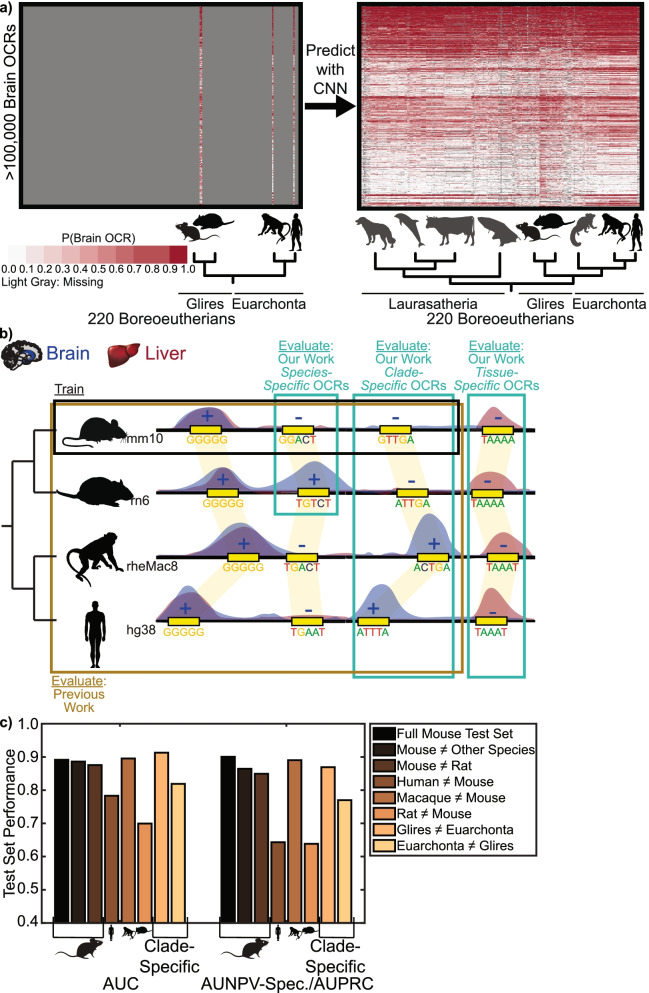
OCR Ortholog Open Chromatin Status Prediction Framework Overview. **a** We trained a convolutional neural network (CNN) for predicting brain open chromatin using sequences underlying brain open chromatin region (OCR) orthologs in a small number of species and used the CNN to predict brain OCR ortholog open chromatin status across the species in the Zoonomia Consortium. Specifically, we used the sequences underlying the orthologs for which we have brain open chromatin data to train a CNN for predicting open chromatin. Then, we used the CNN to predict the probability of brain open chromatin for all brain OCR orthologs; predictions are illustrated on the right. Animals for which we do not have open chromatin data are in dark gray instead of black to indicate that their brain open chromatin is imputed. While we cannot evaluate the accuracy of most of our predictions, obtaining open chromatin data from most tissues in most species is infeasible, so predictions might be the best OCR annotations that we can obtain. **b** To demonstrate that our models can accurately predict whether sequence differences between species are associated with open chromatin differences, in addition to the evaluations described in previous work [57], we evaluated our performance on species-specific open chromatin for a species not used in model training and clade-specific open and closed chromatin for clades not used in model training. Since such regions often comprise a minority of OCR orthologs, models could obtain good overall performance while obtaining poor performance on such regions. We also evaluated our performance on tissue-specific open and closed chromatin for a tissue not used in model training, where we expect models to predict 0 if model learns sequence signatures related to the tissue used in training. **c** Full mouse test set and lineage-specific OCR accuracy evaluations for mouse sequence-only brain model, illustrating that, even for the best of these models, performance on clade-specific and species-specific OCRs and non-OCRs for clades and species not used in training is not as good as performance on the full test set. Animal silhouettes were obtained from PhyloPic [65].

To investigate the feasibility of accurately predicting candidate *cis*-regulatory element differences across species, we chose to focus on OCRs, which are only a proxy of regulatory activity, but whose high resolution has the potential to identify specific genome sequence differences associated with putative regulatory activity. We leveraged new, controlled open chromatin experiments conducted by our laboratory [64] and trained a set of new models to predict OCR differences across species (Fig. 1). We evaluated model performance using new criteria that we developed for this task, which focus on the ability to predict similarities and differences across species and tissues rather than the large number of regions that are consistently open or closed (Fig. 1b). We also developed a novel method for associating previously identified candidate enhancer sets with predicted lineage-specific open chromatin and lack of open chromatin, in which we clustered OCRs based on their predicted open chromatin and identified clusters overlapping candidate enhancer sets more than would be expected by chance. While we developed our methods and evaluation criteria for open chromatin, they can be applied to any high-resolution measurement of enhancer activity. Thus, our methods for evaluating approaches to enhancer ortholog enhancer activity prediction and for identifying predicted lineage-specific enhancers associated with candidate enhancer sets can be applied to any tissue or cell type with enhancer activity data from multiple species. This allows for putative annotation of enhancer activity across the hundreds of new genomes that are being sequenced, providing a valuable resource for research communities studying gene regulation in non-model organisms, especially where direct measurements are not feasible. We anticipate that this work will encourage researchers to develop and properly evaluate new models for predicting enhancer ortholog enhancer activity across species and help reveal potential functional roles of lineage-specific enhancers, enabling us to uncover transcriptional regulatory mechanisms underlying the evolution of mammalian phenotypic diversity.

## Results

### Dataset construction for evaluating approaches for OCR ortholog open chromatin prediction

To demonstrate the ability to predict OCR differences across species, we curated publicly available data to create a dataset of brain OCRs and their orthologs across dozens of species (Methods). Specifically, we created two positive sets: The first consisted of brain OCRs from *Mus musculus* [66], which we used to evaluate the ability of a model trained in one species to generalize to species not used in training. The second consisted of brain OCRs from *Homo sapiens* [41, 67, 68], *Macaca Mulatta* [64], *Mus musculus* [66], and *Rattus norvegicus* [64]. Rather than focus on longer regions, which have been shown to work well in some previous studies [57, 59], we used 500 bp regions for multiple reasons. 500 bp is the approximate resolution of open chromatin, shorter regions help the model to focus on the impact of local sequence differences, obtaining orthologs of shorter regions in genomes with short scaffolds is more often feasible than obtaining orthologs of longer regions, and predictions of enhancer activity for shorter regions are easier to experimentally validate than predictions for longer regions are. We compared performance for two measures of conservation scores – PhastCons [13] and PhyloP [14] – and machine learning models trained using our mouse-only positive set and each of five different mouse negative sets that are similar to those used for in previous work for related tasks. These negative sets were: (1) flanking regions [69], (2) OCRs from other tissues [59, 60], (3) about ten times as many G/C- and repeat-matched regions as positives [57], (4) about twice as many G/C- and repeat-matched regions as positives, and (5) ten dinucleotide-shuffled versions of each positive [70] (Supplemental Fig. 1a). We additionally created a sixth, novel negative set to help the model learn signatures of OCRs whose open chromatin is not conserved–brain closed chromatin regions in a species whose orthologs in another species are brain OCRs (called “non-OCR orthologs of OCRs,” white regions in left part of Fig. 1). We combined this new negative set with the positive set as a part of our comparison (Supplemental Fig. 1a). We refer to the combination of the mouse-only positive set and each mouse negative set as a “training set.” For a modeling approach, we chose CNNs [56, 71] for multiple reasons. CNNs can model combinatorial relationships between sequence patterns, and changes in a single TF motif often do not cause changes in open chromatin [51]; CNNs do not require an explicit featurization of the data, and many sequence patterns involved in brain open chromatin are unknown; and CNNs make predictions quickly relative to SVMs, the other leading approach for related tasks [57]. While we had only tens of thousands of examples for most of our training sets (Table 2), multiple previous studies have trained CNNs to predict measures of enhancer activity with training sets of similar sizes [57, 60, 72], so we were optimistic that our datasets would be sufficiently large for CNN training. We did the initial comparisons using conservation scores generated using mouse reference-based alignments [12, 73] and models trained on only mouse sequences so that we could evaluate their performance on both closely and distantly related species not used for training the machine learning models [57].

**Table 2:** Number of positives and negatives used for training, tuning, and testing each model

Model Number	Genomes Used in Training	Tissue	Negative Set	Positives (Training, Validation, Test)	Negatives (Training, Validation, Test)	Negatives:Positives (Training, Validation, Test)
1	mm10	Brain	Flanking Regions	21,594, 2416, 4576	35,640, 4018, 7440	1.65:1, 1.66:1, 1.63:1
2	mm10	Brain	OCRs in Other Tissues	21,594, 2416, 4576	427,174, 70,504, 82,172	19.78:1, 29.18:1, 17.96:1
3	mm10	Brain	Large G/C- and Repeat-Matched	21,594, 2416, 4576	175,912, 23,880, 32,008	8.15:1, 9.88:1, 6.99:1
4	mm10	Brain	Small G/C- and Repeat-Matched	21,594, 2416, 4576	35,358, 4776, 6654	1.64:1, 1.98:1, 1.45:1
5	mm10	Brain	Dinucleotide-Shuffled OCRs	21,594, 2416, 4576	215,940, 24,160, 45,760	10:1, 10:1, 10:1
6	mm10	Brain	Non-OCR Orths. of OCRs	21,594, 2416, 4576	25,086, 3456, 4694	1.16:1, 1.43:1, 1.03:1
7	mm10	Liver	Non-OCR Orths. of OCRs	32,498, 4032, 7752	22,890, 2994, 4434	1:1.42, 1:1.35, 1:1.75
8	mm10, hg38, rheMac8, rn6	Brain	Non-OCR Orths. of OCRs	74,688, 9036, 15,266	111,206, 14,650, 19,688	1.49:1, 1.62:1, 1.29:1
9	mm10, rheMac8, rn6	Liver	Non-OCR Orths. of OCRs	81,886, 10,428, 17,688	67,278, 8680, 14,544	1:1.22, 1:1.20, 1:1.22

### Best overall performances does not guarantee lineage-specific OCR accuracy

Although all our brain models trained on mouse sequences had good overall performance (Supplemental Notes, Supplemental Fig. 1b), the many OCRs whose orthologs are open in many species are much less likely to be involved in gene expression differences between species than the few whose open chromatin statuses differ between species, so we needed to also evaluate our models on the strict subset of likely relevant sequences. We therefore created a group of “evaluation sets” for evaluating the ability of our models to make accurate predictions on these sequences and evaluated all of our mouse sequence-only brain models on the same evaluation sets. We began by evaluating whether our models and conservation scores can obtain “lineage-specific OCR accuracy,” the ability to predict open chromatin differences between species (Fig. 1b). We did this by evaluating models for the OCR orthologs whose brain open chromatin status differ between species, an evaluation not done in previous studies, which evaluated only overall performance in species not used in training (Table 1) [57]. Specifically, we evaluated performance on a few subsets of brain OCRs and non-OCRs: mouse regions whose open chromatin status differ in another species (MouseBr ≠ OtherBr), regions in species not used in training whose open chromatin status differ from mouse (HumanBr ≠ MouseBr, MacaqueBr ≠ MouseBr, RatBr ≠ MouseBr), regions whose open chromatin status differ between closely related species used and not used in training (MouseBr ≠ RatBr, RatBr ≠ MouseBr), and regions whose open chromatin status differ between clades used and not used in training (GliresBr ≠ EuarchontaBr, EuarchontaBr ≠ GliresBr). We found that all our machine learning models achieved decent performance for all these criteria. However, the model trained on training set with the dinucleotide-shuffled brain OCR negatives, which had the best test set performance, had the worst or second-worst performance for all these evaluation sets (Fig. 1c, Supplemental Figs. 1b-i)), demonstrating the necessity of these additional evaluations. Although some models seemed poorly calibrated (0.5 might be a non-ideal positive class threshold) for GliresBr ≠ EuarchontaBr and EuarchontaBr ≠ GliresBr, re-calibrating models with the positives and our novel negative set did not substantially change their relative performances (Supplemental Notes, Supplemental Tables 1–6, Supplemental Fig. 2).

**Fig. 2:**
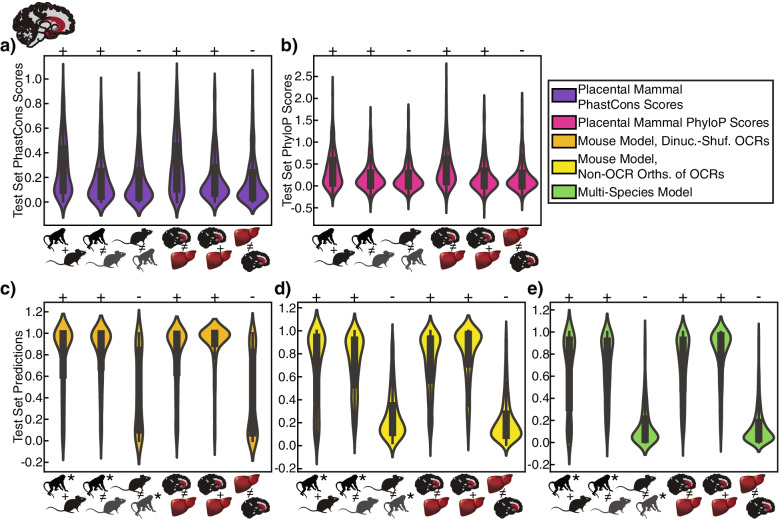
Violin Plots for Brain Model Lineage-Specific and Tissue-Specific OCR Accuracy Evaluation in Macaque. Comparison of **a** PhastCons [13] and **b** PhyloP [14] scores to **c-e** three different machine learning models’ predictions for brain OCRs with conserved open chromatin across mouse and macaque, macaque brain OCRs whose mouse orthologs are closed in brain, macaque brain non-OCRs whose mouse orthologs are open in brain, macaque brain OCRs that are closed in liver, macaque brain OCRs that are open in liver (centered on brain peak summits), and macaque liver OCRs that are closed in brain. + ’s indicate that values should be large, and -‘s indicate that values should be small. Conservation scores were generated from the mm10-based placental mammals alignment [12, 73] and averaged over 500 bp centered on peak summits, where mouse peak summits were used for OCRs conserved between mouse and macaque and for OCRs in mouse whose macaque orthologs are closed, and mouse orthologs of macaque peak summits were used for other evaluations. All machine learning model predictions were made using macaque sequences. The macaque sequences for OCRs conserved between mouse and macaque and for OCRs in mouse whose macaque orthologs are closed were centered on macaque orthologs of mouse peak summits, and macaque peak summits were used for other evaluations. Note that the models in **c** and **d** were trained on only mouse sequences, demonstrating their performance in a species not used in training. Animal silhouettes were obtained from PhyloPic [65]. *’s indicate the species from which sequences were obtained for making predictions. Dinuc.-shuf. stands for dinucleotide-shuffled, and orths. stands for orthologs

We also compared the predictions of our machine learning model trained with our novel negative set and of our model trained with the dinucleotide-shuffled negatives to conservation scores for macaque regions with different brain activity conservation patterns. The region sets were those open in the mouse and macaque orthologs (expect positive prediction), those open in the macaque ortholog but closed in the mouse ortholog (expect positive prediction), and those closed in the macaque ortholog but open in the mouse ortholog (expect negative prediction). We found that conservation scores tended to be only slightly lower for regions whose open chromatin differed between species than for regions open in both species. We also found that conservation scores could not clearly distinguish between whether an OCR’s ortholog was open in mouse or in macaque (Fig. 2a-b). On the other hand, the machine learning models generally had larger predictions for macaque open regions than they did for macaque closed regions, regardless of whether the mouse ortholog was open, though the model trained on the dinucleotide-shuffled brain OCR negatives predicted that almost half of the macaque non-OCRs whose mouse orthologs are OCRs are open in brain (Fig. 2c-e). We repeated this same analysis for human and rat regions with the same and differing open chromatin statuses from mouse and obtained similar results (Supplemental Fig. 3, Supplemental Fig. 4a). We did additional evaluations to further show that conservation scores are significantly inferior to our best machine learning models at predicting open chromatin conservation across lineages (Supplemental Notes, Supplemental Tables 7–9). These results demonstrate the limited ability of conservation scores to reveal if open chromatin is conserved and to identify the species in which OCRs with open chromatin conserved in a strict subset of species are open.

### Best overall model performance does not guarantee tissue-specific OCR accuracy

Many known OCRs are in closed chromatin regions in the brain [48, 74, 75], so we also need to ensure that our models predict that OCRs that are open in only non-brain tissues are closed in the brain. We therefore also determined if conservation scores and our machine learning models achieved high “tissue-specific OCR accuracy” (Fig. 1b) – accurate performance on tissue-specific OCRs and non-OCRs, an evaluation that has not been done directly in any previous studies (Table 1). To determine if our models learned sequence patterns associated with only brain-specific open chromatin or only shared open chromatin across tissues, we evaluated our models’ predictions for the subset of brain OCRs that do not overlap liver OCRs and the subset of brain OCRs that do overlap liver OCRs. Test chromosome predictions from all models for both subsets of the positive set were usually close to one (Supplemental Fig. 5a). We also evaluated our models’ predictions for the liver OCRs that do not overlap brain OCRs, for which we would expect negative predictions, and compared this to the predictions on the negatives in the training set. We found that predictions on both sets tended to be close to zero. However, the liver, non-brain open chromatin status predictions from the model trained with dinucleotide-shuffled OCR negatives tended to be more evenly distributed between zero and one than the liver, non-brain open chromatin status predictions for the models trained with the other negative sets (Supplemental Fig. 5a). We also did a comparison of the performances of models with different negatives in their training sets in which we limited the positive set to brain OCRs that overlap liver OCRs and defined the negatives as liver OCRs that do not overlap brain OCRs (MouseBrVsLv, MacaqueBrVsLv, and RatBrVsLv). We found that all models worked well (AUPRC > 0.6) on mouse as well as on macaque and rat, which were not used in training, with the model trained on dinucleotide-shuffled brain OCR negatives performing the worst (Supplemental Fig. 5b), demonstrating the value of these new evaluation criteria. The difference in performance may be a result of differences between the TF motifs learned by the models (Supplemental Notes, Supplemental Fig. 6). Calibration with the mouse-only training set with our novel negative set had a similar effect to calibration for clade-specific open and closed chromatin regions (Supplemental Tables 10–12, Supplemental Fig. 5c).

We also compared the predictions from the model trained with our novel negative set, which was one of the best-performing models for these evaluations, and the model trained with dinucleotide-shuffled OCR negatives to conservation scores. Unlike the machine learning model predictions, the conservation scores for brain, non-liver OCRs; OCRs in brain and liver; and liver, non-brain OCRs are similar (Fig. 2a, Supplemental Figs. 2–3), demonstrating that conservation scores, in contrast to most of our machine learning models, provide little information about the tissue-specificity of open chromatin.

### Predictions from models of OCR ortholog open chromatin status have phylogeny-matching correlations

We also determined whether our models’ predictions have “phylogeny-matching correlations.” We did this by making open chromatin predictions at mouse OCR orthologs in dozens of species and determining if our predictions tend to match what we would expect based on the amount of time since the species diverged from mouse. All our models had strong performance (Supplemental Notes, Supplemental Fig. 7).

### Machine Learning Models Trained Using Data from Multiple Species Can Accurately Predict OCR Orthologs’ Open Chromatin Statuses

**Fig. 3:**
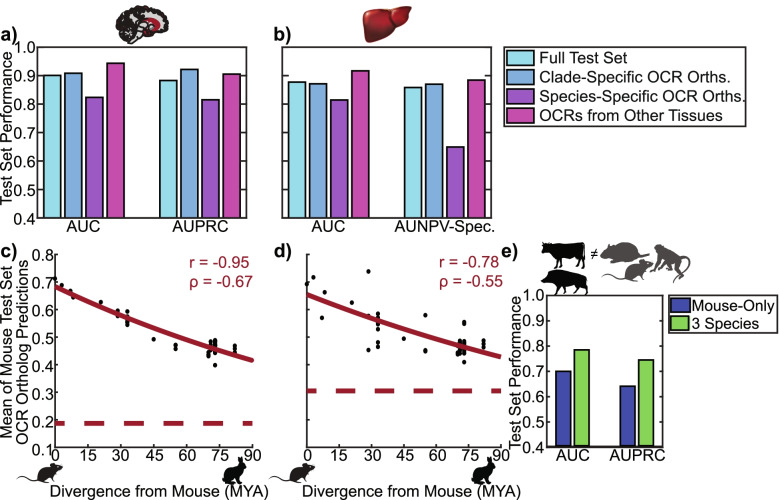
Multi-Species Model Performance. **a** Performance of multi-species brain model on MultiBr, MultiBrClade, MultiBrSpecies, and MultiBrVsLv (Supplemental Tables 20–21). **b** Performance of multi-species liver model on MultiLv, MultiLvClade, MultiLvSpecies, and MultiLvVsBr (Supplemental Tables 20–21). We reported area under the negative predictive value (NPV)-specificity (Spec.) curve instead of the AUPRC because these evaluation sets have more positives than negatives. **c** Divergence from mouse versus mean multi-species brain model predictions across mouse test chromosome brain OCR orthologs in Glires. **d** Divergence from mouse versus mean multi-species liver model predictions across mouse test chromosome liver OCR orthologs in Glires. **e** Performance of mouse-only liver model versus multi-species liver model on MultiLvLauras (Supplemental Tables 20–21). Animal silhouettes were obtained from PhyloPic [65]. AUC stands for area under the receiver operating characteristic curve, AUPRC stands for area under the precision-recall curve, and MYA stands for millions of years ago. The red curves are the best fit exponential function of the form y = ae^bx^. The red dotted lines are the average prediction across test set negatives.

Based on other research showing that training models with data from multiple species can improve OCR prediction accuracy [59], we trained additional machine learning models using open chromatin data from multiple species. For each of brain and liver, we used the open chromatin data from all the species that we had collected as positives (four species for brain, three species for liver) and the orthologs of all these OCRs in the other species for which we had data that did not overlap brain or liver open chromatin, respectively, as negatives. Before training multi-species models, we trained mouse-only models for liver and found that they achieved good performance for all our criteria (Supplemental Notes, Supplemental Figs. 8–9). We then trained brain and liver multi-species models (Tables 2–3) and found that they achieved high lineage-specific and tissue-specific OCR accuracy, where performance was generally better than the performance achieved by any of the models trained on only mouse sequences (Figs. 2, 3a-b, Supplemental Fig. 9). We also evaluated the multi-species liver model’s ability to predict conservation of H3K27ac ChIP-seq and found that it worked well (Supplemental Notes, Supplemental Table 13). In addition, we determined whether the multi-species brain and liver models’ predictions had phylogeny-matching correlations by using them to predict the OCR ortholog open chromatin status of mouse brain and liver OCRs, respectively, across Glires and found strong negative correlations between divergence from mouse and mean OCR ortholog open chromatin status predictions (Fig. 3c-d). We also found strong positive correlations between divergence from mouse and standard deviations of OCR ortholog open chromatin status predictions (Supplemental Figs. 10a-b). For both these models and our mouse-only models, our phylogeny-matching correlation results could not be fully explained by genome quality (Supplemental Notes, Supplemental Fig. 11, Supplemental Tables 14–15). Finally, we evaluated our mouse-only and multi-species liver models on Laurasiatheria-specific liver OCRs and liver non-OCRs [76], as no Laurasiatheria were used in training either model, and found that the multi-species liver model had better performance than the mouse-only liver model (MultiLvLauras, Fig. 3e). Like our mouse-only models, our multi-species brain and liver models also learned TF motifs for TFs that are known to be involved in brain and liver, respectively (Supplemental Notes, Supplemental Figs. 10c-d). In addition, their predictions were significantly more associated with open chromatin conservation than were conservation scores (Fig. 2, Supplemental Notes, Supplemental Figs. 2–3, Supplemental Fig. 9, Supplemental Table 16).

**Table 3:** Hyper-parameters for multi-species models (models 8–9)

Hyper-Parameter	Hyper-Parameter Value
Number of Filters per Convolutional Layer	350
Width of Convolutional Filters	7
Stride of Convolutional Filters	1
Number of Convolutional Layers	5
Dropout for Each Convolutional Layer	0.2
L2 Regularization for Each Convolutional Layer	0.00001
Max-Pooling Width	26
Max-Pooling Stride	26
Number of Units in First Fully Connected Layer	300
Optimizer	Stochastic Gradient Descent
Learning Rate	0.001
Momentum	0.99 (Nesterov)
Batch Size	100
Class-Weighting	Fraction of Examples in the Other Class

**Fig. 4:**
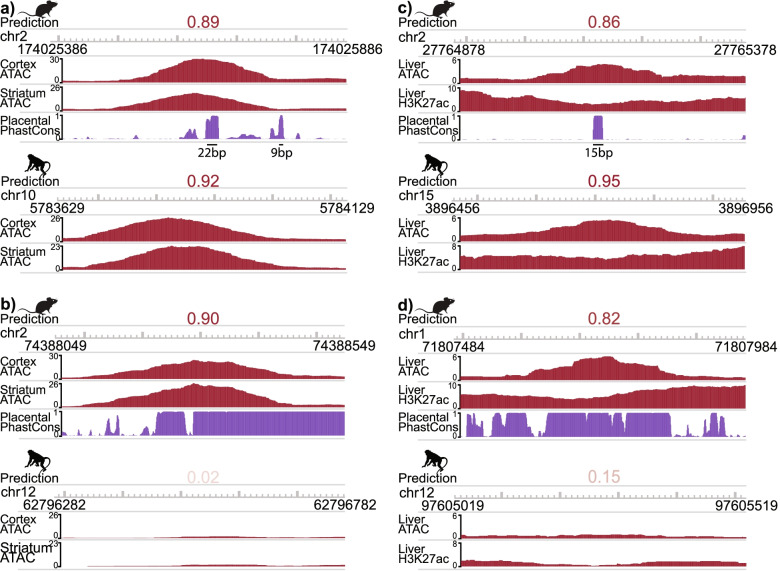
Examples of Mean Conservation Score and Open Chromatin Status Prediction versus Open Chromatin Conservation. **a** 7-week-old mouse cortex and striatum and macaque orofacial motor cortex (“Cortex”) and putamen (“Striatum”) open chromatin signal for a mouse brain OCR that is 50,328 bp away from the *Stx16* transcription start site (TSS). Experimentally identified and predicted brain open chromatin statuses are conserved even though mean mouse PhastCons score is low. **b** 7-week-old mouse cortex and striatum and macaque orofacial motor cortex (“Cortex”) and putamen (“Striatum”) open chromatin signal for a mouse brain OCR that is 144,474 bp away from the *Lnpk* TSS. Experimentally identified and predicted brain open chromatin statuses are not conserved even though mean mouse PhastCons score is high. **c** Our mouse liver open chromatin, mouse liver H3K27ac ChIP-seq, and macaque liver open chromatin signal for a mouse liver OCR that is 24,814 bp away from the *Rxra* TSS. Experimentally identified and predicted liver open chromatin statuses are conserved even though mean mouse PhastCons score is low. **d** Our mouse liver open chromatin, mouse liver H3K27ac ChIP-seq, and macaque liver open chromatin signal for a mouse liver OCR that is 154,404 bp away from the *Fn1* TSS. Experimentally identified and predicted liver open chromatin statuses are not conserved even though mean mouse PhastCons score is high. Animal silhouettes were obtained from PhyloPic [65]. Regions are mouse cortex or liver open chromatin peak summits ± 250 bp and their macaque orthologs, signals are from pooled reads across biological replicates, and liver H3K27ac ChIP-seq data was obtained from E-MTAB-2633 [40].

Some of the OCR orthologs for which our multi-species brain model outperformed conservation scores (i.e., correctly predicted brain open chromatin conservation despite low mean sequence conservation or correctly predicted lack of brain open chromatin conservation despite high mean sequence conservation) are near genes that have been shown to play important roles in the brain. For example, there is a region on mouse chromosome 2 – part of our test set – that has low mean sequence conservation according to PhastCons [13] and PhyloP [14] but high brain experimentally identified and predicted open chromatin conservation between mouse and macaque (Fig. 4a). This OCR’s mouse and macaque orthologs are located near the gene *Stx16*. STX16 is involved in vesicle trafficking in most tissues, including the brain [77], and may play a role in Alzheimer’s disease [78]; in fact, its role in axon regeneration is conserved between mammals and the roundworm *C. elegans* [79]. Although this open chromatin region near *Stx16* has generally low sequence conservation, running TomTom [80] on the 22 bp sequence with high conservation revealed a subsequence that is similar to the FOS motif, which is also found in the macaque ortholog. Since our machine learning model used sequence similarity to the FOS motif in making predictions (Supplemental Fig. 10c), the machine learning model was likely able to automatically determine that it should use this 22 bp sequence in making its prediction. In addition, there is a region on mouse chromosome 2 that has high mean sequence conservation but low experimentally identified and predicted open chromatin conservation between mouse and macaque (Fig. 4b) and whose mouse and macaque orthologs are located near the gene *Lnpk*. This gene is an endoplasmic reticulum junction stabilizer [81] that has been shown to play a role in brain and limb development [82], and mutations in this gene have been associated with neurodevelopmental disorders [83]. It is possible that this region of the genome near *Lnpk* has a high degree of sequence conservation because it has functions in other tissues. Although this mouse OCR near *Lnpk* has generally high sequence conservation, running FIMO [84] on the mouse ortholog’s summit ± 250 bp using the motifs learned by our multi-species brain model (Supplemental Fig. 10c) revealed a motif similar to that of EGR2 (q-value = 0.00044). In contrast, repeating this process on the macaque ortholog summit ± 250 bp did not reveal any occurrences of these motifs (q-value of best match to any important motif for positive predictions = 0.19, q-value of best match to motif similar to EGR2 = 0.62); this may explain why the model was able to accurately predict this difference in open chromatin. These results demonstrate the benefits of using OCR ortholog open chromatin status predictions instead of mean conservation scores for studying the evolution of the expression of important genes in a tissue of interest.

In addition, some of the accurate multi-species model liver open chromatin conservation predictions disagreed with the mean conservation scores. For instance, there is a region on mouse chromosome 2 that has high experimentally identified and predicted liver open chromatin conservation but low sequence conservation (Fig. 4c) and whose mouse and macaque orthologs are located near *Rxra*. This gene is a TF involved in regulating lipid metabolism [85–87], TF-MoDISco identified a sequence similar to its motif as being important in our liver models (Supplemental Fig. 8f, Supplemental Fig. 10d), and its liver expression is stable across fifteen mammals [88]. Although this region near *Rxra* has generally low sequence conservation, the 15 bp segment with high conservation is similar to the motif for CTCF according to TomTom [80], and that motif is also found in the macaque ortholog. Since our machine learning model used sequence similarity to the CTCF motif in making predictions (Supplemental Fig. 10d), the machine learning model was likely able to automatically determine that it should use this sequence in making its prediction. There is also a region on mouse chromosome 1, which is also part of our test set, whose mouse ortholog is an OCR and macaque ortholog is not an OCR according to our data and predictions despite being highly conserved (Fig. 4d) and whose mouse and macaque orthologs are near *Fn1*. This gene has been implicated in liver fibrosis [89–91], and a multi-species liver RNA-seq study found that it has higher expression in mouse liver relative to livers of other mammals and birds and lower expression in primate livers relative to livers of other mammals and birds [92]. For both of these OCRs, the H3K27ac signal conservation in the same regions [40] is similar to the open chromatin status conservation, suggesting that the open chromatin status conservation is indicative of enhancer activity conservation (Fig. 4c-d). These results suggest that using predicted OCR ortholog open chromatin status instead of conservation is beneficial for understanding gene expression evolution in multiple tissues.

### Lineage-specific brain and liver OCRs are associated with neuron and liver functions

Since our models can accurately predict lineage-specific OCRs, we evaluated whether lineage-specific brain and liver OCRs were associated with brain and liver functions. We note that these analyses are not feasible with conservation scores because they require measurements or predicted measurements of enhancer activity in dozens of species, and conservation scores are currently available for only human and house mouse. We did these analyses by clustering each of the brain and liver OCRs, where the features were the predicted activity in each of the Boreoeutheria from the Zoonomia Project [4]. We first used k-means clustering to cluster the OCRs into thousands of small clusters and then used affinity propagation clustering to cluster the smaller clusters into larger clusters. We selected affinity propagation clustering because we did not know how many clusters to expect, and affinity propagation clustering automatically determines the number of clusters. For each of brain and liver, we obtained slightly over one hundred clusters with different patterns of predicted open chromatin across species (brain clusters: https://github.com/pfenninglab/OCROrthologPrediction/clusters/brain, liver clusters: https://github.com/pfenninglab/OCROrthologPrediction/liver).

**Fig. 5:**
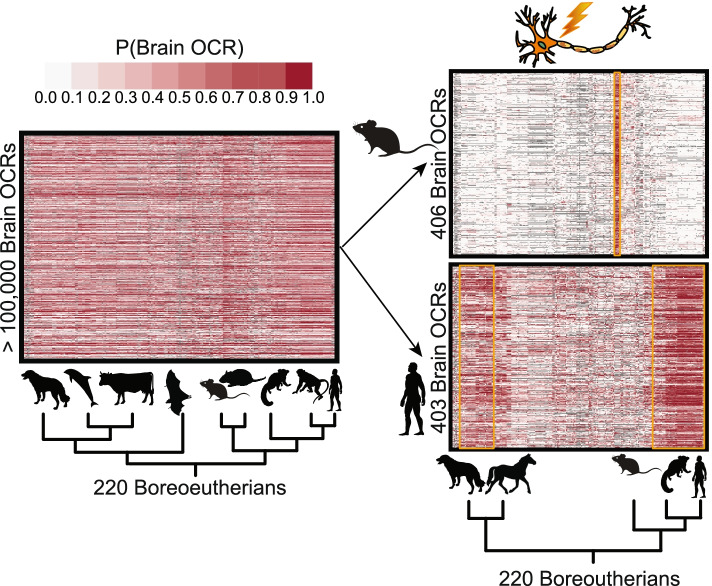
Examples of Brain OCR Clusters with Predicted Lineage-Specific Open Chromatin Associated with Neuron Activity. We clustered the brain OCRs, where the features were the brain predictions in each Boreoeutheria species from Zoonomia, and then identified clusters whose regions had significant overlap with regions associated with mouse neuron firing and human neuron activity. Mouse neuron firing enhancers had significant overlap with a predicted Murinae-specific OCR cluster (cluster 43), and human neuron activity enhancers had significant overlap with a predicted Euarchonta and Carnivora-specific non-OCR cluster (cluster 74). Animal silhouettes were obtained from PhyloPic [65]

We then determined whether each brain cluster that was open in mouse overlapped mouse candidate enhancers associated with neuron firing [93] more than expected by chance (Supplemental Table 17) and each brain cluster that was open in human overlapped human candidate enhancers associated with neuron activity [94] more than expected by chance (Supplemental Table 18). Interestingly, the candidate enhancers from each of these sets intersected clusters with predicted lineage-specific open chromatin or predicted lineage-specific lack of open chromatin more than expected by chance. Specifically, mouse neuron bicuculline (Bic)-specific candidate enhancers, where Bic induces neuron firing, were enriched for overlapping two predicted Murinae-specific brain open chromatin clusters – cluster 43 (Fig. 5) and cluster 27 (Supplemental Fig. 12a). We think that these results are unlikely to be explained by the number of usable orthologs or conservation because mouse brain OCRs overlapping Bic-specific candidate enhancers do not have significantly fewer usable orthologs or lower conservation according to PhastCons [13] or PhyloP [14] than mouse brain OCRs in general. In contrast, mouse activity-invariant candidate enhancers were enriched for overlapping the predicted open chromatin in all species cluster (cluster 1), a noisy cluster without a clear pattern of predicted open chromatin (cluster 37), a noisy predicted Yangochioptera-specific brain non-open chromatin cluster (cluster 81), and a noisy predicted Primate-specific brain non-open chromatin cluster (cluster 88). Likewise, human candidate enhancers from GABAergic neurons made from hiPSCs from four genotypes that had increased activity two hours after exposure to potassium chloride (KCl), where KCl induces neuron activity, were enriched for overlapping multiple clusters with clade-specific open or closed chromatin. These clusters included a predicted Carnivora-, Perissodactyla-, and Euarchonta-specific brain open chromatin cluster (cluster 74, Fig. 5); a predicted Hystricognathi-specific brain non-open chromatin cluster (cluster 11, Supplemental Fig. 12b); and a predicted Muroidea- and Pecora-specific brain non-open chromatin cluster (cluster 48, Supplemental Fig. 12b). These results are unlikely to be explained the number of usable orthologs or conservation because human brain OCRs overlapping this set of GABAergic neuron candidate enhancers tended to have more usable orthologs and higher conservation than human brain OCRs overall according to both PhastCons [13] and PhyloP [13]. In contrast, candidate enhancers from the same source that had decreased activity two hours after exposure to KCl were enriched for overlapping the predicted open chromatin in all species cluster (cluster 1) and a predicted Ruminantia-specific brain non-open chromatin cluster (cluster 82). These results suggest that there may be a relationship between enhancer response to neuron activity and whether the enhancer’s activity tends to be specific to the lineage in which it was identified. We also applied this approach to investigate the overlaps between liver clusters and mouse candidate enhancers associated with liver regeneration [95] and found a potential relationship between mouse liver regeneration and Murinae-specific open chromatin (Supplemental Notes, Supplemental Table 19, Supplemental Fig. 12c).

## Discussion

### Developing a tissue-specific metric of regulatory conservation

While there are well-established methods for quantifying nucleotide-level conservation based on a sequence alignment [13, 14, 96], these methods have limited utility for the many enhancers with tissue-specific activity [45, 46] or low sequence conservation in spite of high functional conservation [39]. Yet quantifying enhancers’ conservation can provide insight into their functional relevance, such as the identification of convergent evolution in gene regulation underlying complex traits [21, 32, 97]. Here, we developed a machine learning-based approach to measure tissue-specific enhancer conservation. First, we trained CNNs to predict OCR orthologs’ open chromatin statuses in the tissue from which the OCRs were obtained. Then, we applied our models to predict the conservation of over 100,000 OCRs from each of brain and liver in over 200 mammals.

Our approach vastly outperformed nucleotide alignment-based methods of conservation PhastCons [13] and PhyloP [14] at predicting lineage- and tissue-specific open chromatin status. As expected, we found many examples where nucleotide-level conservation is low but the predicted open chromatin in a tissue of interest is conserved [39]. Conversely, we also identified cases where the nucleotide-level conservation is high, but the few differences between species disrupt open chromatin in a tissue of interest. Beyond out-performing conservation scores, our machine learning models’ predictions enabled us to do analyses that require direct comparisons between pairs of species other than human and mouse for which conservation scores are not available. We attribute the success of our method to our CNNs’ abilities to learn a conserved regulatory code linking genome sequence to tissue-specific open chromatin [57].

Our method builds upon recently published approaches that constructed machine learning models that learned conserved tissue-specific regulatory codes across species [57–60]. This work, however, had a different goal from ours. For instance, many previous studies for predicting enhancer activity in different species aimed to obtain the best performance on average across all enhancers in multiple tissues or cell lines [59, 98]. In contrast, our goal was to evaluate the feasibility of obtaining a model that can predict enhancer activity conservation or divergence across many species in a tissue of interest with decent accuracy, and such a model may not have the best overall performance (Supplemental Fig. 1b-i). For example, a study predicting liver open chromatin using SVMs showed overall high accuracy but was not able to accurately predict cases where sequence differences between primates were associated with regulatory differences [63]. Fully achieving our goal requires us to accurately make predictions in large numbers of species, including species with highly fragmented assemblies, making methods requiring long input DNA sequences, such as a previous method requiring over 100 kb input sequences [59], infeasible for making predictions at most enhancer orthologs. To demonstrate our ability to predict regulatory conservation, we developed a new, systematic set of evaluation criteria that evaluates if lineage- and tissue-specific differences can be predicted (Fig. 1b, Table 1). We used these criteria to compare the performance of machine learning models trained with different negatives and conservation scores. We note that, for some evaluation sets, we have a small number of examples (Supplemental Table 21) due to the conservative definitions we needed to use for defining them (Supplemental Notes), limiting our confidence in model performance. Since multiple machine learning models worked well according to most of our criteria, an exciting extension would be to train models on combinations of different types of negatives, which would likely require more training time but may improve performance. This is one of many extensions to our work that our criteria can help to evaluate (Supplemental Notes).

### Limitations of our approach

Although our models excel in many criteria for their stated purpose of predicting lineage- and tissue-specific open chromatin, all our models, including our best, perform sub-optimally for some of our criteria. Beyond model performance, our models’ set-up also has inherent limitations that prevent them from fully fulfilling their purposes. For example, many OCRs’ open chromatin statuses are influenced by factors beyond the 250 base pairs in each direction of their summits, and the fixed-size inputs required by CNNs prevents us from modeling some of the long-range interactions that may influence open chromatin (Supplemental Notes) [99]. In addition, since we predict open chromatin status of only OCR orthologs, we cannot identify non-open chromatin-conserved OCRs whose orthologs are not OCRs in any species for which we have open chromatin data (Supplemental Notes). Likewise, we cannot make predictions for OCR orthologs that do not align to the OCRs we identified in an existing alignment, so we are likely to miss OCR orthologs that do not align due to poor alignment quality, which is more common for distantly related species, and we cannot make any predictions in species whose genomes are not in alignments that allow us to map their genomes to the species for which we have data. We also treat open chromatin status as binary, but, in bulk open chromatin data, open chromatin is a continuous signal (Supplemental Notes). Finally, our approach requires high-quality open chromatin or some measure of enhancer activity from at least two species and is likely to have stronger performance when more are included. Cases where the datasets are not matched for sex, age, time of day, assay type, or other factors could influence model performance and may explain the sub-optimal performance of our models on species-specific OCRs and non-OCRs (Supplemental Notes).

### Applications of inferring conservation of tissue-specific regulatory activity

In addition to being accurate, our models’ predictions revealed that many OCRs may have lineage-specific patterns of open or closed chromatin and that these patterns may be functional. We developed an approach to deciphering the putative lineage-specificity of candidate enhancers identified in other studies in which we clustered our OCRs using predicted open chromatin as features and identified clusters with more overlap with these candidate enhancers than expected by chance. Applying this approach revealed that mouse candidate enhancers activated during neuron firing [93] are enriched for overlapping clusters with predicted Murinae-specific open chromatin, and human candidate enhancers activated during neuron activity [94] are enriched for overlapping clusters with predicted Euarchonta-specific gains or Muroidea-specific losses of open chromatin. These results suggest that there may be Muroidea-specific enhancer signatures of Muroidea neuron activity. Although no study, to our knowledge, has evaluated this for large numbers of species, a few studies have compared neuron activity between mice and one or two Euarchonta and found striking differences, including lower spiking frequencies in fast-spiking mouse cortical neurons relative to human and rhesus macaque [100] and lack of expression of h-Channels in mouse excitatory neurons relative to human [101]. We found clusters with predicted lineage-specific open chromatin for additional linages, predicted lineage-specific losses in open chromatin for additional lineages, and predicted convergent gains and losses in open chromatin; investigating the roles of the OCRs in these clusters could be an exciting path towards identifying mechanisms underlying linage-specific differences between mammalian brains and livers. However, we do not have sufficient data to fully evaluate model performance on OCR orthologs in Laurasiatherian species, so clusters involving Laurasiatherian lineage-specific open or closed chromatin may be a result of inaccurate predictions.

Our approach has the potential to be applied to numerous other groups of species, cell types, and tissues because it does not require experimentally determined OCR data from more than a few species but rather requires only genomes, which are being generated in unprecedented quantities [4, 9, 10], from many species. Applying our approach to more species and tissues can enable us to use it in a forward genomics approach [102] to help identify the mechanisms underlying the evolution of the expression of genes of interest or of phenotypes that have evolved through gene expression. This can be done by identifying OCR orthologs whose changes in predicted open chromatin status correspond to changes in gene expression or phenotypes. Many multi-species gene expression datasets in tissues with data from an assay that can serve as a proxy for enhancers are publicly available [40, 67, 88, 103, 104], and more will likely be generated in the near future [105]. Additional multi-species single-cell RNA-seq datasets are being generated from some of these tissues [106, 107] and will likely soon be supplemented by single-cell ATAC-seq. Many of these tissues have been associated with phenotypes that have evolved through gene expression [32, 97], so we can use the gene expression data along with predictions from models like ours to gain insights how these phenotypes evolved. Such insights may also reveal mechanisms underlying diseases associated with these phenotypes [60].

## Conclusions

The lineage and tissue-specificity of many enhancers limits our ability to quantify the conservation of enhancers through nucleotide-level conservation scores. Therefore, rather than focusing on identifying cases where natural selection is acting on individual nucleotides, we used open chromatin data from tissues of interest to train CNNs for predicting open chromatin and showed that our predictions can reveal potential cases where natural selection operates to maintain the TF binding sites that are needed to regulate gene expression. We evaluated the success of our CNNs as well as nucleotide conservation-based and other machine learning-based methods using new criteria that we designed explicitly for evaluating open chromatin conservation prediction. We then used CNNs to predict brain and liver open chromatin conservation across mammals and found that candidate enhancers associated with neuron firing tended to overlap regions of predicted lineage-specific open or closed chromatin. Our approaches to quantifying enhancer conservation and evaluating methods for this task can be applied to any tissue or cell type with enhancer data available from multiple species. As this is, to our knowledge, the first study to predict open chromatin conservation across more than a few species, we anticipate that our work will serve as a foundation for identifying OCRs whose open chromatin conservation is involved in gene expression evolution, providing insights into transcriptional regulatory mechanisms underlying phenotypes that have evolved through gene expression and their associated diseases.

## Methods

### Constructing negative set with non-OCR orthologs of OCRs

Mouse liver open chromatin data generation, curation of all other open chromatin datasets, data processing, and positive set construction details are described in Supplemental Methods. We obtained the non-OCR orthologs of OCRs by obtaining the orthologs of OCRs in each other species for which we had data from the same tissue and filtered the orthologs to include only those that did not overlap open chromatin in the same tissue (Supplemental Fig. 1a). For example, mouse chr4:127,435,564–127,436,049 does not overlap any OCRs in mouse cortex or striatum, but its human ortholog, chr1:34,684,126–34,684,689, is an OCR in both cortex and striatum, so this mouse region was a member of our novel negative set. To ensure that we would have enough negatives for training the model, we created a less conservative set of OCRs, which we called “loose OCRs” (Supplemental Fig. 13). For each species, tissue combination, the loose OCRs are the “base peaks” (Supplemental Methods, Supplemental Fig. 13) that are non-exonic, at least 20 kb from a TSS, and at most 1 kb long (same criteria used in constructing the positive set) and intersect at least 1 peak from the pooled reads across replicates from each of other datasets that are used for the species, tissue combination; we obtained these loose OCRs using bedtools [108]. We defined the peak summit of a loose OCR to be the peak summit of the corresponding base peak.

We identified orthologs of our loose OCRs in each other species with open chromatin data from the same tissue using halLiftover [109] followed by HALPER [110]. We used these tools instead of liftOver [111] because they map regions using Cactus alignments [112], which, unlike the pairwise alignment liftOver chains, are reference-free, contain many species, and account for a wide range of structural rearrangements, including inversions. We ran halLiftover with default parameters on our loose OCRs and their peak summits using the Zoonomia version 1 Cactus alignment [11]. We then constructed contiguous loose OCR orthologs from the outputs of halLiftover by running HALPER with parameters -max_frac 2.0, -min_len 50, and -protect_dist 5. Finally, we used bedtools subtract with the -A option [108] to remove the loose OCR orthologs in each species that overlapped peaks from the pooled reads across replicates from any of the datasets from the same tissue in that species, a set that we refer to as “Union Pooled Peaks” (Supplemental Fig. 13).

For the models trained on only mouse sequences, we used only the mouse orthologs of loose OCRs in non-mouse species (Supplemental Fig. 1a, Supplemental Fig. 13). For the multi-species brain models, we used the mouse, human, macaque, and rat orthologs of loose brain OCRs from each of the other species, and for the multi-species liver models, we used the mouse, macaque, and rat orthologs of loose liver OCRs from each of the other species. When constructing negatives of the non-OCR orthologs of OCRs, we used sequence underlying the ortholog of the base peak summit ± 250 bp and that sequence’s reverse complement. Our negative:positive training set ratios were approximately 1.16:1 for the brain model trained on only mouse sequences, 0.704:1 for the liver model trained on only mouse sequences, 1.49:1 for the multi-species brain model, and 0.822:1 for the multi-species liver model (Table 2). Construction of additional negative sets is described in Supplemental Methods.

### Training mouse-only brain model

Construction of training, validation, and test sets are described in Supplemental Methods [122–128], and machine learning models are listed in Table 2. We used CNNs [71] for our machine learning model because they have achieved state-of-the-art performance in related tasks [61, 113, 114]; they can learn complex combinatorial relationships between sequences, which we know can play an important role in enhancer activity [51]; and they do not require an explicit featurization of the data, enabling them to learn yet-to-be-discovered sequence patterns that are important for enhancer activity. Our inputs were one-hot-encoded DNA sequences [70], and our outputs were probabilities of sequences being OCRs in the tissue for which the model was trained. We used smaller architectures than those used by many previous studies for related tasks [59, 61, 113, 114] because these previous studies were training multi-task models, where our models were single-task models and therefore likely needed to learn substantially fewer sequence signatures. We first tuned hyper-parameters for the brain model trained on only mouse sequences for which the training set negatives were our novel negative set by comparing the validation set performances of various models (Model Number 6 in Table 2, Fig. 1c). Specifically, we began with the architecture that we used for TF binding prediction in our previous work [115], a three-layer CNN with 15–60 filters of size 15 per layer. We then tuned hyper-parameters until we had a model that had satisfactory performance according to all our evaluation criteria on the validation set. [We constructed evaluation sets corresponding to the validation set in the same way as those for generating the results we present except that we used the mouse validation set chromosomes instead of the mouse test set chromosomes (Supplemental Methods).] We did not do an exhaustive hyper-parameter search for any model because our goal was to evaluate the feasibility of training models that had satisfactory performance for all our evaluation criteria and not to train optimally performing machine learning models.

Our final architecture was five convolutional layers with 300 filters of width 7 and stride 1 in each, followed by a max-pooling layer with width and stride 26, followed by a fully connected layer with 300 units, followed by a fully connected layer that went into a sigmoid. All convolutional layers had dropout 0.2 and L2 regularization 0.00001. We trained the model using stochastic gradient decent with learning rate 0.001, Nesterov momentum 0.99, and batch size 100, and each class was assigned a weight equal to the fraction of the other class in the training set. We trained the model using the training set until there were three consecutive epochs with no improvement in recall at eighty percent precision (or, if there were more positives than negatives, no improvement in specificity at eighty percent NPV) on the validation set. Before training, we initialized the weights to be those from a pre-trained neural network with the same hyper-parameters and the negative set randomly down-sampled to be the size of the positive set (or a positive set randomly down-sampled to be the size of the negative set if the positive set was larger) in order to help the model handle the class imbalance, as we did in our previous work [115]. We initialized the weights for the pre-training using Keras’s He normal initializer [116, 117]. We started with these hyper-parameters for training our other models and then tuned them as described in Supplemental Methods.

### Evaluating machine learning models

In addition to evaluating models on the test sets corresponding to the training sets, we evaluated models on multiple additional evaluation sets to compute their lineage-specific and tissue-specific OCR accuracies. Illustrations of clade-specific and species-specific OCRs for the lineage-specific OCR accuracy evaluations and tissue-specific OCRs for the tissue-specific OCR accuracy evaluations are in Fig. 1b. A list of evaluation sets is in Supplemental Table 20, the numbers of positives and negatives in each evaluation set are listed in Supplemental Table 21, and the figures illustrating the performance for each evaluation set, model combination are listed in Supplemental Table 22. Processing of liver H3K27ac ChIP-seq data for comparing predicted open chromatin conservation to conservation of H3K27ac ChIP-seq is described in Supplemental Methods.

#### Evaluating models’ lineage-specific OCR accuracy

##### OCR orthologs with differing open chromatin statuses between two species

To obtain OCR orthologs that are open in one species but not in another, we used as positives the sequences and reverse complements of sequences underlying OCRs from one species whose orthologs in the other species have closed chromatin and as negatives the sequences and reverse complements of sequences underlying non-OCRs whose orthologs in at least one other species are OCRs. More specifically, to construct positives for the MouseBr ≠ OtherBr and MouseLv ≠ OtherLv evaluations, we used halLiftover [109] followed by HALPER [110] with the same parameters we used previously to identify test set chromosome mouse OCR orthologs in human, macaque, and rat for brain and macaque and rat for liver. We then removed peaks that overlapped the of union pooled peaks from any dataset in the same tissue (Supplemental Fig. 13, Supplemental Tables 20–21). To construct negatives for these evaluations, we used the approach for constructing our novel negative set except that we identified non-OCR orthologs of OCRs instead of loose OCRs (Fig. 1c, Supplemental Fig. 1c, Supplemental Fig. 8b, Supplemental Tables 20–21). To construct positives for the MouseBr ≠ RatBr and MouseLv ≠ RatLv evaluations, we used the subset of regions from MouseBr ≠ OtherBr and MouseLv ≠ OtherLv, respectively, whose orthologs’ open chromatin status differ between mouse and rat (Fig. 1c, Supplemental Fig. 1d, Supplemental Fig. 8b, Supplemental Fig. 13, Supplemental Tables 20–21). To construct positives for the MacaqueBr ≠ MouseBr and MacaqueLv ≠ MouseLv evaluations, we identified macaque OCRs whose mouse orthologs do not overlap any of the union pooled peaks in the same tissue (Supplemental Fig. 1f, Supplemental Fig. 8b, Supplemental Fig. 13) and are on test set chromosomes. To construct negatives for these evaluations, we identified mouse test set chromosome OCR orthologs in macaque that do not overlap any of the union pooled peaks in the same tissue (Supplemental Fig. 1f, Supplemental Fig. 8b, Supplemental Fig. 13, Supplemental Tables 20–21). We obtained regions for HumanBr ≠ MouseBr, RatBr ≠ MouseBr, and RatLv ≠ MouseLv using same process that we used for MacaqueBr ≠ MouseBr and MacaqueLv ≠ MouseLv (Fig. 1c, Supplemental Fig. 1g, Supplemental Fig. 1h, Supplemental Fig. 8b, Supplemental Tables 20–21).

##### Clade- and species-specific OCRs

We defined a clade-specific OCR in a tissue as an OCR whose ortholog is open in that tissue in every species within a clade for which we have data and closed in that tissue in every other species for which we have data, and we defined a species-specific OCR in a tissue as an OCR whose ortholog is open in that tissue in a species for which we have data and is closed in that tissue in the single most closely related species (there were no ties) for which we have data (Fig. 1b). Laurasiatheria data was used for only comparing mouse-only to multi-species liver models (Fig. 3e). More specifically, we identified clade-active OCRs in each clade – OCRs in a “base species” whose ortholog in the other species in that clade (if there was another) overlaps an open chromatin peak from the pooled reads across replicates in all the datasets we used in that tissue from that species (Supplemental Fig. 13). We did not require the OCR ortholog in the non-base species to overlap a reproducible open chromatin peak so that we could have at least one hundred test set examples for each evaluation. We chose the “base species” to be the species in each clade with the highest-quality genomes – mouse for Glires for brain and liver, human for Euarchonta for brain, macaque for Euarchonta for liver, and cow for Laurasiatheria for liver. We then identified the subset of clade-active peaks from the base species whose orthologs in all species in the other clade do not overlap any open chromatin peaks from the union pooled peaks; these were our clade-specific OCRs (Supplemental Fig. 13). To obtain clade-specific non-OCRs for a clade, we identified orthologs of clade-active OCRs from the other clade in the base species in the clade whose orthologs in all species in the clade did not overlap open chromatin peaks from union pooled peaks (Fig. 1b, Supplemental Fig. 13). For the evaluations we present, we used only test set chromosomes for mouse and regions whose mouse orthologs are on test set chromosomes for other species. The sequences of clade-specific OCRs and non-OCRs used for evaluating the models were those from the base species and their reverse complements (Fig. 1c, Figs. 3a-b, Fig. 3e, Supplemental Fig. 1e, Supplemental Fig. 1i, Supplemental Figs. 4a-b, Supplemental Fig. 8b, Supplemental Tables 20–21). When evaluating the multi-species models, we combined the clade-specific OCRs and non-OCRs from Euarchonta and Glires (Supplemental Table 21).

The species-specific OCRs and non-OCRs are described in Supplemental Table 20 (Fig. 1b). For the results we present, we used only regions on test set chromosomes for mouse and only regions whose mouse orthologs are on test set chromosomes for other species (Fig. 3a, Supplemental Table 21). We did not include macaque-specific OCRs and non-OCRs when evaluating the multi-species liver model because we did not have liver open chromatin from any other Euarchonta species. We combined the species-specific OCRs and non-OCRs from different species when evaluating the multi-species models (Fig. 3a-b, Supplemental Table 21).

#### Evaluating models’ tissue-specific OCR accuracy

To evaluate the performance of models trained in one tissue on OCRs from another tissue, we defined our positives to be OCRs that are shared between the two tissues (shows our models were learning more than just the sequences involved in tissues-specific open chromatin), and we defined our negatives to be OCRs in the evaluation tissue that do not overlap OCRs in the training tissue (shows our models were learning more than just the sequences involved in general open chromatin) (Fig. 1b). For the evaluations we present, we used only regions on test set chromosomes for mouse and regions whose mouse orthologs are on test set chromosomes for other species. We used bedtools intersectBed with options -wa and -u [108] to identify OCRs from our training tissue that overlap OCRs from the evaluation tissue, where we included OCRs longer than 1 kb for the evaluation tissue for mouse (Supplemental Fig. 5, Supplemental Fig. 8c, Supplemental Tables 20–21). We used bedtools subtractBed with option -A [108] to identify liver OCRs that do not overlap any brain union pooled peaks and brain OCRs that do not overlap open chromatin from liver union pooled peaks (Fig. 1b, Supplemental Fig. 5, Supplemental Fig. 8c, Supplemental Fig. 13, Supplemental Tables 20–21). We combined the data from all three species for evaluating the multi-species models (Figs. 3a-b, Supplemental Table 21).

For comparing models trained with different training sets, we also compared the distributions of test set chromosome predictions for the brain OCRs that do not overlap liver OCRs, the brain OCRs that overlap liver OCRs, the liver OCRs that do not overlap brain OCRs (Fig. 1b), and the negative set. We defined these groups of OCRs as we did for other evaluations, and we used predictions for sequences and their reverse complements. We compared the distributions for the brain OCRs that overlap liver OCRs to the liver OCRs that do not overlap brain OCRs using a Wilcoxon rank-sum test and multiplied the p-values by 6 to do a Bonferroni correction.

#### Evaluating if models’ predictions had phylogeny-matching correlations

To evaluate the relationship between OCR ortholog open chromatin status and phylogenetic distance, we identified test set mouse OCR orthologs in all the fifty-six Glires species from Zoonomia, predicted the open chromatin statuses of those orthologs, and computed the correlation between those predictions and the species’ phylogenetic divergences from mouse. This provides us with an approximate measure of how predicted OCR ortholog open chromatin statuses change over evolution. Specifically, we identified the mouse test chromosome OCR orthologs and OCR summit orthologs in Glires using halLiftover [109] with the Zoonomia version 1 Cactus alignment [11, 112]; we used brain OCRs for evaluating brain OCR models and liver OCRs for evaluating liver OCR models. We next constructed contiguous orthologs from the outputs of halLiftover using HALPER [110] with parameters -max_frac 2.0, -min_len 50, and -protect_dist 5. We constructed inputs for our models from the contiguous OCR orthologs by using bedtools fastaFromBed [108] with fasta files downloaded from NCBI [4, 118] and the UCSC Genome Browser [119] to obtain the sequences underlying their summit orthologs ± 250 bp. We constructed the reverse complements of sequences, used our models to predict each sequence and its reverse complement’s open chromatin status, and averaged the predictions between the forward and reverse strands. We then removed all predictions from OCRs with orthologs in less than one quarter of species. After that, for each model, we computed the mean OCR ortholog open chromatin status prediction and the standard deviation of predictions across all remaining OCR orthologs in each species. We finally computed the Pearson and Spearman correlations between these means and standard deviations of predictions and the millions of years since divergence from mouse, which we obtained from TimeTree [120]. We did this for brain OCR orthologs using brain models trained with each training set as well as for liver OCR orthologs using the liver model trained on only mouse sequences and the multi-species liver model (Fig. 3, Supplemental Figs. 7–8, Supplemental Fig. 10).

### Comparing predictions to mean conservation scores

We compared the predictions to mean conservation scores by identifying OCR orthologs with conserved and non-conserved open chromatin status between species, computing the mean conservation scores of those OCR orthologs, and comparing those scores to the predicted open chromatin status of those OCR orthologs. We defined an OCR ortholog with conserved open chromatin status between mouse and another species as a mouse OCR whose ortholog in the other species overlaps an OCR in the same tissue. For mouse brain test chromosome OCRs, we identified 441 OCRs with conserved open chromatin status in macaque, 195 OCRs with conserved open chromatin status in human, and 670 OCRs with conserved open chromatin status in rat. For mouse liver test chromosome OCRs, we identified 689 OCRs with conserved open chromatin status in macaque and 580 OCRs with conserved open chromatin status in rat. We defined an OCR ortholog with non-conserved open chromatin status between mouse and another species as a mouse OCR whose ortholog in another species does not overlap any union pooled peaks in that tissue in that species (Supplemental Fig. 13). For mouse brain test set OCRs, we identified 394 OCR orthologs with non-conserved open chromatin status in macaque, 448 OCR orthologs with non-conserved open chromatin status in human, and 338 OCR orthologs with non-conserved open chromatin status in rat. For mouse liver test set OCRs, we identified 1,114 OCR orthologs with non-conserved open chromatin status in macaque and 1,241 OCR orthologs with non-conserved open chromatin status in rat. We think that the differences in numbers of OCR orthologs with conserved and non-conserved open chromatin status between species is due not only to differences in evolutionary relatedness but also to differences between species in numbers of datasets used to define OCRs and differences in sequencing depths of those datasets [41, 64, 68].

We used our models to predict the OCR ortholog open chromatin status for the open chromatin status-conserved and open chromatin status non-conserved OCR orthologs in the non-mouse species and compared it to the conservations scores of the mouse OCRs. We computed mean conservation scores of the mouse OCRs by calculating the mean PhastCons [13] and PhyloP [14] scores at the peak summits ± 250 bp. We evaluated if the distributions of the predictions and each type of conservation score differed between the open chromatin status-conserved and open chromatin status non-conserved orthologs using a Wilcoxon rank-sum test. We did a Bonferroni correction by multiplying all p-values by 20 (2 conservation score comparisons and 2 model predictions comparisons – models trained on only mouse sequences and multi-species models – for 5 species, tissue pairs; Supplemental Tables 7–8).

We then evaluated whether the predictions were more effective than the mean conservation scores at differentiating between open chromatin status-conserved and open chromatin status non-conserved OCR orthologs. We first averaged the predictions of the sequence underlying the non-mouse OCR ortholog’s summit ± 250 bp and its reverse complement so that each OCR ortholog would have a single prediction value. We next combined our open chromatin status-conserved and open chromatin status non-conserved OCR orthologs and ranked them according to each of PhastCons score, PhyloP score, and OCR ortholog open chromatin status prediction. We then did a Wilcoxon sign-rank test to compare the ranking distributions of the open chromatin status-conserved OCR orthologs between the OCR ortholog open chromatin status predictions and each type of conservation score. We also did this for the ranking distributions of the open chromatin status non-conserved OCR orthologs. We did this for predictions made by the models trained using only mouse sequences and by the multi-species models. Finally, we did a Bonferroni correction by multiplying all p-values by 40 (2 conservation score comparisons for each of mouse-only and multi-species models for open chromatin status-conserved and open chromatin status non-conserved OCR orthologs for 5 species, tissue pairs; Supplemental Table 16).

### Clustering OCRs

To generate OCR clusters for OCRs from a tissue, we mapped the OCRs from each species across all the Boreoeutheria from Zoonomia [4, 11] except for *Manis tricuspis*, filtered the OCRs, and clustered the OCRs. Specifically, we mapped OCRs from each species with OCRs using halLiftover with the Zoonomia version 1 Cactus [11] and constructed contiguous orthologs from halLiftover outputs using HALPER [110] with settings -max_frac 2.0, -min_len 50, and -protect_dist 5. We then used the multi-species model corresponding to the tissue for which the OCR was generated (in Table 2, model 8 for brain and model 9 for liver) to make predictions for the OCR orthologs and OCR orthologs’ reverse complements in all the Boreoeutheria that we mapped to except for *Galeopterus variegatus*, *Hippopotamus amphibius*, *Monodon monoceros*, and *Platanista gangetica*. For each OCR ortholog, we set the prediction to be the average between the prediction for the ortholog and the prediction for its reverse complement.

We filtered OCRs to ensure that we did not have redundant OCRs and to ensure that we had usable OCR orthologs in enough species to use predictions in each species as features for clusters. First, for brain, we removed all human OCRs whose mouse ortholog overlapped a mouse brain OCR, all macaque OCRs whose mouse ortholog overlapped a mouse brain OCR or human ortholog overlapped a human brain OCR, and all rat OCRs whose mouse ortholog overlapped a mouse brain OCR, human ortholog overlapped a human brain OCR, or macaque ortholog overlapped a macaque brain OCR. For liver, we removed all macaque OCRs whose mouse ortholog overlapped a mouse liver OCR and all rat OCRs whose mouse ortholog overlapped a mouse liver OCR or whose macaque ortholog overlapped a macaque liver OCR. After that, we removed all remaining OCRs that did not have a usable ortholog in at least half of all species or at least one quarter of each of Euarchonta, Glires, and Laurasiatheria.

We clustered the remaining OCRs using the prediction of each species’ OCR ortholog’s open chromatin status as a feature and treating species without usable orthologs as missing data. We first used k-means clustering with k = 9,000 (brain) or 12,000 (liver) to cluster the OCRs into small clusters; we defined cluster centroid values for each species as the mean of all OCR ortholog activity predictions for that species in the cluster. We did this because affinity propagation clustering was not tractable for hundreds of thousands of examples with hundreds of features. We then used affinity propagation clustering [121], implemented in scikit-learn [122], with preference = -0.6 to cluster the outputs of the k-means clustering, which we defined as the centroids of the small clusters, into larger clusters. In both clustering steps, we defined the distance between two OCRs as 1 minus the cosine similarity between the vectors of enhancer activity predictions in the species for which both had usable orthologs. We ultimately obtained 102 brain clusters and 103 liver clusters.

### Identifying enhancer sets with more overlap with clusters than expected by chance

To identify enhancer sets with more overlap with a cluster than expected by chance, we first obtained the enhancer sets from the supplemental information of the relevant manuscripts. For the mouse neuron firing candidate enhancers, we used the regions in Tables S11—S13 from a recent study of enhancers activated during mouse neuron firing [93] and used liftOver [111] to map the coordinates from mm9 to mm10. For the human GABAergic neuron activity candidate enhancers, we used the regions in Supp Data 12 from a recent study of enhancers activated during human neuron activation [94] and used liftOver to map the coordinates from hg19 to hg38 [111]. For the mouse liver regeneration candidate enhancers, we used various subsets of regions from Supplemental Table  1 from a recent study of enhancers activated during liver regeneration (Supplemental Table 19). For each category of regions in a header in Supplemental Table 19, we required the FDR to be less than 0.05 and the log2 fold-change to be greater than 1 (for ↑) or less than 1 (for ↓). We then used LOLA [123] to run a hypergeometric test to evaluate the statistical significance of the overlap of each enhancer set with each cluster from the relevant tissue that is open in the relevant species; our query was our enhancer set, our database was our list of bed files with the relevant cluster locations, our universe was our list of OCRs in the relevant species, tissue combination, and we set “redefineUserSets” to TRUE. We did a Bonferroni correction by multiplying all p-values by 391, which was the total number of tests.

We wanted to determine if our enrichment for overlaps with clusters with lineage-specific open chromatin or lack of open chromatin could be partially explained by OCRs overlapping enhancer sets having fewer usable orthologs or lower conservation. To evaluate this, we used bedtools [108] to identify the OCRs from each relevant tissue, species combination overlapping each enhancer set. We next computed the number of usable orthologs, the average PhastCons [13] score for the peak summit ± 250 bp, and the average PhyloP [14] score for the peak summit ± 250 bp for all OCRs from the relevant tissue, species combination as well as for the OCRs overlapping each enhancer set. Then, for each enhancer set, we used a Wilcoxon rank-sum test to compare the numbers of usable orthologs and the PhastCons and PhyloP scores between the OCRs overlapping the enhancer set and the full set of OCRs. We considered the difference to not be statistically significant if the nominal p-value was greater than or equal to 0.05.

## Supplementary Information

**Additional file 1. **

## Data Availability

Human DNase hypersensitivity data analyzed in this study was downloaded from the ENCODE portal (https://www.encodeproject.org/) [41, 67], and human ATAC-seq data analyzed in this study was downloaded from Gene Expression Omnibus accession GSE96949 [68]. Macaque and rat ATAC-seq data from data we previously generated [64] and mouse liver ATAC-seq data in this manuscript can be accessed in Gene Expression Omnibus accession GSE159815. Publicly available mouse brain data analyzed in this study can be accessed in Gene Expression Omnibus accession GSE161374. Publicly available mouse liver ATAC-seq data was downloaded from China National Gene Bank accession CNP0000198 [124]. Other mouse ATAC-seq data was downloaded from China National Gene Bank accession CNP0000198 [124] and the ENCODE portal (https://www.encodeproject.org/) [74]. Cow and pig ATAC-seq data was obtained from the authors of [76]. Mouse and macaque H3K27ac ChIP-seq data was downloaded from ArrayExpress accession E-MTAB-2633 [40]. Code and cluster images can be found at https://github.com/pfenninglab/OCROrthologPrediction, and additional code and cluster information can be obtained from the authors upon request.
