# Supplementary material for: Inferring mammalian tissue-specific regulatory conservation by predicting tissue-specific differences in open chromatin
Source: BMC Genomics. 2022 Apr 11;23:291. doi: 10.1186/s12864-022-08450-7 (PMC8996547; doi:10.1186/s12864-022-08450-7)

Supplemental Information for Inferring mammalian tissue-specific regulatory conservation by predicting tissue-specific differences in open chromatin

CONTENTS

1. Supplemental Notes
2. Supplemental Materials and Methods
3. Supplemental Figure Captions
4. Supplemental Tables
5. Supplemental References

SUPPLEMENTAL NOTES

Preliminary Model Evaluation

We evaluated the machine learning model trained on each training set on its corresponding test set. We found that all models worked well, with every model achieving an AUPRC > 0.7 (**Supplemental Figure 1b**). This performance is especially impressive given that the ratios of the number of negatives to the number of positives ranged from approximately 1.2:1 (non-OCR orthologs of OCRs) to approximately 20:1 (OCRs in other tissues). The best-performing model was the model with the training set whose negatives consisted of dinucleotide-shuffled brain OCRs (**Supplemental Figure 1b**). However, in this comparison, each model was evaluated on a test set with different negatives, so this evaluation may not be indicative of how useful each model would be in answering questions about gene expression evolution.

Re-Calibrating Models with Our Negative Set Usually Does Not Substantially Improve Performance

Since, for many applications, we need to make a binary classification as to whether a region is open in brain, we also investigated how well-calibrated our models are. We found that models trained with some training sets — including those with flanking region negatives, OCRs in other tissue negatives, the smaller number of G/C- and repeat-matched region negatives, and dinucleotide-shuffled brain OCR negatives — tended to do better on clade-specific OCRs than on clade-specific closed chromatin regions (**Figure 1b, Supplemental Figure 1a**). On the other hand, the models whose negatives were the larger number of G/C- and repeat-matched regions and our novel negative set tended to do better on clade-specific closed chromatin regions than on clade-specific OCRs. We tried re-calibrating all the models with the training set positives and negatives from our novel negative set. For the models trained on all training sets except for the one with our novel negative set, this led to an increase in specificity and a decrease in

sensitivity. In general, the increase in specificity was not much greater than the decrease in sensitivity (**Supplemental Tables 1-6**), but for model whose training set negatives were the smaller number of G/C- and repeat-matched regions, the increase in specificity was substantially larger than the decrease in sensitivity (**Supplemental Figure 2**). Thus, while some models were poorly calibrated, re-calibrating models with the positives and negatives from our novel negative set usually had limited utility.

<u>Machine Learning Models Predict OCR Orthologs' Open Chromatin Status Significantly More Accurately than Mean Conservation Scores</u>

To do quantify the extent to which our machine learning model whose training set negatives were our novel negative set can predict differences in open chromatin conservation relative to conservation scores, we identified test set mouse brain and liver OCRs whose macaque orthologs do and do not overlap OCRs in brain and liver, respectively, and computed the mean conservation scores of these OCRs [1, 2] as well as the predictions on test set macaque orthologs of the machine learning models whose training set negatives were our novel negative set in the corresponding tissue. We found that mean conservation scores and model predictions tended to be higher for the macaque orthologs for which open chromatin status was conserved than those for which open chromatin status was not conserved (**Supplemental Tables 7-8**). For each tissue, we then ranked the macaque OCR orthologs based on their mean conservation scores and their model predictions, with the highest rank corresponding to the highest score or open chromatin status prediction. For the open chromatin status-conserved OCRs in each tissue, we used a Wilcoxon signed-rank test to evaluate whether these OCRs tended to have higher ranks for our predictions than they do for mean conservation scores; we found that the ranks were significantly higher for our predictions (brain predictions vs. PhastCons scores: 3.69×10^{-4}, brain predictions vs. PhyloP scores: 9.30×10^{-6}, liver predictions vs. PhastCons scores: 6.25×10^{-17}, liver predictions vs. PhyloP scores: 1.35×10^{-22}). For each tissue, we also used a Wilcoxon signed-rank test to evaluate whether the OCR orthologs

without open chromatin tended to have lower ranks for our predictions than they do for mean conservation scores, and we found that the ranks were significantly lower for our predictions (brain predictions vs. PhastCons scores: 6.88×10^{-4}, brain predictions vs. PhyloP scores: 1.85×10^{-7}, liver predictions vs. PhastCons scores: 3.63×10^{-11}, liver predictions vs. PhyloP scores: 3.42×10^{-18}). We repeated this for human and rat orthologs of mouse brain OCRs with conserved and non-conserved open chromatin statuses and for rat orthologs of mouse liver OCRs with conserved and non-conserved OCR statuses and obtained similar results (**Supplemental Tables 7-9**). This shows that using machine learning models for predicting open chromatin status conservation of OCR orthologs can be more accurate than using mean sequence conservation scores.

Machine Learning Models Learned Motifs of Brain Transcription Factors

To determine what sequence patterns our models were prioritizing, we ran DeepLIFT with the rescale rule [3] followed by TF-MoDISco [4] on the positive examples from the validation set from each model and compared the results to known motifs. All the models seemed to have learned motifs of TFs that are known to play important roles in the brain, including Ctcf [5, 6], Fos [7-9], Egr2 [10, 11], and Rfx4 [12-14] (**Supplemental Figure 6**). All the models except for those trained with flanking region negatives (**Supplemental Figure 6a**) and those trained with dinucleotide-shuffled brain OCR negatives (**Supplemental Figure 6e**) seemed to also have learned the motif of Mef2c, a TF with multiple roles in the brain [15-17] (**Supplemental Figure 6**). The model trained with OCRs in other tissue negatives seemed to have learned the depletion of motifs of multiple TFs whose mouse and human orthologs are not expressed in the assayed brain regions most similar to those in our study, including Hnf4g, Nr5a1, Elf3, and Foxd2, and the model with model trained with the larger number of G/C- and repeat-matched region negatives seemed to have learned a depletion of the motif for Nr2f6 [18-20] (**Supplemental Figures 6b-c**). The model trained with dinucleotide-shuffled brain OCR negatives seemed to have learned the motif for Bcl6

[21, 22], but this motif consists almost exclusively of G's, so it might be indicative of many consecutive G's being more common in brain OCRs than in shuffled brain OCRs (**Supplemental Figure 6e**). The model trained with our novel negative set also seemed to have learned the motif for Dbp, which has been implicated in circadian rhythms [23, 24]; two slightly different Rfx motifs (also learned by the model with the smaller number of G/C- and repeat-matched region negatives), which is not surprising because multiple Rfx TFs play important roles in the brain [12, 14, 25]; and a depletion of the motif for Thra (**Supplemental Figures 6d, f**). It is possible that these apparent differences in motifs learned by the models caused their differences in performance.

Phylogeny-Matching Correlation Evaluations for Brain Models Trained on Mouse Sequences

Our approach for determining whether our models' predictions have phylogeny-matching correlations does not require open chromatin data from multiple species. We obtained the orthologs of the mouse brain OCRs in all of the fifty-six Glires species in the Zoonomia Project [26, 27], used our machine learning models to predict the brain open chromatin statuses of these orthologs, computed the mean brain open chromatin statuses across all brain OCR orthologs in each species, and computed the correlation between mean predicted brain open chromatin status and evolutionary distance from mouse. Although there are OCR orthologs, such as species-specific OCRs and OCRs with convergently evolved open chromatin [28], whose open chromatin conservation across species is not associated with phylogenetic distance, we think that such OCRs in most tissues are a minority due to the principle of evolutionary parsimony and a previous study of enhancer activity across multiple species [29]. As anticipated, all models showed a strong negative correlation between mean predicted brain open chromatin status and divergence from mouse (**Supplemental Figure 7a**). Nevertheless, there is still more open chromatin at these brain OCR orthologs than would be expected from brain non-OCRs, even in the most distantly related Glires species, because all mean predictions are greater than the mean predictions

for the negatives in the test sets corresponding to the training sets (**Supplemental Figure 7a**). We also expected there to be a strong positive correlation between the standard deviation of open chromatin status and divergence from mouse because most brain OCR orthologs in species closely related to mouse are active in the brain, while the brain open chromatin status of brain OCR orthologs in species that are more distantly related should vary. We found this expected positive correlation for the predictions from all our machine learning models trained on mouse data (**Supplemental Figure 7b**).

Approach to Evaluating Machine Learning Models for OCR Ortholog Open Chromatin Status Prediction Can Be Applied to Any Tissue or Cell Type with Open Chromatin Data from Multiple Species

Although we prototyped our approach to evaluating machine learning models for predicting open chromatin status of OCR orthologs in the brain, this approach can be applied to any tissue or cell type with open chromatin data from multiple species. We therefore applied it to another tissue, the liver, and found that our novel approach to constructing training set negatives also worked well for most metrics. To do this, we first generated a new mouse liver open chromatin dataset and found that it was high-quality (**Supplemental Figure 8a**, TSS enrichment for replicate 1 = 17.27, TSS enrichment for replicate 2 = 16.31, rescue ratio = 1.02, self-consistency ratio = 1.26). We then defined our positive set for liver as the 250bp in each direction of peak summits of our mouse liver ATAC-seq peaks that overlapped liver ATAC-seq peaks from CNP0000198 (**Supplemental Figure 13**) [30]. We obtained negatives by mapping rat and macaque liver ATAC-seq data from the Pfenning Lab [31] to mouse and identifying the mouse orthologs that did not overlap mouse liver union pooled peaks (**Figure 1b, Supplemental Figure 13**). We found that the model achieved high lineage-specific and tissue-specific OCR accuracy (AUPRC > 0.65, **Supplemental Figures 8b-c**). We also determined if our predictions had phylogeny-matching correlations by obtaining orthologs of the mouse liver OCRs in all the Glires from the Zoonomia project [26, 27] and predicting their open chromatin statuses. As with brain, we found a strong negative correlation between the predicted

mean liver OCR ortholog open chromatin status in Glires and those species' divergence from mouse (**Supplemental Figure 8d**) and a strong positive correlation between standard deviation of predicted liver OCR ortholog open chromatin status in Glires and those species' divergence from mouse (**Supplemental Figure 8e**). In addition, we interpreted the model using DeepLIFT with the rescale rule [3] followed by TF-MoDISco [4] and found that the model seemed to have learned motifs of multiple known liver TFs, including Hnf4a [32-34], Ctcf [5, 35], Cebpa [36, 37], Onecut1 [38, 39], and Foxa2 [40-42], as well as a depletion of the motif for Wt1, whose mouse and human orthologs are not expressed in liver, and for Plagl1, whose mouse and human orthologs have low expression in liver [18-20] (**Supplemental Figure 8f**).

Comparison of Liver Model Predictions to H3K27ac ChIP-seq Conservation

For each species with open chromatin data, we identified OCRs overlapping H3K27ac ChIP-seq peaks from https://www.ebi.ac.uk/research/flicek/publications/FOG15 [43] and compared the multi-species liver model open chromatin predictions in each other species with H3K27ac ChIP-seq data for the orthologs with H3K27ac ChIP-seq to those for the orthologs without H3K27ac ChIP-seq. We found that the predictions for OCR orthologs for which H3K27ac ChIP-seq was conserved tended to be higher than the predictions for OCR orthologs for which H3K27ac ChIP-seq was not conserved (**Supplemental Table 13**). This illustrates that our novel approach to constructing training set negatives for open chromatin status prediction of OCR orthologs works well in multiple tissues.

Open Chromatin Predictions Do Not Seem to Be Associated with Genome Quality

Since the Zoonomia genomes vary in quality, we evaluated whether our open chromatin status predictions are associated with genome quality [26, 27, 44]. We computed the correlation between mean predicted brain open chromatin status across the mouse brain OCR orthologs in each Glires species and scaffold and contig N50's. We found a weak Pearson correlation and even weaker Spearman correlation

between the scaffold and contig N50's and the mean predicted brain open chromatin status (**Supplemental Figure 11a**). We repeated this process in for the liver predictions of the mouse liver OCR orthologs and obtained similar results (**Supplemental Figure 11b**). To demonstrate that mean predicted mouse OCR ortholog open chromatin status has a stronger relationship with divergence from mouse than it does with genome quality, we created generalized linear models for mean predicted mouse OCR ortholog open chromatin status with covariates for divergence from mouse and scaffold or contig N50. The coefficients for divergence from mouse were all statistically significantly different from zero and larger in magnitude than the coefficients for scaffold or contig N50, and the coefficients for scaffold or contig N50 were never statistically significantly different from zero (**Supplemental Table 14**). These results suggest that lower-quality genomes are not strongly associated with lower OCR ortholog open chromatin status predictions.

To further evaluate the relationship between genome quality and our predictions, we investigated whether the extent to which OCR ortholog open chromatin status predictions vary within a species is associated with genome quality. To do this, we computed the correlation between standard deviation of predicted brain open chromatin status across the mouse brain OCR orthologs in each of the Glires and scaffold and contig N50's. We found a weak negative Pearson correlation and even weaker negative Spearman correlation between the scaffold N50's and the standard deviation of predicted brain open chromatin status; for contig N50, the Pearson correlation was weak and negative, while the Spearman correlation was weak and positive (**Supplemental Figure 11c**). We repeated this process for the liver open chromatin status predictions of the mouse liver OCR orthologs and obtained similar results except that the Spearman correlation between the contig N50 and standard deviation of predicted open chromatin status was weak and negative (**Supplemental Figure 11d**). To demonstrate that standard deviation of predicted mouse OCR ortholog open chromatin status had a stronger relationship with divergence from mouse than it did with genome quality, we created generalized linear models for standard deviation of

predicted mouse OCR ortholog open chromatin status with covariates for divergence from mouse and scaffold or contig N50. The coefficients for divergence from mouse were all statistically significantly different from zero and larger in magnitude than the coefficients for scaffold or contig N50 (**Supplemental Table 15**). These results further demonstrate that genome quality does not substantially influence our OCR ortholog open chromatin status predictions.

Multi-Species Models Learn Additional Brain and Liver TF Motifs

We found that our multi-species brain and liver models seemed to have learned motifs of brain and liver TFs, respectively. When interpreting the multi-species brain model, in addition to the motifs that we found for the model trained on only mouse sequences, we found a depletion of the motifs for Nr1i3 and Pit1, whose mouse and human orthologs are not expressed in the assayed brain regions most similar to those in our study (**Supplemental Figure 10c**) [18-20]. When interpreting the multi-species liver model, in addition to the motifs that we found for the model trained on mouse sequences, we also found the motifs for additional TFs that are known to be involved in the liver, including Ppara [45-47], Ets2 [48, 49], Sp1 [50, 51], Bcl6 [52, 53], and Nfe2l2 [54, 55], as well as a depletion of the motif for Dbx1, whose mouse and human orthologs are not expressed in liver [18-20], and a depletion of the motif for Zfp637 (**Supplemental Figure 10d**). These results suggest that our multi-species models learned the importance of relevant sequence features to their tasks and not only learning general patterns of genome sequence content.

Multi-Species Machine Learning Models Make Significantly More Accurate Predictions than Mean Conservation Scores

We compared the test set predictions of our multi-species models to those made by mean conservation scores. First, we found that our model predictions for non-mouse orthologs of mouse brain

and liver OCRs whose open chromatin status is conserved tended to be higher than for non-mouse orthologs of mouse brain and liver OCRs whose open chromatin status is not conserved (**Supplemental Tables 7-8**). Then, for each tissue, we ranked the macaque OCR orthologs based on their model predictions, with the highest rank corresponding to the highest score or open chromatin status prediction. For the open chromatin status-conserved OCRs, we evaluated whether these OCRs tended to have higher ranks for our multi-species model predictions than they did for mean conservation scores; we found that the ranks were significantly higher for our predictions (brain predictions vs. PhastCons scores: 7.80×10^{-5}, brain predictions vs. PhyloP scores: 1.53×10^{-6}, liver predictions vs. PhastCons scores: 3.68×10^{-22}, liver predictions vs. PhyloP scores: 1.25×10^{-27}). For each tissue, we also evaluated whether the OCR orthologs without open chromatin tended to have lower ranks for our multi-species model predictions than they do for mean conservation scores, and we found that the ranks were significantly lower for our predictions (brain predictions vs. PhastCons scores: 4.34×10^{-5}, brain predictions vs. PhyloP scores: 8.08×10^{-9}, liver predictions vs. PhastCons scores: 7.15×10^{-14}, liver predictions vs. PhyloP scores: 9.82×10^{-22}) (**Supplemental Tables 7-8, 16**).

Relationships between Liver Clusters and Mouse Candidate Enhancers Associated with Liver Regeneration

We investigated whether each liver cluster that was active in mouse overlapped mouse candidate enhancers associated with liver regeneration [56] more than expected by chance (**Supplemental Table 19**). We found that candidate liver enhancers that have increased activity four weeks after hepatocyte repopulation relative to the control were enriched for overlapping a cluster with predicted Murinae-specific open chromatin – cluster 29 – as well as two clusters with predicted Muroidea-specific open chromatin – cluster 36 and cluster 100 (**Supplemental Figure 12c**). We think that these results are unlikely to be explained by the number of usable orthologs or conservation because OCRs overlapping this

candidate enhancer set do not have significantly fewer usable orthologs or lower conservation according to PhastCons [2] or PhyloP [1] than liver OCRs overall. We also found that candidate liver enhancers that have increased activity one week after hepatocyte repopulation relative to the control were enriched for overlapping a cluster with noisy predicted Primate-specific liver non-open chromatin (cluster 83), suggesting that their orthologs in a non-Murinae clade are closed. In contrast to these findings, liver candidate enhancers with decreased activity four weeks or one week after hepatocyte repopulation relative to the control were not enriched for overlapping any clusters, and liver candidate enhancers with decreased activity four weeks after hepatocyte repopulation relative to one week after hepatocyte repopulation were enriched for overlapping a cluster without a clear pattern of predicted open chromatin (cluster 39). These results suggest that there may be a relationship between liver regeneration in Murinae and Murinae-specific open chromatin, though we could not determine why the cluster enrichments differ for enhancers up-regulated at different numbers of weeks after regeneration.

Additional Limitations of Our Method

Despite the numerous advantages of predicting open chromatin status with short sequences, using shorter input sequences also has limitations. Some enhancers, such as super-enhancers, are much longer than 500 base pairs, and such enhancers have been shown to play important roles in the brain [57]. In addition, open chromatin status can be affected by long-range interactions with DNA sequences that are more than a few hundred base pairs away from open chromatin peak summits [58]. For example, one study showed that many variants associated with open chromatin occur at least a few hundred base pairs away from OCRs [59]. Encouragingly, our knowledge of 3D genome structure is advancing rapidly, so incorporating such information into machine learning models may be feasible soon. Furthermore, open chromatin status changes over evolutionary history can be affected by factors not influenced by local

sequence, such as changes in TFs' protein structures that affect their ability to interact with DNA or other TFs [60], so any model with only DNA sequence underlying OCRs as input will not be able to predict every OCR ortholog open chromatin status difference between species.

In addition, training and evaluating any machine learning model using regulatory genomics data from multiple species is inherently limited because raising different species in the same type of controlled environment is infeasible, and this can make differentiating between lineage-specific OCRs and confounding factor-specific OCRs difficult. For example, the activity of many enhancers has been associated with aging [61, 62]. Although all of our data were from adults, the mouse [30, 63] and rat data were from younger adults, whereas the human and macaque data came from a combination of younger and older adults [31, 64, 65]. Part of our motivation for conservatively defining clade-specific OCRs was the desire to prevent Glires-specific OCRs from being young adult-specific OCRs. In addition, time of day and the amount of time since waking up has been shown to affect enhancer activity [66], and controlling for these factors is challenging when obtaining post-mortem human data or data from different animal colonies. Although our macaque and rat samples were collected approximately two hours after the animals woke up, time of day of collection relative to sleep cycle for the remaining samples used was either not described or not able to be controlled [30, 31, 63-65]. Thus, some individual OCR ortholog open chromatin status differences between species and tissues could be affected by the amount of time that the animal had been awake, in addition to species and tissue differences. Furthermore, an animal's sex has been shown to be associated with the activity of both brain [67, 68] and liver [69] enhancers. Although all our datasets with multiple biological replicates had both males and females, the number of male and female replicates differed between datasets. We hope that our conservative definitions of clade-specific, species-specific, and tissue-specific OCRs prevented these OCRs from being sex-specific OCRs.

Furthermore, while our CNN provided accurate predictions of open chromatin conservation, using a CNN for our machine learning model has limitations. CNNs require inputs of a fixed size; this prevented

us from accounting for differences in peak length between OCRs and would make using CNNs in future work incorporating long-range interactions difficult. CNNs also require extensive hyper-parameter tuning, and their performance can be sensitive to the random seed used in initialization. It is possible that, with more extensive hyper-parameter tuning or a different random seed, we would have been able to train models with better performance for some of the training sets whose models had poor performance for our criteria or to obtain models trained on only mouse sequences with comparably good performance to the multi-species models. While multiple Bayesian optimization methods exist for automating much of the hyper-parameter tuning process [70-72], these methods often require extensive compute time that is not available to many researchers. SVMs do not have CNNs' input size limits, have only a few hyper-parameters to tune, and have been shown to work well on related tasks [73-75], but their prediction time can be slow because their kernels need to be computed for every DNA sequence, which could make using SVMs for predicting open chromatin conservation of hundreds of thousands of OCRs in each of hundreds of species intractable. In addition, CNNs continue to be less directly interpretable than methods with user-defined features that cannot account for complex combinatorial relationships between sequence patterns involved in open chromatin, even though many advances have been made to improve the interpretability of CNNs [3, 4, 76]. Interpreting models for open chromatin conservation prediction could reveal the mechanisms through which enhancer orthologs have lost activity over evolutionary history, such as losses in TF motifs and changes in DNA shape.

<u>Potential Extensions of Our Work</u>

There are many ways to extend our approach for open chromatin conservation prediction that have the potential to both improve our accuracy and expand the space in which we can make predictions. For example, training a model on a few species with open chromatin data using genome-wide negatives

to predict OCRs genome-wide in species without open chromatin data would require substantial additional training time but may improve accuracy and would enable us to predict open chromatin in regions whose orthologs are not open in any of the species for which we have data. While some machine learning models have been successfully trained to predict open chromatin genome-wide [77, 78], such models have not yet been applied to predicting open chromatin conservation across species. Likewise, training a model that includes TF protein sequences and, if available, TF expression, could enable models to learn when differences between species in TF sequence or expression might be associated with differences in open chromatin. In addition, modifying our models to predict continuous open chromatin signal across species would enable us to not only predict changes in the existence of OCRs but also in their strength. A previous study trained CNNs to predict continuous open chromatin signal across species [79], suggesting that accomplishing this task might be feasible, but such models' ability to accurately predict changes in open chromatin between species has yet to be systematically evaluated. In fact, any extension to our approach would need to be evaluated for its ability to predict lineage- and tissue-specific open chromatin (**Figure 1b**), and, given that some of our models trained with widely used negative sets such as dinucleotide-shuffled sequences did not meet all our evaluation criteria, direct application of some existing methods to predicting open chromatin conservation may not initially be successful. Finally, using reporter assays to experimentally validate our predictions for species without available open chromatin could substantially improve the reliability of our work, though assaying large numbers of enhancers in primary tissue is not feasible with current technology [80], and cell lines often poorly capture tissue-specific transcriptional regulatory programs [81].

Another exciting extension to our work would be investigating the open chromatin regions where our models perform poorly, as these may be examples with transcriptional regulatory codes that are not conserved across mammals. For example, there are many TFs, such as some Krüppel-associated-box cys2-his2 zinc finger TFs, whose genes are present in only Euarchonta [82-84], so these TFs may bind to

incorrectly predicted Euarchonta-specific enhancers. Likewise, there are TFs that are expressed at different levels or in different tissues in different species, which can cause enhancer activity differences directly or through differences in co-binding with other TFs that enable enhancer activity [84-86], so these TFs also may bind to incorrectly predicted clade- or species-specific enhancers. OCRs with incorrect predictions may also be bound by TFs that bind differently between adults of different ages, different parts of the circadian cycle, or different conditions in different labs, as we were not able to control for all these factors in data collection and curation; thus, this investigation may also help reveal important factors that should be controlled for in future enhancer activity experiments.

SUPPLEMENTAL MATERIALS AND METHODS

Assaying Open Chromatin in Mouse Liver

We performed ATAC-seq experiments on two 10-week-old heterozygous Pvalb-2A-Cre mice (B6.Cg-Pvalb$^{tm1.1(cre)Aibs}$/J; Jackson Stock No: 012358) [87], one male (Replicate 1 in **Supplemental Figure 8a**) and one female (Replicate 2 in **Supplemental Figure 8a**). We euthanized the mice by isoflurane and decapitation. We quickly dissected fresh liver tissue and extracted nuclei by 30 strokes of Dounce homogenization with the loose pestle (0.005 in. clearance) in 5mL of cold lysis buffer [88]. We filtered the nuclei suspensions through a 70µm cell strainer, pelleted them by centrifugation at 2,000 x g for 10 minutes, resuspended them in water, and filtered them a final time through a 40µm cell strainer. We stained sample aliquots with DAPI (Invitrogen #D1206) and quantified nuclei concentrations using a manual hemocytometer under a fluorescent microscope. We then input approximately 50,000 nuclei into a 50µL ATAC-seq tagmentation reaction as described in [88] and [89]. We amplified the resulting

libraries to 1/3 qPCR saturation, and fragment length distributions estimated by the Agilent TapeStation System showed high-quality ATAC-seq periodicity. We paired-end-sequenced the samples on the Illumina NovaSeq 6000 System through Novogene services. We obtained 165,337,124 reads from the male mouse and 225,752,264 reads from the female mouse.

Identifying Brain and Liver OCRs

We used open chromatin data from four species: *Homo sapiens* [64, 65, 90], *Macaca Mulatta* [31], *Mus musculus* [30, 63], and *Rattus norvegicus* [31]. For human brain OCRs, we used NeuN+ primary motor cortex (4 biological replicates), putamen (4 biological replicates), and nucleus accumbens (1 biological replicate) ATAC-seq data from GSE96949 [64] and caudate and putamen DNase hypersensitivity data (2 biological replicates) from ENCODE [65]. For macaque brain OCRs, we used orofacial motor cortex (2 biological replicates), hand motor cortex (2 biological replicates), caudate (2 biological replicates), putamen (2 biological replicates), and nucleus accumbens (1 biological replicate) ATAC-seq data from our previous study [31]. For macaque liver OCRs, we used liver ATAC-seq data (1 biological replicate) we previously generated [31]. For mouse brain OCRs, we used cortex and striatum ATAC-seq data from seven-week-old and twelve-week-old mice from our previous study [63] (2 biological replicates each). For mouse liver OCRs, we used the mouse liver ATAC-seq data that we generated as well as mouse liver ATAC-seq data from CNP0000198 [30] (4 biological replicates). For rat brain OCRs, we used primary motor cortex (3 biological replicates) and striatum data (2 biological replicates) from our previous study [31]. For rat liver OCRs, we used liver ATAC-seq data (2 biological replicates) from our previous study [31]. For each dataset, we combined reads from technical replicates. In addition, we identified Laurasiatheria-specific liver OCRs and non-OCRs using cow and pig liver ATAC-seq data (2 biological replicates each) from the FAANG Consortium [91].

We processed DNase hypersensitivity data by using the Kundaje Lab open chromatin pipeline [92] to map reads to hg38 [93], filter reads, call peaks, evaluate which peaks are reproducible, and remove peaks overlapping the ENCODE black list [94]. We used the default settings for the pipeline. We downloaded human brain DNase hypersensitivity data from the caudate nucleus and the putamen from the ENCODE portal [90, 95]. Since the caudate nucleus and the putamen are both parts of the striatum but came from different people, we treated them as biological replicates. The final set of peaks was the larger set of the peaks that were reproducible according to the Irreproducible Discovery Rate (IDR) [96] across biological replicates and the peaks that were reproducible according to the IDR across pooled pseudo-replicates (the "optimal set").

We processed the mouse brain ATAC-seq data using the Kundaje Lab open chromatin pipeline [92] and the mouse liver, human, macaque, and rat ATAC-seq data as well as the cow and pig ATAC-seq data we used for identifying Laurasiatheria-specific OCRs and non-OCRs using the ENCODE ATAC-seq pipeline [97]. For the mouse brain ATAC-seq data, we began with the filtered bam files from data we previously generated [63] and used the default parameters for the remainder of the pipeline. For the other ATAC-seq data, we used the default parameters except for "atac.multimapping" : 0, "atac.cap_num_peak" : 300000, "atac.smooth_win" : 150, "atac.enable_idr" : true, and "atac.idr_thresh" : 0.1; these parameter modifications enabled the parameters for read filtering, peak calling, and calculating the IDR to be the same as those used for the mouse brain data. We mapped the human data to hg38 [93], the macaque data to rheMac8 [98], the mouse data to mm10 [99], the rat data to rn6 [100], the cow data to NCBI assembly Btau_5.0.1 [101], and the pig data to susScr3 [102]. For the mouse liver ATAC-seq data from CNP0000198 [30], we treated the two female and two male samples as four biological replicates. The final set of peaks for datasets with multiple biological replicates was the larger set of the peaks that were reproducible according to the IDR [96] across biological replicates and the peaks that were reproducible according to IDR across pooled pseudo-replicates (the "optimal set"); the final set of

peaks for datasets with only 1 biological replicate was the peaks that were reproducible according to IDR across self-pseudo-replicates.

We then used the percentage of mapped reads, number of filtered reads, periodicity, TSS enrichment, number of IDR reproducible peaks, rescue ratio, and self-consistency ratio analyses generated by the pipelines [88, 103] to evaluate data quality. We found that most of the samples were high-quality. However, we excluded the second macaque nucleus accumbens biological replicate because it had only about sixteen million filtered reads and poor periodicity and because the two replicates had rescue ratio 4.01 and self-consistency ratio 2.04. We also excluded the second macaque liver replicate because it had only about two million filtered reads and poor periodicity and because the two replicates had self-consistency ratio 3.53. In addition, we excluded the first rat liver biological replicate it had only 35,593 reproducible peaks according to the IDR across self-pseudo-replicates despite having over sixty-eight million filtered reads. As a result, for macaque nucleus accumbens and liver, we used the peaks from the first biological replicate that were reproducible according to the IDR across self-pseudo-replicates, and, for rat liver, we used the "optimal set" from running the ENCODE ATAC-seq pipeline on only biological replicates 2 and 3.

Constructing Positive Sets

We gathered open chromatin data generated by ATAC-seq [88, 89] or DNase hypersensitivity [104] from two brain regions – cortex and striatum – in four species: *Homo sapiens* [64, 65, 90], *Macaca Mulatta* [31], *Mus musculus* [63], and *Rattus norvegicus* [31]. To obtain OCRs in each species, we intersected the IDR "optimal set" reproducible peaks from each of the brain regions and datasets for brain and each of the liver datasets for liver and defined OCRs to be the intersected peaks that are likely to be enhancers (**Supplemental Figure 13**). Specifically, for each species, we selected one set of reproducible

open chromatin peaks to be the "base peaks," used bedtools intersect with the -wa and -u options to intersect it with each of the other reproducible peak sets in series, and then used bedtools closestBed with the -t first and -d options to identify the "base peaks" that overlapped at least one peak from each other set that were over 20kb from the nearest protein-coding TSS (not promoters), at most 1kb long (not super-enhancers), and non-exonic [105]. The base peaks for human brain were the IDR "optimal set" from NeuN+ cells in the primary motor cortex from GSE96949 [64], for the macaque brain were the IDR "optimal set" from the orofacial motor cortex from data we previously generated [31], for the macaque liver were the IDR reproducible peaks across self-pseudo replicates from the first macaque liver replicate from data we previously generated [31], for mouse brain were the IDR "optimal set" from the cortex from the seven-week-old mouse from data we previously generated [63], for mouse liver were the IDR "optimal set" from our mouse liver ATAC-seq dataset, for the rat brain were the IDR "optimal set" from the primary motor cortex from data we previously generated [31], and for the rat liver were the IDR "optimal set" from the second and third rat liver replicates from data we previously generated [31]. To determine the distance from the nearest protein-coding TSS, we used the GENCODE protein-coding TSS's for human (version 27) and mouse (version M15) [106, 107], the union of the RefSeq rheMac8 protein-coding TSS's [108] and the human GENCODE protein-coding TSSs mapped to rheMac8 using liftOver [109] for macaque, the union of the RefSeq rn6 protein-coding TSSs [108] and the mouse GENCODE protein-coding TSSs mapped to rn6 using liftOver [109] for rat, the union of the RefSeq Btau_5.0.1 TSS's [108] and the human GENCODE protein-coding TSS's mapped to Btau_5.0.1 with halLiftover [110] on the version 1 Zoonomia Cactus alignment [111] for cow, and the union of the susScr11 TSS's mapped to susScr3 [98] with liftOver [109] and the human GENCODE protein-coding TSS's mapped to susScr3 with halLiftover [110] on the version 1 Zoonomia Cactus alignment [111] for pig. To identify non-exonic peaks, we used bedtools [105] subtract with option -A to identify peaks that did not overlap protein-coding exons, where human protein-coding exons were obtained from GENCODE (version 27), mouse protein-coding exons were obtained

from GENCODE (version M15) [106, 107], macaque protein-coding exons were obtained from RefSeq for rheMac8, and rat protein-coding exons were obtained from RefSeq for rn6 [108], cow protein-coding exons were obtained from RefSeq for Btau_5.0.1 [108], and pig protein-coding exons were obtained from RefSeq for susScr11 [108] and then mapped to susScr3 with liftOver [109]. We defined the peak summit of an OCR to be the peak summit of the corresponding base peak, and we constructed positive examples by taking likely enhancer peaks summits +/- 250bp and their reverse complements. For all species, tissue combinations except for macaque brain and rat liver, if there were multiple peaks with identical coordinates and different summits, we kept all peaks; for macaque brain and rat liver, we kept the first peak. We centered peaks on their summits because previous work has shown that there is a concentration of TF motifs at peak summits [112-114]. We then defined our brain OCRs to be the 250bp in each direction of summits of non-exonic cortex open chromatin peaks that (1) overlap striatum open chromatin peaks, (2) are less than 1kb (to exclude super-enhancers), and (3) are at least 20kb from the nearest transcription start site (TSS) so that so they would not overlap promoters. We defined liver OCRs in the same way except, rather than requiring overlap with striatum, we required overlap with any other liver OCR datasets we had processed from the same species. By requiring OCRs to be reproducible open chromatin peaks according to IDR, intersecting OCRs across multiple datasets, and filtering OCRs in a conservative way, we limited the number of false positive OCRs being used to train our machine learning models.

Constructing Additional Negative Training Sets

We could not directly compare the model trained on our novel negative set to existing models for predicting enhancers across species because the existing models were trained for different tasks. Some previous models were trained for 3kb [73] or variable-length [115] H3K27ac ChIP-seq regions instead of

500bp OCRs. Another model took > 100kb input sequences to predict open chromatin in many cell lines instead of 500bp input sequences to predict open chromatin in brain or liver primary tissue [79], and an additional model predicted open chromatin in melanoma cell lines [116], which have different transcriptional regulatory programs from brain and liver. Therefore, we instead created negative training sets based on the negative sets used in these studies and trained brain models using them to compare to the model trained using our novel negative set.

Flanking Regions

We constructed the flanking region negatives by using bedtools [105, 117] to identify the subset of regions flanking mouse brain OCRs that are not OCRs (**Supplemental Figure 1a**). Specifically, we first identified the 500bp flanking regions of each of our OCRs +/- 500bp; we required a 500bp separation between flanks and OCRs to ensure that our negatives would not include false negatives due to poorly defined peak boundaries. We then removed all flanking regions that overlapped any peaks from the mouse brain union pooled peaks (**Supplemental Figure 13**) [92, 97]; we used these peaks instead of the subset of such peaks that we defined as OCRs because non-reproducible peaks have the potential to be enhancers, and we wanted to limit the number of false negatives in our training set. For each remaining flanking region, we used its underlying sequence and that sequence's reverse complement. Thus, although there could be up to two negatives for every positive, our negative:positive training data ratio was approximately 1.65:1 (**Table 2**).

OCRs from Other Tissues

We constructed the OCRs from other tissues negatives by identifying OCRs in non-cortex and non-striatum tissues that do not overlap our brain OCRs (**Supplemental Figure 1a**). We first used the ENCODE ATAC-seq pipeline [97] with the same parameters that we used for the brain samples to process all of the ATAC-seq data from tissues that do not overlap cortex and striatum from the mouse ENCODE post-natal

samples [118] and from CNP0000198 [30]. We then used the same quality control metrics that we used for selecting datasets to include as OCRs to evaluate the quality of these datasets and removed those that were low-quality. The mouse ENCODE datasets that we included were from liver, intestine, and cerebellum. (We did not use this for our liver positive set because it came from an embryonic sample, and the other datasets were from adults.) The datasets from CNP0000198 [30] that we included were from female abdominal fat, female adrenal gland, female kidney, male kidney, female liver, male liver, female lung, male lung, female pancreas, male small intestine, male spleen, male stomach, female thymus, and male thymus. (For the purposes of creating these negatives, male and female samples were processed separately for all tissues, including liver.) We obtained the union of the IDR "optimal set" peaks across all of these datasets as well as our mouse liver data and used bedtools subtract with the -A option [105] to remove those peaks that overlapped open any mouse brain union pooled peaks (**Supplemental Figure 13**). For each filtered peak, we used the sequence underlying its summit +/- 250bp and that sequence's reverse complement. Our negative:positive training data ratio was approximately 19.78:1 (**Table 2**).

G/C- and Repeat-Matched Regions

We identified G/C- and repeat-matched regions for our OCRs using a combination of R packages and bedtools (**Supplemental Figure 1a**) [105]. We first created a repeat-masked mm10 genome by running forgeMaskedBSgenomeDataPkg from the BSgenome R package [119] on mm10 [99] with masks downloaded from the UCSC Genome Browser [120]. We then ran genNullSeqs from the gkmSVM R package [74, 121] on the sequences of the brain OCR peak summits +/- 250bp and our masked mouse genome with default parameters except for the following: length_match_tol=0.00, which ensures that all of our sequences are 500bp; nMaxTrials=100, which allows for more attempts to find G/C- and repeat-matched regions than the default; and xfold=10 for the larger G/C- and repeat-matched region negatives and =2 for the smaller G/C- and repeat-matched region negatives. Although we allowed for more trials,

getNullSeqs found fewer G/C- and repeat-matched regions than we had requested. After generating these regions, we used bedtools subtract [105] with the -A option to remove any regions that overlapped any open chromatin peaks called from the mouse brain union pooled peaks (**Supplemental Figure 13**). For each filtered G/C- and repeat-matched region, we used its underlying sequence and that sequence's reverse complement. As a result, for the larger G/C- and repeat-matched training set, the negative:positive training data ratio was approximately 8.15:1, and, for the smaller G/C- and repeat-matched training set, the negative:positive training data ratio was approximately 1.64:1 (**Table 2**).

Dinucleotide-Shuffled OCRs

We obtained dinucleotide shuffled OCRs by running the MEME suite's [122] fasta-shuffle-letters on the sequences of our brain OCR peak summits +/- 250bp (**Supplemental Figure 1a**). We used the default parameters except for -kmer 2, which enabled us to preserve dinucleotide frequencies, and -copies 10, which enabled us to generate ten times as many negatives as positives. We used every shuffled sequence and its reverse complement. Thus, the negative:positive training data ratio was exactly 10:1 (**Table 2**).

Constructing Training, Validation, and Test Sets

For the models trained using only mouse sequences, we divided the positives and negatives (except for the dinucleotide-shuffled OCRs) into training, validation, and test sets based on chromosomes to ensure that there would be no overlap between the sets. For the dinucleotide-shuffled OCRs negatives, we put each of them into the set that corresponded to the positive example from which it was constructed. Our training set chromosomes were mm10 chromosomes 3-7, 10-19, and X. Our validation set chromosomes that we used for developing our positive and negative set definitions [for example, validation set performance was used to determine that we should use orthologs of loose OCRs instead of

OCRs for our novel negative set (**Supplemental Figure 13**)], early stopping, and hyper-parameter tuning were mm10 chromosomes 8-9. Our test set chromosomes were mm10 chromosomes 1-2. We performed all presented evaluations on mouse genomic regions, including those on types of regions not used in model training, on only regions from mm10 chromosomes 1-2.

For the models and model evaluations using sequences from non-mouse species, we divided sequences into training, validation, and test sets based on the chromosomes to which their mouse orthologs mapped. In other words, we mapped such regions to mm10 using halLiftover [110] with the Zoonomia version 1 Cactus alignment [111] followed by HALPER [112] with parameters -max_frac 2.0, -min_len 50, and -protect_dist 5 and put them into the training set if their mm10 orthologs were on chromosomes 3-7, 10-19, or X; put them into the validation set if their mm10 orthologs were on chromosomes 8-9; put them into the test set if their mm10 orthologs were on chromosomes 1-2; and excluded them if their orthologs were elsewhere in mm10 or if they had no orthologs. Although many non-mouse regions were excluded from evaluation, because some OCRs have high sequence conservation, not accounting for the location of mouse orthologs when constructing training, validation, and test sets could lead to test set sequences that are almost identical to training set sequences [90, 123]. All evaluation set results that we present are from regions that map to the mouse test set chromosomes.

Training Machine Learning Models

We tuned hyper-parameters for the CNNs trained with training sets based on training sets from previous studies, the CNN for the liver data that used only mouse sequences, and the CNNs for the multi-species models by beginning with the architecture that we used for our mouse sequence-only brain models and our novel negative set. We then adjusted the number of convolutional filters per layer and

the learning rate, ultimately selecting the values that provided the best performance on the validation set. For the model with flanking region negatives, we used 250 convolutional filters per layer and learning rate 0.001. For the model with the OCRs from other tissue negatives, we used 250 convolutional filters per layer and learning rate 0.0005. For the model with the larger number of random G/C- and repeat-matched negatives, the multi-species brain model, and both liver models, we used 350 convolutional filters per layer and learning rate 0.001 (**Table 3**). For the models with the smaller number of random G/C- and repeat-matched negatives and the dinucleotide-shuffled negatives, we used 300 convolutional filters per layer and learning rate 0.001. All models were implemented and trained using Keras [124] version 1.2.2 with the Theano backend [125] and evaluated using Scikit-learn [126] and PRROC [127]. A complete list of our models is in **Table 2**.

Calibrating Machine Learning Models

Because the machine learning models trained with some of the training sets had high sensitivity and low specificity, we re-calibrated them with the training data from the training set whose negatives were our novel negative set. More specifically, we first made predictions with the model we wanted to re-calibrate on the training data from the positive set and our novel negative set. We next trained a logistic regression to use the model's predictions as features to predict the real open chromatin status for these training examples. We then used the logistic regression to make predictions on the relevant evaluation sets. We did the training and prediction using Scikit-learn (**Supplemental Figure 4, Supplemental Figure 5c, Supplemental Tables 1-6, Supplemental Tables 10-12**) [126].

Evaluating the Relationship between OCR Ortholog Open Chromatin Status and Genome Quality

To evaluate the relationship between predicted OCR ortholog open chromatin status and genome quality, we computed the correlation between the mean and standard deviation of predicted mouse test chromosome OCR ortholog open chromatin status and the Glires' genome assemblies' scaffold and contig N50's. We obtained the scaffold and contig N50's from NCBI [27, 44] and computed the log base ten of each of them. We computed the correlations for predictions from multi-species brain and liver models, using brain and liver OCR orthologs, respectively (**Supplemental Figure 11**). We also determined the relative association of phylogenetic distance and genome quality with predictions by fitting generalized linear models of mean and standard deviation of predicted mouse test chromosome OCR ortholog open chromatin status as a combination of divergence from mouse and scaffold or contig N50. In addition to comparing the effect sizes for the generalized linear models, we also computed the p-values on the coefficients and multiplied them by four to do a Bonferroni correction (**Supplemental Tables 14-15**).

<u>Interpreting Deep Learning Models</u>

We interpreted the deep learning models by computing the importance of every nucleotide in each true positive example in the validation set and then using these importance values to construct motifs. We computed the importance of every nucleotide in every true positive example in the validation set using DeepLIFT, which calculates the extent to which each input contributes to the prediction relative to a reference [3]. We used the DeepLIFT version 0.5.5-theano with the Rescale rule scores from the sequence layer with the target of the final convolutional layer, where our reference was a sequence of N's. We also used an extension to DeepLIFT, also with the Rescale rule, to compute the "hypothetical scores" for each nucleotide at each position for each sequence, which can be thought of as the preference of the model for observing each nucleotide at each position in the sequence [4].

We combined the scores and hypothetical scores using the TF-MoDISco method to construct "TF-MoDISco Motifs" [4]. TF-MoDISco first identifies frequently occurring sequence patterns with high DeepLIFT scores within the sequences of each OCR (called seqlets), next computes a similarity matrix between all seqlets, and then uses the similarity matrix to cluster the seqlets into nonredundant motifs. We used the following settings for TF-MoDIsco: seqlet FDR threshold = 0.2; gapped k-mer settings for similarity computation k-mer length = 8, number of gaps = 1, and number of mismatches = 0; and final motif width = 50. We visualized our TF-MoDISco motifs from TF-MoDISco using the aggregated hypothetical scores of the seqlets supporting each motif. We created position frequency matrices from TF-MoDISco motifs by averaging the one-hot-encoded sequences at all of the seqlet coordinates belonging to the motifs and compared them to known motifs by running TomTom [128] on them with the *Mus musculus* motifs from CIS-BP (**Supplemental Figure 6, Supplemental Figure 8, and Supplemental Figure 10**) [129].

Comparing Liver Open Chromatin Predictions to H3K27ac ChIP-seq:

We compared our liver open chromatin predictions to liver H3K27ac ChIP-seq regions from a multi-species dataset [43]. So that we would have sufficient power, for this evaluation, we included all OCRs and their orthologs instead of limiting to OCRs with mouse orthologs on test chromosomes. We first used halLiftover [110] with the Zoonomia version 1 Cactus alignment [111] followed by HALPER [112] with settings -max_frac 2.0, -min_len 50, and -protect_dist 5 to identify orthologs of all mouse, rat, and macaque liver OCRs in all Zoonomia species except for *Manis tricuspis*, which was not in the Cactus alignment. We next used our multi-species liver model to predict the liver open chromatin statuses of the orthologs and orthologs' reverse complements in all the species except for *Galeopterus variegatus*, *Hippopotamus amphibius*, *Monodon monoceros*, *Platanista gangetica*, and *Procavia capensis*, which we

excluded due to challenges converting between chromosome naming conventions. Then, for each OCR ortholog, we set the prediction to be the average between the prediction for the ortholog and the prediction for its reverse complement. After that, we obtained the liver H3K27ac ChIP-seq regions in each species and those regions' orthologs in other species with liver H3K27ac ChIP-seq data as well as whether those orthologs overlapped H3K27ac ChIP-seq regions from https://www.ebi.ac.uk/research/flicek/publications/FOG15 [43]. We mapped the rat ChIP-seq regions from rn5 to rn6 and the macaque ChIP-seq regions from rheMac2 to rheMac8 using liftOver [109]. We finally filtered the liver OCRs by removing those that did not overlap H3K27ac ChIP-seq regions in the same species.

When evaluating the relationship between liver open chromatin predictions and liver H3K27ac ChIP-seq conservation, we considered all Boreoeutheria with liver H3K27ac ChIP-seq except for *Chlorocebus sabaeus* because the H3K27ac ChIP-seq reads were mapped to *Chlorocebus pygerythrus* instead of *Chlorocebus sabaeus* [43]. For each of mouse, rat, and macaque, we considered H3K27ac ChIP-seq to be conserved if there was a liver H3K27ac ChIP-seq region overlapping the ortholog and to be non-conserved if the ortholog did not have an overlapping H3K27ac ChIP-seq region; we did not include any species for which either the H3K27ac ChIP-seq data or our overlapping OCRs had no ortholog. Then, for each combination of species for which we had liver open chromatin data and species with liver H3K27ac ChIP-seq data, we compared the multi-species liver model predictions for the orthologs with conserved H3K27ac ChIP-seq to those for the orthologs with non-conserved H3K27ac ChIP-seq using a Wilcoxon rank-sum test; we did a Bonferroni correction by multiplying all p-values by twenty-nine, which was the number of tests we did. We also found that median of our predictions for the orthologs with conserved H3K27ac ChIP-seq was higher than the median of our predictions for the orthologs with non-conserved H3K27ac ChIP-seq (**Supplemental Table 13**).

Obtaining and Visualizing Signal Tracks

We obtained the signal tracks used in **Figure 4** using the pooled replicates fold-change bigwigs from the data processing pipelines. For the H3K27ac ChIP-seq data, we downloaded the mouse and macaque H3K27ac ChIP-seq data from E-MTAB-2633 [43] and reprocessed it using the AQUAS Transcription Factor and Histone ChIP-Seq processing pipeline [130] with default parameters, mapping reads to mm10 and rheMac8, respectively. We evaluated the data quality of each biological replicate based on the percentage of mapped reads, number of filtered reads, NSC, RSC, number of IDR reproducible peaks, rescue ratio, and self-consistency ratio analyses generated by the pipelines and found that all four biological replicates from each species were high-quality. We created visualizations for these figures using the New WashU Epigenome Browser [131].

SUPPLEMENTAL FIGURE CAPTIONS

Supplemental Figure 1: Additional Lineage-Specific OCR Accuracy Evaluations for Models Trained with Different Training Sets

a) Illustration of negatives for different training sets.

b) Performance of models trained with all mouse sequence-only brain training sets on corresponding test sets.

c) Test chromosome performance of models trained on all mouse sequence-only brain training sets on MouseBr≠OtherBr.

d) Test chromosome performance of models trained on all mouse sequence-only brain training sets on MouseBr≠RatBr.

e) Test chromosome performance of models trained on all mouse sequence-only brain training sets on GliresBr≠EuarchontaBr.

f) Test chromosome performance of models trained on all mouse sequence-only brain training sets on MacaqueBr≠MouseBr.

g) Test chromosome performance of models trained on all mouse sequence-only brain training sets on HumanBr≠MouseBr.

h) Test chromosome performance of models trained on all mouse sequence-only brain training sets on RatBr≠MouseBr.

i) Test chromosome performance of models trained on all mouse sequence-only brain training sets on EuarchontaBr≠GliresBr.

Animal silhouettes were obtained from PhyloPic [132]. AUC stands for area under the receiver operating characteristic curve, AUPRC stands for area under the precision-recall curve, Rep. stands for repeat, Dinuc.-Shuf. stands for dinucleotide-shuffled, and Orths. stands for orthologs. For evaluations with more positives than negatives, we reported the area under the negative predictive value (NPV)-specificity (Spec.) curve instead of the AUPRC.

Supplemental Figure 2: Performance of Brain Model Trained with Smaller G/C- and Repeat-Matched Negatives before and after Calibration

a) Test chromosome performance on GliresBr≠EuarchontaBr before and after calibration with training set positives and non-OCR orthologs of OCR negatives. We reported the negative predictive value (NPV) instead of the precision because there are more positives than negatives in this evaluation.

b) Test chromosome performance on EuarchontaBr≠GliresBr before and after calibration with training set positives and non-OCR orthologs of OCRs.

Animal silhouettes were obtained from PhyloPic [132].

Supplemental Figure 3: Violin Plots for Lineage-Specific and Tissue-Specific OCR Accuracy Evaluation in Human

Comparison of PhastCons [2] and PhyloP [1] scores to three different machine learning models' predictions for brain OCRs with conserved open chromatin across mouse and human, human brain OCRs whose mouse orthologs are closed in brain, human brain non-OCRs whose mouse orthologs are open in brain, human brain OCRs that are closed in liver, human brain OCRs that are open in liver (centered on brain peak summits), and human liver OCRs that are closed in brain. +'s indicate that values should be large, and -'s indicate that values should be small. Conservation scores were generated from the mm10-based placental mammals alignment [133, 134] and averaged over 500bp centered on peak summits, where mouse peak summits were used for OCRs conserved between mouse and human and for OCRs in mouse whose human orthologs are closed, and mouse orthologs of human peak summits were used for other evaluations. All machine learning model predictions were made using human sequences, where the human sequences for OCRs conserved between mouse and human and for OCRs in mouse with human orthologs that are not OCRs were centered on human orthologs of mouse peak summits, and human peak summits were used for other evaluations. Note that the models in the third and fourth panels were trained on only mouse sequences, demonstrating their performance in a species not used in training. Animal silhouettes were obtained from PhyloPic [132]. *'s indicate the species from which sequences were obtained for making predictions. Dinuc.-shuf. stands for dinucleotide-shuffled, and Orths. stands for orthologs.

Supplemental Figure 4: Violin Plots for Lineage-Specific and Tissue-Specific OCR Accuracy Evaluation in Rat

a) Comparison of PhastCons [2] and PhyloP [1] scores to three different machine learning models' predictions for brain OCRs with conserved open chromatin across mouse and rat, rat brain OCRs whose mouse orthologs are closed in brain, rat brain non-OCRs whose mouse orthologs are open in brain, rat brain OCRs that are closed in liver, rat brain OCRs that are open in liver (centered on brain peak summits), and rat liver OCRs that are closed in brain.

b) Comparison of PhastCons [2] and PhyloP [1] scores to two different machine learning models' predictions for liver OCRs with conserved open chromatin across mouse and rat, rat liver OCRs whose mouse orthologs are closed in liver, rat liver non-OCRs whose mouse orthologs are open in liver, rat liver OCRs that are closed in brain, rat liver OCRs that are open in brain (centered on liver peak summits), and rat brain OCRs that are closed in liver.

+'s indicate that values should be large, and -'s indicate that values should be small. Conservation scores were generated from the mm10-based placental mammals alignment [133, 134] and averaged over 500bp centered on peak summits, where mouse peak summits were used for OCRs conserved between mouse and rat and for OCRs in mouse whose rat orthologs are closed, and mouse orthologs of rat peak summits were used for other evaluations. All machine learning model predictions were made using rat sequences, where the rat sequences for OCRs conserved between mouse and rat and for OCRs in mouse whose rat orthologs are closed were centered on rat orthologs of mouse peak summits, and rat peak summits were used for other evaluations. Note that the models in the third and fourth panels were trained on only mouse sequences, demonstrating their performance in a species not used in training. Animal silhouettes were obtained from PhyloPic [132]. *'s indicate the species from which sequences were obtained for making predictions. Dinuc.-shuf. stands for dinucleotide-shuffled, and Orths. stands for orthologs.

Supplemental Figure 5: Additional Tissue-Specific OCR Accuracy Evaluations – Performance of Brain Models on Liver OCRs

a) We made test chromosome predictions with machine learning models trained on different mouse sequence-only brain training sets on brain open chromatin regions (OCRs) that do not overlap liver OCRs, brain OCRs that overlap liver OCRs, liver OCRs that do not overlap brain OCRs, and negatives from test sets associated with training sets. p-Values were computed with a Wilcoxon rank-sum test, and we did a Bonferroni correction across all mouse sequence-only brain training sets.

b) We evaluated the test chromosome performance of the mouse sequence-only brain models on MouseBrVsLv (snowflakes), HumanBrVsLv (dots), and RatBrVsLv (x's).

c) We evaluated the test chromosome performance of the brain model trained with the smaller G/C- and repeat-matched negatives on MouseBrVsLv (snowflakes), HumanBrVsLv (dots), and RatBrVsLv (x's) before and after calibration with the training set positives and non-OCR orthologs of OCRs.

The moues silhouette was obtained from PhyloPic [132]. AUC stands for area under the receiver operating characteristic curve, AUPRC stands for area under the precision-recall curve, Rep. stands for repeat, and Orths. stands for orthologs.

Supplemental Figure 6: TF-MoDISco Motifs from Brain Models Trained with Different Training Sets

Each table contains motifs from TF-MoDISco; transcription factors (TFs) whose motifs match the TF-MoDISco motifs with TomTom q-value < 0.05 ordered from most to least significant TomTom p-value [128], where red TFs are those whose motifs are considered important and gold TFs are those whose motifs' depletions are considered important; and number of supporting seqlets for each motif.

a) TF-MoDISco motifs for brain model from the training set with flanking region negatives.

b) TF-MoDISco motifs for brain model from the training set with open chromatin regions (OCRs) from other tissue negatives.

c) TF-MoDISco motifs for brain model from the training set with larger G/C- and repeat-matched region negatives.

d) TF-MoDISco motifs for brain model from the training set with smaller G/C- and repeat-matched region negatives.

e) TF-MoDISco motifs for brain model from the training set with dinucleotide-shuffled OCR negatives.

f) TF-MoDISco motifs for brain model from the training set with non-OCR orthologs of OCRs negatives.

Supplemental Figure 7: Phylogeny-Matching Correlations Evaluation from Brain Models Trained with Different Training Sets

a) Divergence from mouse versus mean of the test chromosome brain open chromatin region (OCR) ortholog open chromatin status predictions across each Glires species from the model trained on each training set. The curves are the best fit exponential functions of the form $y = ae^{bx}$. The dotted lines are the average predictions across test set negatives.

b) Divergence from mouse versus standard deviation (Std. Dev.) of the test chromosome brain OCR ortholog open chromatin status predictions across each of the Glires from the model trained on each training set. The curves are the best fit exponential functions of the form $y = c/(1 + ae^{-bx})$.

Animal silhouettes were obtained from PhyloPic [132]. Rep. stands for repeat, Orths. stands for orthologs, and MYA stands for millions of years ago.

Supplemental Figure 8: Performance of Mouse Liver Models

a) Periodicity plots for each of the biological replicates from our new mouse liver ATAC-seq data.

b) Test set chromosome performance of mouse liver models on MouseLv, MouseLv≠OtherLv, MouseLv≠RatLv, MacaqueLv≠MouseLv, RatLv≠MouseLv, GliresLv≠EuarchontaLv, and EuarchontaLv≠GliresLv. For MouseLv, MacaqueLv≠MouseLv, and EuarchontaLv≠GliresLv, we reported the

area under the negative predictive value (NPV)-specificity (Spec.) curve because these evaluations had more positives than negatives.

c) Test set chromosome performance of mouse liver models on MouseLvVsBr (snowflakes), MacaqueLvVsBr (dots), and RatLvVsBr (x's).

d) Divergence from mouse versus mean predictions across mouse test chromosome liver OCR orthologs in Glires. The curve is the best fit exponential function of the form $y = ae^{bx}$. The dotted line is the average prediction across test set negatives. MYA stands for millions of years ago.

e) Divergence from mouse versus standard deviation (Std. Dev.) of predictions across mouse test chromosome liver OCR orthologs in Glires. The curve is the best fit exponential function of the form $y = c/(1 + ae^{-bx})$. MYA stands for millions of years ago.

f) TF-MoDISco motifs for the mouse liver model; transcription factors (TFs) whose motifs match the TF-MoDISco motifs with TomTom q-value < 0.05 ordered from most to least significant TomTom p-value [128], where red TFs are those whose motifs are considered important and gold TFs are those whose motifs' depletions are considered important; and number of supporting seqlets for each motif.

Animal silhouettes were obtained from PhyloPic [132]. AUC stands for area under the receiver operating characteristic curve, and AUPRC stands for area under the precision-recall curve.

Supplemental Figure 9: Violin Plots for Liver Model Lineage-Specific and Tissue-Specific OCR Accuracy Evaluation in Macaque

Comparison of PhastCons [2] and PhyloP [1] scores to two different machine learning models' predictions for liver OCRs with conserved open chromatin across mouse and macaque, macaque liver OCRs whose mouse orthologs are closed in liver, macaque liver non-OCRs whose mouse orthologs are open in liver, macaque liver OCRs that are closed in brain, macaque liver OCRs that are open in brain (centered on liver peak summits), and macaque brain OCRs that are closed in liver. +'s indicate that values should be large,

and -‘s indicate that values should be small. Conservation scores were generated from the mm10-based placental mammals alignment [133, 134] and averaged over 500bp centered on peak summits, where mouse peak summits were used for OCRs conserved between mouse and macaque and for OCRs in mouse whose macaque orthologs are closed, and mouse orthologs of macaque peak summits were used for other evaluations. All machine learning model predictions were made using macaque sequences, where the macaque sequences for OCRs conserved between mouse and macaque and for OCRs in mouse whose macaque orthologs are closed were centered on macaque orthologs of mouse peak summits, and macaque peak summits were used for other evaluations. Note that the model in the third panel was trained on only mouse sequences, demonstrating its performance in a species not used in training. Animal silhouettes were obtained from PhyloPic [132]. *’s indicate the species from which sequences were obtained for making predictions. Orths. stands for orthologs.

Supplemental Figure 10: Additional Evaluations from Multi-Species Brain and Liver Models

a) Divergence from mouse versus standard deviation (Std. Dev.) of multi-species brain model predictions across mouse test chromosome brain open chromatin region (OCR) orthologs in Glires. The red curve is the best fit exponential function of the form $y = c/(1 + ae^{-bx})$. MYA stands for millions of years ago.

b) Divergence from mouse versus Std. Dev. of multi-species liver model predictions across mouse test chromosome liver OCR orthologs in Glires. The red curve is the best fit exponential function of the form $y = c/(1 + ae^{-bx})$. MYA stands for millions of years ago.

c) TF-MoDISco motifs for multi-species brain model; transcription factors (TFs) whose motifs match the TF-MoDISco motifs with TomTom q-value < 0.05 ordered from most to least significant TomTom p-value [128], where red TFs are those whose motifs are considered important and gold TFs are those whose motifs’ depletions are considered important; and number of supporting seqlets for each motif.

d) TF-MoDISco motifs for multi-species brain model; TFs whose motifs match the TF-MoDISco motifs with TomTom q-value < 0.05 ordered from most to least significant TomTom p-value [128], where red TFs are those whose motifs are considered important and gold TFs are those whose motifs' depletions are considered important; and number of supporting seqlets for each motif.

Animal silhouettes were obtained from PhyloPic [132].

Supplemental Figure 11: Genome Quality versus Open Chromatin Status Predictions in Glires

a) log base ten of scaffold and contig N50's of each of the Glires versus mean test chromosome brain open chromatin region (OCR) ortholog open chromatin status prediction across each of the Glires.

b) log base ten of scaffold and contig N50's of each of the Glires versus mean test chromosome liver OCR ortholog open chromatin status prediction across each of the Glires.

c) log base ten of scaffold and contig N50's of each of the Glires versus standard deviation (Std. Dev.) of test chromosome brain OCR ortholog open chromatin status predictions across each of the Glires.

d) log base ten of scaffold and contig N50's of each of the Glires versus Std. Dev. of test chromosome liver OCR ortholog open chromatin status predictions across each of the Glires.

Supplemental Figure 12: Additional Predicted Lineage-Specific OCR Clusters Associated with Neuron Firing, Neuron Activity, and Liver Regeneration

a) Additional predicted Murinae-specific brain open chromatin region (OCR) cluster (cluster 27) with significant overlap with mouse enhancers associated with neuron firing.

b) Predicted Hystricognathi-specific brain non-OCR cluster (cluster 11) and Muroidea and Pecora-specific non-OCR cluster (cluster 48) with significant overlap with human enhancers associated with neuron activity.

c) We clustered the liver OCRs where the features were the liver predictions in each Boreoeutherian species from Zoonomia and then identified clusters whose regions had significant overlap with regions associated with mouse liver regeneration. These clusters were a Murinae-specific OCR cluster (cluster 29, top) and two Muroidea-specific OCR clusters (cluster 36, middle, and cluster 100, bottom). Animal silhouettes were obtained from PhyloPic [132].

Supplemental Figure 13: Illustrations of Definitions of “OCRs” that Are Reproducible Peaks across All Datasets for a Tissue, Species Combination; “Loose OCRs;” and “Union Pooled Peaks”

SUPPLEMENTAL TABLES

Supplemental Table 1: Mouse Sequence Brain Model Sensitivity on Glires-Specific Test Chromosome Brain OCRs and Non-OCRs before and after Calibration

Negatives in Training Set	Uncalibrated Model Sensitivity	Calibrated Model Sensitivity
Flanking Regions	0.84	0.74
OCRs in Other Tissues	0.73	0.71
Large G/C- and Repeat-Matched	0.71	0.70
Small G/C- and Repeat-Matched	0.86	0.72
Dinucleotide-Shuffled OCRs	0.83	0.67
Non-OCR Orths. of OCRs	0.74	0.77

Supplemental Table 2: Mouse Sequence Brain Model Specificity on Glires-Specific Test Chromosome Brain OCRs and Non-OCRs before and after Calibration

Negatives in Training Set	Uncalibrated Model Specificity	Calibrated Model Specificity
Flanking Regions	0.81	0.90
OCRs in Other Tissues	0.79	0.82
Large G/C- and Repeat-Matched	0.95	0.96
Small G/C- and Repeat-Matched	0.74	0.88
Dinucleotide-Shuffled OCRs	0.61	0.79
Non-OCR Orths. of OCRs	0.92	0.92

Supplemental Table 3: Mouse Sequence Brain Model Precision on Glires-Specific Test Chromosome Brain OCRs and Non-OCRs before and after Calibration

Negatives in Training Set	Uncalibrated Model Precision	Calibrated Model Precision

Flanking Regions	0.89	0.92
OCRs in Other Tissues	0.86	0.87
Large G/C- and Repeat-Matched	0.96	0.97
Small G/C- and Repeat-Matched	0.85	0.91
Dinucleotide-Shuffled OCRs	0.79	0.85
Non-OCR Orths. of OCRs	0.94	0.94

Supplemental Table 4: Mouse Sequence Brain Model Sensitivity on Euarchonta-Specific Brain OCRs and Non-OCRs before and after Calibration, where OCR and Non-OCRs' Mouse Orthologs Are on Test Chromosomes

Negatives in Training Set	Uncalibrated Model Sensitivity	Calibrated Model Sensitivity
Flanking Regions	0.74	0.65
OCRs in Other Tissues	0.75	0.72
Large G/C- and Repeat-Matched	0.60	0.57
Small G/C- and Repeat-Matched	0.84	0.69
Dinucleotide-Shuffled OCRs	0.68	0.51
Non-OCR Orths. of OCRs	0.60	0.61

Supplemental Table 5: Mouse Sequence Brain Model Specificity on Euarchonta-Specific Brain OCRs and Non-OCRs before and after Calibration, where OCR and Non-OCRs' Mouse Orthologs Are on Test Chromosomes

Negatives in Training Set	Uncalibrated Model Specificity	Calibrated Model Specificity
Flanking Regions	0.64	0.80
OCRs in Other Tissues	0.66	0.68
Large G/C- and Repeat-Matched	0.84	0.86
Small G/C- and Repeat-Matched	0.55	0.73
Dinucleotide-Shuffled OCRs	0.54	0.72
Non-OCR Orths. of OCRs	0.85	0.83

Supplemental Table 6: Mouse Sequence Brain Model Precision on Euarchonta-Specific Brain OCRs and Non-OCRs before and after Calibration, where OCR and Non-OCRs' Mouse Orthologs Are on Test Chromosomes

Negatives in Training Set	Uncalibrated Model Precision	Calibrated Model Precision
Flanking Regions	0.55	0.65
OCRs in Other Tissues	0.56	0.57
Large G/C- and Repeat-Matched	0.69	0.70
Small G/C- and Repeat-Matched	0.52	0.60
Dinucleotide-Shuffled OCRs	0.46	0.51
Non-OCR Orths. of OCRs	0.70	0.68

Supplemental Table 7: PhastCons, PhyloP, and Predictions for Mouse Test Chromosome Brain OCRs with Conserved versus Non-Conserved Open Chromatin Status

Species with Mouse OCR Orthologs	PhastCons Scores	PhyloP Scores	Predictions from Brain Model Trained on Mouse	Predictions from Multi-Species Brain Model
Macaque	4.82×10^{-18}	4.12×10^{-10}	8.06×10^{-129}	2.39×10^{-143}
Human	6.13×10^{-9}	1.05×10^{-6}	4.19×10^{-68}	4.59×10^{-80}
Rat	1.50×10^{-16}	7.18×10^{-14}	6.73×10^{-123}	7.14×10^{-149}

Supplemental Table 8: PhastCons, PhyloP, and Predictions for Mouse Test Chromosome Liver OCRs with Conserved versus Non-Conserved Open Chromatin Status

Species with Mouse OCR Orthologs	PhastCons Scores	PhyloP Scores	Predictions from Liver Model Trained on Mouse	Predictions from Multi-Species Liver Model
Macaque	1.69×10^{-5}	> 1	1.34×10^{-182}	1.75×10^{-228}
Rat	1.30×10^{-4}	> 1	4.46×10^{-157}	4.19×10^{-213}

Supplemental Table 9: OCR Predictions by Mouse Sequence Models on Other Species' Orthologs versus Conservation Scores

Tissue	Species	Conservation Score Type	Open Chromatin Conserved	Open Chromatin Not Conserved
Brain	Human	PhastCons	5.81×10^{-4}	4.26×10^{-1}
Brain	Human	PhyloP	7.09×10^{-5}	9.77×10^{-2}
Brain	Rat	PhastCons	1.24×10^{-2}	4.80×10^{-3}
Brain	Rat	PhyloP	9.50×10^{-3}	3.60×10^{-8}
Liver	Rat	PhastCons	5.85×10^{-6}	9.30×10^{-2}
Liver	Rat	PhyloP	6.98×10^{-7}	2.54×10^{-2}

Supplemental Table 10: Mouse Sequence Brain Model Sensitivity on Liver OCRs before and after Calibration

Negatives in Training Set	Uncalibrated Model Sensitivity	Calibrated Model Sensitivity
Flanking Regions	0.90	0.85
OCRs in Other Tissues	0.71	0.69
Large G/C- and Repeat-Matched	0.80	0.79
Small G/C- and Repeat-Matched	0.91	0.83
Dinucleotide-Shuffled OCRs	0.86	0.79
Non-OCR Orths. of OCRs	0.85	0.86

Supplemental Table 11: Mouse Sequence Brain Model Specificity on Liver OCRs before and after Calibration

Negatives in Training Set	Uncalibrated Model Specificity	Calibrated Model Specificity
Flanking Regions	0.79	0.89
OCRs in Other Tissues	0.97	0.98
Large G/C- and Repeat-Matched	0.95	0.95
Small G/C- and Repeat-Matched	0.75	0.89
Dinucleotide-Shuffled OCRs	0.53	0.69

Non-OCR Orths. of OCRs	0.93	0.92

Supplemental Table 12: Mouse Sequence Brain Model Precision on Liver OCRs before and after Calibration

Negatives in Training Set	Uncalibrated Model Precision	Calibrated Model Precision
Flanking Regions	0.56	0.70
OCRs in Other Tissues	0.88	0.90
Large G/C- and Repeat-Matched	0.82	0.83
Small G/C- and Repeat-Matched	0.53	0.70
Dinucleotide-Shuffled OCRs	0.36	0.44
Non-OCR Orths. of OCRs	0.78	0.77

Supplemental Table 13: Wilcoxon Rank-Sum Test p-Values for Differences in Predicted (Multi-Species Model) Open Chromatin in Liver Open Chromatin + H3K27ac ChIP-seq Region Orthologs with and without H3K27ac ChIP-seq (All Directions Match Expectations)

Species with H3K27ac	Mouse OCRs	Rat OCRs	Macaque OCRs
Mus musculus	N/A	9.45 x 10^{-101}	4.04 x 10^{-78}
Callithrix jacchus	1.51 x 10^{-46}	6.61 x 10^{-50}	3.03 x 10^{-106}
Macaca mulatta	8.86 x 10^{-58}	8.05 x 10^{-55}	N/A
Felis catus	1.30 x 10^{-39}	9.36 x 10^{-32}	4.39 x 10^{-87}
Homo sapiens	1.05 x 10^{-50}	8.40 x 10^{-47}	3.32 x 10^{-71}
Bos taurus	2.00 x 10^{-49}	3.42 x 10^{-51}	1.73 x 10^{-94}
Canis lupus familiaris	9.10 x 10^{-39}	5.76 x 10^{-33}	3.10 x 10^{-105}
Oryctolagus cuniculus	2.42 x 10^{-29}	3.59 x 10^{-38}	9.75 x 10^{-59}
Heterocephalus glaber	2.09 x 10^{-11}	N/A	N/A
Cavia porcellus	8.06 x 10^{-14}	N/A	N/A
Sus scrofa	3.13 x 10^{-34}	8.57 x 10^{-27}	8.15 x 10^{-64}
Rattus norvegicus	1.74 x 10^{-104}	N/A	6.82 x 10^{-63}

Supplemental Table 14: Test Chromosome GLM Results – Mean Prediction as Function of Divergence from Mouse and log10(Scaffold/Contig N50)

Tissue	Genome Quality Metric	Distance from Mouse Coefficient	Distance from Mouse p-Value	Genome Quality Coefficient	Genome Quality p-Value
Brain	Scaffold N50	-2.78 x 10^{-3}	2.80 x 10^{-22}	1.88 x 10^{-10}	5.88 x 10^{-1}
Brain	Contig N50	-2.82 x 10^{-3}	6.09 x 10^{-26}	7.39 x 10^{-10}	1.74 x 10^{-1}
Liver	Scaffold N50	-2.22 x 10^{-3}	4.89 x 10^{-8}	3.25 x 10^{-10}	8.44 x 10^{-1}
Liver	Contig N50	-2.35 x 10^{-3}	1.92 x 10^{-9}	1.77 x 10^{-9}	1.04

Supplemental Table 15: Test Chromosome GLM Results – Standard Deviation of Prediction as Function of Divergence from Mouse and log10(Scaffold/Contig N50)

Tissue	Genome Quality Metric	Distance from Mouse Coefficient	Distance from Mouse p-Value	Genome Quality Coefficient	Genome Quality p-Value
Brain	Scaffold N50	4.99×10^{-4}	1.03×10^{-14}	-1.60×10^{-10}	1.30×10^{-4}
Brain	Contig N50	5.63×10^{-4}	9.87×10^{-18}	-8.31×10^{-10}	1.08×10^{-3}
Liver	Scaffold N50	4.79×10^{-4}	3.98×10^{-4}	-1.47×10^{-10}	2.53×10^{-1}
Liver	Contig N50	5.27×10^{-4}	4.55×10^{-7}	-8.11×10^{-10}	3.22×10^{-1}

Supplemental Table 16: Test Chromosome OCR Predictions by Multi-Species Models on Other Species' Orthologs versus Conservation Scores

Tissue	Species	Conservation Score Type	Brain Open Chromatin Conserved	Brain Open Chromatin Not Conserved
Brain	Human	PhastCons	5.85×10^{-6}	9.30×10^{-2}
Brain	Human	PhyloP	6.98×10^{-7}	2.54×10^{-2}
Brain	Rat	PhastCons	7.05×10^{-4}	1.62×10^{-9}
Brain	Rat	PhyloP	5.95×10^{-4}	3.99×10^{-12}
Liver	Rat	PhastCons	5.00×10^{-22}	1.52×10^{-11}
Liver	Rat	PhyloP	1.88×10^{-25}	1.65×10^{-17}

Supplemental Table 17: Significance of Overlap between Mouse Neuron Firing Enhancers (Bic Induces Neuron Firing, TTX Blocks Neuron Firing) and Brain Clusters Active in Mouse

Cluster	Bic-Specific Enhancers	Activity-Invariant Enhancers	TTX-Specific Enhancers
cluster 1	> 1	8.54×10^{-19}	> 1
cluster 13	> 1	> 1	> 1
cluster 17	> 1	> 1	> 1
cluster 23	> 1	5.27×10^{-1}	> 1
cluster 26	> 1	9.47×10^{-1}	> 1
cluster 27	6.00×10^{-3}	> 1	> 1
cluster 30	7.28×10^{-1}	> 1	> 1
cluster 37	> 1	6.97×10^{-3}	> 1
cluster 4	> 1	> 1	> 1
cluster 43	2.37×10^{-3}	> 1	> 1
cluster 49	> 1	> 1	> 1
cluster 51	> 1	> 1	> 1
cluster 58	> 1	> 1	> 1
cluster 60	> 1	9.10×10^{-1}	> 1
cluster 63	> 1	> 1	> 1
cluster 71	> 1	> 1	> 1
cluster 79	3.47×10^{-1}	6.60×10^{-2}	> 1
cluster 81	> 1	1.92×10^{-8}	> 1
cluster 82	> 1	5.94×10^{-2}	> 1
cluster 88	> 1	3.83×10^{-2}	> 1
cluster 94	> 1	> 1	> 1

Supplemental Table 18: Significance of Overlap between Human Neuron Activity Up and Down Enhancers (Minutes/Hours: Time after KCl Exposure) and Brain Clusters Active in Human

Cluster	↑, 15 Minutes	↓, 15 Minutes	↑, 2 Hours	↓, 2 Hours
cluster 1	5.04×10^{-2}	4.83×10^{-3}	> 1	2.97×10^{-3}
cluster 11	> 1	> 1	6.18×10^{-4}	> 1
cluster 13	> 1	> 1	> 1	> 1
cluster 21	> 1	> 1	> 1	> 1
cluster 26	> 1	> 1	> 1	> 1
cluster 29	> 1	> 1	> 1	> 1
cluster 32	> 1	> 1	> 1	> 1
cluster 41	> 1	> 1	> 1	> 1
cluster 42	> 1	> 1	> 1	> 1
cluster 48	> 1	> 1	5.84×10^{-3}	> 1
cluster 5	> 1	> 1	> 1	> 1
cluster 55	> 1	9.70×10^{-1}	> 1	> 1
cluster 61	> 1	> 1	> 1	> 1
cluster 67	> 1	> 1	> 1	> 1
cluster 73	> 1	> 1	> 1	> 1
cluster 74	> 1	> 1	4.52×10^{-2}	> 1
cluster 77	> 1	> 1	> 1	> 1
cluster 78	> 1	> 1	> 1	> 1
cluster 81	> 1	> 1	> 1	> 1
cluster 82	> 1	> 1	> 1	2.03×10^{-2}
cluster 95	> 1	> 1	> 1	> 1
cluster 96	> 1	> 1	> 1	> 1
cluster 99	> 1	> 1	> 1	> 1

Supplemental Table 19: Significance of Overlap between Mouse Liver Regeneration Enhancers (Wk.: Weeks into Hepatocyte Repopulation) and Liver Clusters Active in Mouse

Cluster	Wk. 1 ↑ vs. Ctl.	Wk. 1 ↓ vs. Ctl.	Wk. 4 ↑ vs. Ctl.	Wk. 4 ↓ vs. Ctl.	Wk. 4 ↑ vs. Wk. 1	Wk. 4 ↓ vs. Wk. 1
cluster 100	> 1	> 1	6.36×10^{-4}	> 1	> 1	> 1
cluster 17	> 1	> 1	> 1	> 1	> 1	> 1
cluster 18	> 1	> 1	> 1	> 1	> 1	> 1
cluster 28	7.60×10^{-1}	> 1	> 1	> 1	> 1	> 1
cluster 29	> 1	> 1	2.00×10^{-3}	> 1	> 1	> 1
cluster 2	6.96×10^{-1}	> 1	2.43×10^{-1}	> 1	> 1	> 1
cluster 31	> 1	> 1	> 1	> 1	> 1	> 1
cluster 34	> 1	> 1	> 1	> 1	> 1	> 1
cluster 36	> 1	> 1	9.29×10^{-3}	> 1	> 1	> 1
cluster 39	3.31×10^{-1}	> 1	> 1	> 1	> 1	6.60×10^{-3}
cluster 51	> 1	> 1	> 1	> 1	> 1	> 1

cluster 55	> 1	> 1	> 1	> 1	> 1	> 1
cluster 59	> 1	> 1	> 1	> 1	> 1	> 1
cluster 64	> 1	> 1	> 1	> 1	> 1	> 1
cluster 69	> 1	> 1	> 1	> 1	> 1	> 1
cluster 75	> 1	> 1	> 1	> 1	> 1	> 1
cluster 76	> 1	> 1	> 1	> 1	> 1	> 1
cluster 78	> 1	> 1	> 1	> 1	> 1	> 1
cluster 83	4.34×10^{-2}	> 1	> 1	> 1	> 1	> 1
cluster 84	> 1	> 1	> 1	> 1	> 1	> 1
cluster 8	> 1	> 1	> 1	> 1	> 1	> 1
cluster 9	> 1	> 1	> 1	> 1	> 1	> 1
cluster 93	> 1	> 1	> 1	> 1	> 1	> 1
cluster 94	> 1	> 1	> 1	> 1	> 1	> 1

Supplemental Table 20: Evaluation Set Names and Genomes

Evaluation Set Name	Genomes in Evaluation	Positives in Evaluation	Negatives in Evaluation
MouseBrFlank	mm10	Mouse Brain OCRs	Flanking Regions of Brain OCRs
MouseBrOtherTis	mm10	Mouse Brain OCRs	OCRs in Non-Brain Tissues
MouseBrLGC	mm10	Mouse Brain OCRs	Large G/C- and Repeat-Matched Regions of Brain OCRs
MouseBrSGC	mm10	Mouse Brain OCRs	Small G/C- and Repeat-Matched Regions of Brain OCRs
MouseBrShuf	mm10	Mouse Brain OCRs	Dinucleotide-Shuffled Brain OCRs
MouseBr	mm10	Mouse Brain OCRs	Mouse Non-Brain-OCR Orths. of Loose non-Mouse Brain OCRs
MouseBr≠OtherBr	mm10	Mouse Brain OCRs whose Orth. in at Least 1 Other Species Is Not a Brain OCR	Mouse Non-Brain-OCR Orths. of non-Mouse Brain OCRs
MouseBr≠RatBr	mm10	Mouse Brain OCRs whose Rat Orth. Is Not a Brain OCR	Mouse Non-Brain-OCR Orths. of Rat Brain OCRs
GliresBr≠EuarchontaBr	mm10	Glires-Specific Brain OCRs	Glires-Specific Brain non-OCRs
MacaqueBr≠MouseBr	rheMac8	Macaque Brain OCRs whose Mouse Orth. Is Not a Brain OCR	Macaque Non-Brain-OCR Orths. of Mouse Brain OCRs

HumanBr≠MouseBr	hg38	Human Brain OCRs whose Mouse Orth. Is Not a Brain OCR	Human Non-Brain-OCR Orths. of Mouse Brain OCRs
RatBr≠MouseBr	rn6	Rat Brain OCRs whose Mouse Orth. Is Not a Brain OCR	Rat Non-Brain-OCR Orths. of Mouse Brain OCRs
EuarchontaBr≠GliresBr	hg38	Euarchonta-Specific Brain OCRs	Euarchonta-Specific Brain Non-OCRs
MouseBrVsLv	mm10	Mouse Brain and Liver OCRs	Mouse Liver, Non-Brain OCRs
MacaqueBrVsLv	rheMac8	Macaque Brain and Liver OCRs	Macaque Liver, Non-Brain OCRs
RatBrVsLv	rn6	Rat Brain and Liver OCRs	Rat Liver, Non-Brain OCRs
MouseLv	mm10	Mouse Liver OCRs	Mouse Non-Liver-OCR Orths. of Loose Non-Mouse Liver OCRs
MouseLv≠OtherLv	mm10	Mouse Liver OCRs whose Orth. In at Least 1 Other Species Is Not a Liver OCR	Mouse Non-Liver-OCR Orths. of Non-Mouse Liver OCRs
MouseLv≠RatLv	mm10	Mouse Liver OCRs whose Rat Orth. Is Not a Liver OCR	Mouse Non-Liver-OCR Orths. of Rat Liver OCRs
MacaqueLv≠MouseLv	rheMac8	Macaque Liver OCRs whose Mouse Orth. Is Not a Liver OCR	Macaque Non-Liver-OCR Orths. of Mouse Liver OCRs
RatLv≠MouseLv	rn6	Rat Liver OCRs whose Mouse Orth. in Not a Brain OCRs	Rat Non-Liver-OCR Orths. of Mouse Liver OCRs
GliresLv≠EuarchontaLv	mm10	Glires-Specific Liver OCRs	Glires-Specific Liver Non-OCRs
EuarchontaLv≠GliresLv	rheMac8	Euarchonta-Specific Liver OCRs	Euarchonta-Specific Liver Non-OCRs
MouseLvVsBr	mm10	Mouse Liver and Brain OCRs	Mouse Brain, Non-Liver OCRs
MacaqueLvVsBr	rheMac8	Macaque Liver and Brain OCRs	Macaque Brain, Non-Liver OCRs
RatLvVsBr	rn6	Rat Liver and Brain OCRs	Rat Brain, Non-Liver OCRs
MultiBr	mm10, hg38, rheMac8, rn6	Brain OCRs	Non-Brain-OCR Orths. of Loose Brain OCRs
MultiBrClade	mm10, hg38	Clade-Specific Brain OCRs	Clade-Specific Brain non-OCRs
MultiBrSpecies	mm10, hg38, rheMac8, rn6	Species-Specific Brain OCRs	Species-Specific Brain Non-OCRs

MultiBrVsLv	mm10, rheMac8, rn6	Brain and Liver OCRs	Liver, Non-Brain OCRs
MultiLv	mm10, rheMac8, rn6	Liver OCRs	Non-Liver-OCR Orths. of Loose Liver OCRs
MultiLvClade	mm10, rheMac8	Clade-Specific Liver OCRs (non-Laurasiatheria)	Clade-Specific Liver non-OCRs (non-Laurasiatheria)
MultiLvSpecies	mm10, rn6	Species-Specific Liver OCRs	Species-Specific Liver non-OCRs
MultiLvVsBr	mm10, rheMac8, rn6	Liver and Brain OCRs	Brain, Non-Liver OCRs
MultiLvLauras	Btau_5.0.1	Laurasiatheria-Specific Liver OCRs	Laurasiatheria-Specific Liver Non-OCRs

Supplemental Table 21: Number of Positives and Negatives Used in Each Evaluation (not necessarily used in training models)

Evaluation Set Name	Number of Positives in Evaluation	Number of Negatives in Evaluation	Negatives:Positives in Evaluation
MouseBrFlank	4576	7440	1.63:1
MouseBrOtherTis	4576	82172	17.96:1
MouseBrLGC	4576	32008	6.99:1
MouseBrSGC	4576	6654	1.45:1
MouseBrShuf	4576	45760	10:1
MouseBr	4576	4694	1.03:1
MouseBr≠OtherBr	1570	2062	1.31:1
MouseBr≠RatBr	674	990	1.32:1
GliresBr≠EuarchontaBr	230	134	1:1.72
MacaqueBr≠MouseBr	734	788	1.07:1
HumanBr≠MouseBr	416	896	2.15:1
RatBr≠MouseBr	990	676	1:1.46
EuarchontaBr≠GliresBr	134	230	1.72:1
MouseBrVsLv	1040	3382	3.25:1
MacaqueBrVsLv	846	1898	2.24:1
RatBrVsLv	1770	3518	1.99:1
MouseLv	7752	4434	1:1.75
MouseLv≠OtherLv	3738	4080	1.09:1
MouseLv≠RatLv	2482	2050	1:1.21
MacaqueLv≠MouseLv	2384	2228	1:1.07
RatLv≠MouseLv	2050	2482	1.21:1
GliresLv≠EuarchontaLv	1024	1826	1.78:1
EuarchontaLv≠GliresLv	1826	1024	1:1.78
MouseLvVsBr	2012	2212	1.10:1
MacaqueLvVsBr	946	1428	1.51:1
RatLvVsBr	1130	2942	2.60:1
MultiBr	15266	19688	1.29:1
MultiBrClade	364	364	1:1

MultiBrSpecies	1918	1920	1:1
MultiBrVsLv	3656	8798	2.41:1
MultiLv	17688	14544	1:1.22
MultiLvClade	2850	2850	1:1
MultiLvSpecies	4532	4532	1:1
MultiLvVsBr	4088	6582	1.61:1
MultiLvLauras	154	172	1.12:1

Supplemental Table 22: Figures (S Means Supplemental Figure) with Results from Each Model (Column Headers Are Model Numbers from Table 2), Evaluation (Row Headers Are Evaluation Set Names from Supplemental Table 20) Combinations for Evaluations Other than Models' Test Sets

Evaluation Set Name	1	2	3	4	5	6	7	8	9
MouseBrFlank	S1b	N/A	N/A	N/A	N/A	N/A	N/A	N/A	N/A
MouseBrOtherTis	N/A	S1b	N/A	N/A	N/A	N/A	N/A	N/A	N/A
MouseBrLGC	N/A	N/A	S1b	N/A	N/A	N/A	N/A	N/A	N/A
MouseBrSGC	N/A	N/A	N/A	S1b	N/A	N/A	N/A	N/A	N/A
MouseBrShuf	N/A	N/A	N/A	N/A	S1b	N/A	N/A	N/A	N/A
MouseBr	N/A	N/A	N/A	N/A	N/A	S1b	N/A	N/A	N/A
MouseBr≠OtherBr	S1c	S1c	S1c	S1c	S1c	S1c	N/A	N/A	N/A
MouseBr≠RatBr	S1d	S1d	S1d	S1d	S1d	S1d	N/A	N/A	N/A
GliresBr≠EuarchontaBr	S1e	S1e	S1e	S1e	S1e	S1e	N/A	N/A	N/A
MacaqueBr≠MouseBr	S1f	S1f	S1f	S1f	S1f	S1f	N/A	N/A	N/A
HumanBr≠MouseBr	S1g	S1g	S1g	S1g	S1g	S1g	N/A	N/A	N/A
RatBr≠MouseBr	S1h	S1h	S1h	S1h	S1h	S1h	N/A	N/A	N/A
EuarchontaBr≠GliresBr	S1i	S1i	S1i	S1i	S1i	S1i	N/A	N/A	N/A
MouseBrVsLv	S5b	S5b	S5b	S5b	S5b	S5b	N/A	N/A	N/A
MacaqueBrVsLv	S5b	S5b	S5b	S5b	S5b	S5b	N/A	N/A	N/A
RatBrVsLv	S5b	S5b	S5b	S5b	S5b	S5b	N/A	N/A	N/A
MouseLv	N/A	N/A	N/A	N/A	N/A	N/A	S8b	N/A	N/A
MouseLv≠OtherLv	N/A	N/A	N/A	N/A	N/A	N/A	S8b	N/A	N/A
MouseLv≠RatLv	N/A	N/A	N/A	N/A	N/A	N/A	S8b	N/A	N/A
MacaqueLv≠MouseLv	N/A	N/A	N/A	N/A	N/A	N/A	S8b	N/A	N/A
RatLv≠MouseLv	N/A	N/A	N/A	N/A	N/A	N/A	S8b	N/A	N/A
GliresLv≠EuarchontaLv	N/A	N/A	N/A	N/A	N/A	N/A	S8b	N/A	N/A
EuarchontaLv≠GliresLv	N/A	N/A	N/A	N/A	N/A	N/A	S8b	N/A	N/A
MouseLvVsBr	N/A	N/A	N/A	N/A	N/A	N/A	S8c	N/A	N/A
MacaqueLvVsBr	N/A	N/A	N/A	N/A	N/A	N/A	S8c	N/A	N/A
RatLvVsBr	N/A	N/A	N/A	N/A	N/A	N/A	S8c	N/A	N/A
MultiBr	N/A	N/A	N/A	N/A	N/A	N/A	N/A	3a	N/A
MultiBrClade	N/A	N/A	N/A	N/A	N/A	N/A	N/A	3a	N/A
MultiBrSpecies	N/A	N/A	N/A	N/A	N/A	N/A	N/A	3a	N/A
MultiBrVsLv	N/A	N/A	N/A	N/A	N/A	N/A	N/A	3a	N/A
MultiLv	N/A	N/A	N/A	N/A	N/A	N/A	N/A	N/A	3b
MultiLvClade	N/A	N/A	N/A	N/A	N/A	N/A	N/A	N/A	3b

MultiLvSpecies	N/A	N/A	N/A	N/A	N/A	N/A	N/A	N/A	3b
MultiLvVsBr	N/A	N/A	N/A	N/A	N/A	N/A	N/A	N/A	3b
MultiLvLauras	N/A	N/A	N/A	N/A	N/A	N/A	3e	N/A	3e

SUPPLEMENTAL REFERENCES

1. Pollard KS, Hubisz MJ, Rosenbloom KR, Siepel A: **Detection of nonneutral substitution rates on mammalian phylogenies.** *Genome Research* 2010, **20**:110-121.
2. Siepel A, Bejerano G, Pedersen JS, Hinrichs AS, Hou M, Rosenbloom K, Clawson H, Spieth J, Hillier LDW, Richards S *et al*: **Evolutionarily conserved elements in vertebrate, insect, worm, and yeast genomes**. *Genome Research* 2005, **15**:1034-1050.
3. Shrikumar A, Greenside P, Kundaje A: **Learning Important Features Through Propagating Activation Differences**. *Proceedings of the 34th International Conference on Machine Learning* 2017, **70**:3145--3153.
4. Shrikumar A, Tian K, Shcherbina A, Avsec Ž, Banerjee A, Sharmin M, Nair S, Kundaje A: **TF-MoDISco v0.4.2.2-alpha: Technical Note**. https://arxiv.org/abs/1811.00416v1 (2018). Accessed May 2019.
5. Wendt KS, Yoshida K, Itoh T, Bando M, Koch B, Schirghuber E, Tsutsumi S, Nagae G, Ishihara K, Mishiro T *et al*: **Cohesin mediates transcriptional insulation by CCCTC-binding factor**. *Nature* 2008, **451**(7180):796-801.
6. Isbel L, Prokopuk L, Wu H, Daxinger L, Oey H, Spurling A, Lawther AJ, Hale MW, Whitelaw E: **Wiz binds active promoters and CTCF-binding sites and is required for normal behaviour in the mouse**. *Elife* 2016, **5**.
7. Herrera DG, Robertson HA: **Activation of c-fos in the brain**. *Prog Neurobiol* 1996, **50**(2-3):83-107.
8. Berretta S, Parthasarathy HB, Graybiel AM: **Local release of GABAergic inhibition in the motor cortex induces immediate-early gene expression in indirect pathway neurons of the striatum**. *J Neurosci* 1997, **17**(12):4752-4763.
9. Joo JY, Schaukowitch K, Farbiak L, Kilaru G, Kim TK: **Stimulus-specific combinatorial functionality of neuronal c-fos enhancers**. *Nat Neurosci* 2016, **19**(1):75-83.
10. Yamada K, Gerber DJ, Iwayama Y, Ohnishi T, Ohba H, Toyota T, Aruga J, Minabe Y, Tonegawa S, Yoshikawa T: **Genetic analysis of the calcineurin pathway identifies members of the EGR gene family, specifically EGR3, as potential susceptibility candidates in schizophrenia**. *Proc Natl Acad Sci U S A* 2007, **104**(8):2815-2820.
11. Swanberg SE, Nagarajan RP, Peddada S, Yasui DH, LaSalle JM: **Reciprocal co-regulation of EGR2 and MECP2 is disrupted in Rett syndrome and autism**. *Hum Mol Genet* 2009, **18**(3):525-534.
12. Kawase S, Kuwako K, Imai T, Renault-Mihara F, Yaguchi K, Itohara S, Okano H: **Regulatory factor X transcription factors control Musashi1 transcription in mouse neural stem/progenitor cells**. *Stem Cells Dev* 2014, **23**(18):2250-2261.
13. Zhang D, Zeldin DC, Blackshear PJ: **Regulatory factor X4 variant 3: a transcription factor involved in brain development and disease**. *J Neurosci Res* 2007, **85**(16):3515-3522.
14. Xu P, Morrison JP, Foley JF, Stumpo DJ, Ward T, Zeldin DC, Blackshear PJ: **Conditional ablation of the RFX4 isoform 1 transcription factor: Allele dosage effects on brain phenotype**. *PLoS One* 2018, **13**(1):e0190561.

15. Chen YC, Kuo HY, Bornschein U, Takahashi H, Chen SY, Lu KM, Yang HY, Chen GM, Lin JR, Lee YH *et al*: **Foxp2 controls synaptic wiring of corticostriatal circuits and vocal communication by opposing Mef2c**. *Nat Neurosci* 2016, **19**(11):1513-1522.
16. Harrington AJ, Raissi A, Rajkovich K, Berto S, Kumar J, Molinaro G, Raduazzo J, Guo Y, Loerwald K, Konopka G *et al*: **MEF2C regulates cortical inhibitory and excitatory synapses and behaviors relevant to neurodevelopmental disorders**. *Elife* 2016, **5**:e20059.
17. Mitchell AC, Javidfar B, Pothula V, Ibi D, Shen EY, Peter CJ, Bicks LK, Fehr T, Jiang Y, Brennand KJ *et al*: **MEF2C transcription factor is associated with the genetic and epigenetic risk architecture of schizophrenia and improves cognition in mice**. *Mol Psychiatry* 2018, **23**(1):123-132.
18. **The Human Protein Atlas**. 2005. https://www.proteinatlas.org/. Accessed August 2020.
19. Uhlén M, Fagerberg L, Hallstrom BM, Lindskog C, Oksvold P, Mardinoglu A, Sivertsson A, Kampf C, Sjostedt E, Asplund A *et al*: **Tissue-based map of the human proteome**. *Science* 2015, **347**:1260419-1260419.
20. Smith CM, Hayamizu TF, Finger JH, Bello SM, McCright IJ, Xu J, Baldarelli RM, Beal JS, Campbell J, Corbani LE *et al*: **The mouse Gene Expression Database (GXD): 2019 update**. *Nucleic Acids Res* 2019, **47**(D1):D774-D779.
21. Schwindt H, Akasaka T, Zühlke-Jenisch R, Hans V, Schaller C, Klapper W, Dyer MJ, Siebert R, Deckert M: **Chromosomal translocations fusing the BCL6 gene to different partner loci are recurrent in primary central nervous system lymphoma and may be associated with aberrant somatic hypermutation or defective class switch recombination**. *J Neuropathol Exp Neurol* 2006, **65**(8):776-782.
22. Tiberi L, Bonnefont J, van den Ameele J, Le Bon SD, Herpoel A, Bilheu A, Baron BW, Vanderhaeghen P: **A BCL6/BCOR/SIRT1 complex triggers neurogenesis and suppresses medulloblastoma by repressing Sonic Hedgehog signaling**. *Cancer Cell* 2014, **26**(6):797-812.
23. Yan L, Miyake S, Okamura H: **Distribution and circadian expression of dbp in SCN and extra-SCN areas in the mouse brain**. *J Neurosci Res* 2000, **59**(2):291-295.
24. Gachon F, Fonjallaz P, Damiola F, Gos P, Kodama T, Zakany J, Duboule D, Petit B, Tafti M, Schibler U: **The loss of circadian PAR bZip transcription factors results in epilepsy**. *Genes Dev* 2004, **18**(12):1397-1412.
25. González-Velasco O, Papy-García D, Le Douaron G, Sánchez-Santos JM, De Las Rivas J: **Transcriptomic landscape, gene signatures and regulatory profile of aging in the human brain**. *Biochim Biophys Acta Gene Regul Mech* 2020, **1863**(6):194491.
26. Armstrong J, Hickey G, Diekhans M, Fiddes IT, Novak AM, Deran A, Fang Q, Xie D, Feng S, Stiller J *et al*: **Progressive Cactus is a multiple-genome aligner for the thousand-genome era**. *Nature* 2020, **587**(7833):246-251.
27. Zoonomia Consortium: **A comparative genomics multitool for scientific discovery and conservation**. *Nature* 2020, **587**(7833):240-245.
28. Partha R, Chauhan BK, Ferreira Z, Robinson JD, Lathrop K, Nischal KK, Chikina M, Clark NL: **Subterranean mammals show convergent regression in ocular genes and enhancers, along with adaptation to tunneling**. *Elife* 2017, **6**:e25884.
29. Roller M, Stamper E, Villar D, Izuogu O, Martin F, Redmond AM, Ramachanderan R, Harewood L, Odom DT, Flicek P: **LINE retrotransposons characterize mammalian tissue-specific and evolutionarily dynamic regulatory regions**. *Genome Biol* 2021, **22**(1):62.
30. Liu C, Wang M, Wei X, Wu L, Xu J, Dai X, Xia J, Cheng M, Yuan Y, Zhang P *et al*: **An ATAC-seq atlas of chromatin accessibility in mouse tissues**. *Sci Data* 2019, **6**(1):65.
31. Wirthlin M, Kaplow, Irene M., Lawler, Alyssa J., He, J., Phan., BaDoi, N., Brown, Ashley R., Stauffer, William R., Pfenning, Andreas R.: **The Regulatory Evolution of the Primate Fine-Motor**

System. https://www.biorxiv.org/content/10.1101/2020.10.27.356733v1 (2020). Accessed October 2020.
32. Babeu JP, Boudreau F: **Hepatocyte nuclear factor 4-alpha involvement in liver and intestinal inflammatory networks**. *World J Gastroenterol* 2014, **20**(1):22-30.
33. Hoffman BG, Robertson G, Zavaglia B, Beach M, Cullum R, Lee S, Soukhatcheva G, Li L, Wederell ED, Thiessen N *et al*: **Locus co-occupancy, nucleosome positioning, and H3K4me1 regulate the functionality of FOXA2-, HNF4A-, and PDX1-bound loci in islets and liver**. *Genome Research* 2010, **20**(8):1037-51.
34. Alpern D, Langer D, Ballester B, Le Gras S, Romier C, Mengus G, Davidson I: **TAF4, a subunit of transcription factor II D, directs promoter occupancy of nuclear receptor HNF4A during post-natal hepatocyte differentiation**. *Elife* 2014, **3**:e03613.
35. Vietri Rudan M, Barrington C, Henderson S, Ernst C, Odom DT, Tanay A, Hadjur S: **Comparative Hi-C reveals that CTCF underlies evolution of chromosomal domain architecture**. *Cell Rep* 2015, **10**(8):1297-1309.
36. Schrem H, Klempnauer J, Borlak J: **Liver-enriched transcription factors in liver function and development. Part II: the C/EBPs and D site-binding protein in cell cycle control, carcinogenesis, circadian gene regulation, liver regeneration, apoptosis, and liver-specific gene regulation**. *Pharmacol Rev* 2004, **56**(2):291-330.
37. Hatzis P, Kyrmizi I, Talianidis I: **Mitogen-activated protein kinase-mediated disruption of enhancer-promoter communication inhibits hepatocyte nuclear factor 4alpha expression**. *Mol Cell Biol* 2006, **26**(19):7017-7029.
38. Plumb-Rudewiez N, Clotman F, Strick-Marchand H, Pierreux CE, Weiss MC, Rousseau GG, Lemaigre FP: **Transcription factor HNF-6/OC-1 inhibits the stimulation of the HNF-3alpha/Foxa1 gene by TGF-beta in mouse liver**. *Hepatology* 2004, **40**(6):1266-1274.
39. Margagliotti S, Clotman F, Pierreux CE, Beaudry JB, Jacquemin P, Rousseau GG, Lemaigre FP: **The Onecut transcription factors HNF-6/OC-1 and OC-2 regulate early liver expansion by controlling hepatoblast migration**. *Dev Biol* 2007, **311**(2):579-589.
40. Wang K: **Molecular mechanisms of hepatic apoptosis regulated by nuclear factors**. *Cell Signal* 2015, **27**(4):729-738.
41. Friedman JR, Kaestner KH: **The Foxa family of transcription factors in development and metabolism**. *Cell Mol Life Sci* 2006, **63**(19-20):2317-2328.
42. Lee CS, Friedman JR, Fulmer JT, Kaestner KH: **The initiation of liver development is dependent on Foxa transcription factors**. *Nature* 2005, **435**(7044):944-947.
43. Villar D, Berthelot C, Aldridge S, Rayner TF, Lukk M, Pignatelli M, Park TJ, Deaville R, Erichsen JT, Jasinska AJ *et al*: **Enhancer evolution across 20 mammalian species**. *Cell* 2015, **160**(3):554-566.
44. **National Center for Biotechnology Information (NCBI) Assembly [Internet]**. National Library of Medicine (US), National Center for Biotechnology Information, Bethesda, MD. 1988. https://www.ncbi.nlm.nih.gov/assembly. Accessed June 2019.
45. Song D, Chu Z, Min L, Zhen T, Li P, Han L, Bu S, yang J, Gonzale FJ, Liu A: **Gemfibrozil not fenofibrate decreases systemic glucose level via PPARα**. *Pharmazie* 2016, **71**(4):205-212.
46. Yang XN, Liu XM, Fang JH, Zhu X, Yang XW, Xiao XR, Huang JF, Gonzalez FJ, Li F: **PPARα Mediates the Hepatoprotective Effects of Nutmeg**. *J Proteome Res* 2018, **17**(5):1887-1897.
47. Kersten S, Stienstra R: **The role and regulation of the peroxisome proliferator activated receptor alpha in human liver**. *Biochimie* 2017, **136**:75-84.
48. Liu X, Xu J, Rosenthal S, Zhang LJ, McCubbin R, Meshgin N, Shang L, Koyama Y, Ma HY, Sharma S *et al*: **Identification of Lineage-Specific Transcription Factors That Prevent Activation of Hepatic Stellate Cells and Promote Fibrosis Resolution**. *Gastroenterology* 2020, **158**(6):1728-1744.e1714.

49. Park JS, Qiao L, Gilfor D, Yang MY, Hylemon PB, Benz C, Darlington G, Firestone G, Fisher PB, Dent P: **A role for both Ets and C/EBP transcription factors and mRNA stabilization in the MAPK-dependent increase in p21 (Cip-1/WAF1/mda6) protein levels in primary hepatocytes**. *Mol Biol Cell* 2000, **11**(9):2915-2932.
50. Sugawara H, Iwata H, Souri M, Ichinose A: **Regulation of human protein Z gene expression by liver-enriched transcription factor HNF-4alpha and ubiquitous factor Sp1**. *J Thromb Haemost* 2007, **5**(11):2250-2258.
51. Kilbourne EJ, Widom R, Harnish DC, Malik S, Karathanasis SK: **Involvement of early growth response factor Egr-1 in apolipoprotein AI gene transcription**. *J Biol Chem* 1995, **270**(12):7004-7010.
52. LaPensee CR, Lin G, Dent AL, Schwartz J: **Deficiency of the transcriptional repressor B cell lymphoma 6 (Bcl6) is accompanied by dysregulated lipid metabolism**. *PLoS One* 2014, **9**(6):e97090.
53. Sommars MA, Ramachandran K, Senagolage MD, Futtner CR, Germain DM, Allred AL, Omura Y, Bederman IR, Barish GD: **Dynamic repression by BCL6 controls the genome-wide liver response to fasting and steatosis**. *Elife* 2019, **8**.
54. Tang W, Jiang YF, Ponnusamy M, Diallo M: **Role of Nrf2 in chronic liver disease**. *World J Gastroenterol* 2014, **20**(36):13079-13087.
55. Xu D, Xu M, Jeong S, Qian Y, Wu H, Xia Q, Kong X: **The Role of Nrf2 in Liver Disease: Novel Molecular Mechanisms and Therapeutic Approaches**. *Front Pharmacol* 2018, **9**:1428.
56. Wang AW, Wang YJ, Zahm AM, Morgan AR, Wangensteen KJ, Kaestner KH: **The Dynamic Chromatin Architecture of the Regenerating Liver**. *Cell Mol Gastroenterol Hepatol* 2020, **9**(1):121-143.
57. Achour M, Le Gras S, Keime C, Parmentier F, Lejeune FX, Boutillier AL, Neri C, Davidson I, Merienne K: **Neuronal identity genes regulated by super-enhancers are preferentially down-regulated in the striatum of Huntington's disease mice**. *Hum Mol Genet* 2015, **24**(12):3481-3496.
58. Long HK, Prescott SL, Wysocka J: **Ever-Changing Landscapes: Transcriptional Enhancers in Development and Evolution**. *Cell* 2016, **167**(5):1170-1187.
59. Degner JF, Pai AA, Pique-Regi R, Veyrieras J-B, Gaffney DJ, Pickrell JK, De Leon S, Michelini K, Lewellen N, Crawford GE *et al*: **DNase I sensitivity QTLs are a major determinant of human expression variation**. In: *Nature.* vol. 482; 2012: 390-394.
60. Khoueiry P, Girardot C, Ciglar L, Peng PC, Gustafson EH, Sinha S, Furlong EE: **Uncoupling evolutionary changes in DNA sequence, transcription factor occupancy and enhancer activity**. *Elife* 2017, **6**:e28440.
61. Feser J, Tyler J: **Chromatin structure as a mediator of aging**. *FEBS Lett* 2011, **585**(13):2041-2048.
62. Bryois J, Garrett ME, Song L, Safi A, Giusti-Rodriguez P, Johnson GD, Shieh AW, Buil A, Fullard JF, Roussos P *et al*: **Evaluation of chromatin accessibility in prefrontal cortex of individuals with schizophrenia**. *Nat Commun* 2018, **9**(1):3121.
63. Srinivasan C, Phan BN, Lawler AJ, Ramamurthy E, Kleyman M, Brown AR, Kaplow IM, Wirthlin ME, Pfenning AR: **Addiction-associated genetic variants implicate brain cell type- and region-specific cis-regulatory elements in addiction neurobiology**. *J Neurosci* 2021, **41**(43):9008-9030.
64. Fullard JF, Hauberg ME, Bendl J, Egervari G, Cirnaru MD, Reach SM, Motl J, Ehrlich ME, Hurd YL, Roussos P: **An atlas of chromatin accessibility in the adult human brain**. *Genome Research* 2018, **28**(8):1243-1252.
65. Thurman RE, Rynes E, Humbert R, Vierstra J, Maurano MT, Haugen E, Sheffield NC, Stergachis AB, Wang H, Vernot B *et al*: **The accessible chromatin landscape of the human genome**. In: *Nature* 2012, **489**(7414):75-82.

66. Hor CN, Yeung J, Jan M, Emmenegger Y, Hubbard J, Xenarios I, Naef F, Franken P: **Sleep-wake-driven and circadian contributions to daily rhythms in gene expression and chromatin accessibility in the murine cortex**. *Proc Natl Acad Sci U S A* 2019, **116**(51):25773-25783.
67. Qureshi IA, Mehler MF: **Genetic and epigenetic underpinnings of sex differences in the brain and in neurological and psychiatric disease susceptibility**. *Prog Brain Res* 2010, **186**:77-95.
68. Forger NG: **Epigenetic mechanisms in sexual differentiation of the brain and behaviour**. *Philos Trans R Soc Lond B Biol Sci* 2016, **371**(1688):20150114.
69. Sugathan A, Waxman DJ: **Genome-wide analysis of chromatin states reveals distinct mechanisms of sex-dependent gene regulation in male and female mouse liver**. *Mol Cell Biol* 2013, **33**(18):3594-3610.
70. Frazier PI: **A Tutorial on Bayesian Optimization**. https://arxiv.org/abs/1807.02811 (2018). Accessed November 2019.
71. Bergstra J, Yamins D, Cox D: **Making a Science of Model Search: Hyperparameter Optimization in Hundreds of Dimensions for Vision Architectures**. *Proceedings of the 30th International Conference on Machine Learning*, 2013:**28**(1):115-123.
72. Snoek J, Larochelle H, Adams RP: **Practical Bayesian Optimization of Machine Learning Algorithms**. *Advances in Neural Information Processing Systems*, 2012:**2**:2951-2959.
73. Chen L, Fish AE, Capra JA: **Prediction of gene regulatory enhancers across species reveals evolutionarily conserved sequence properties**. *PLoS Comput Biol* 2018, **14**(10):e1006484.
74. Ghandi M, Lee D, Mohammad-Noori M, Beer MA: **Enhanced regulatory sequence prediction using gapped k-mer features**. *PLoS Comput Biol* 2014, **10**(7):e1003711.
75. Arvey A, Agius P, Noble WS, Leslie C: **Sequence and chromatin determinants of cell-type-specific transcription factor binding**. *Genome Research* 2012, **22**(9):1723-1734.
76. Lundberg SM, Lee S-I: **A Unified Approach to Interpreting Model Predictions**. In: *Advances in Neural Information Processing Systems: 2019*; 2019: 4765-4774.
77. Nair S, Kim DS, Perricone J, Kundaje A: **Integrating regulatory DNA sequence and gene expression to predict genome-wide chromatin accessibility across cellular contexts**. *Bioinformatics* 2019, **35**(14):i108-i116.
78. Cofer EM, Raimundo J, Tadych A, Yamazaki Y, Wong AK, Theesfeld CL, Levine MS, Troyanskaya OG: **Modeling transcriptional regulation of model species with deep learning**. *Genome Research* 2021, **31**(6):1097-1105.
79. Kelley DR: **Cross-species regulatory sequence activity prediction**. *PLoS Comput Biol* 2020, **16**(7):e1008050.
80. Blankvoort S, Witter MP, Noonan J, Cotney J, Kentros C: **Marked Diversity of Unique Cortical Enhancers Enables Neuron-Specific Tools by Enhancer-Driven Gene Expression**. *Curr Biol* 2018, **28**(13):2103-2114.e2105.
81. Lopes-Ramos CM, Paulson JN, Chen CY, Kuijjer ML, Fagny M, Platig J, Sonawane AR, DeMeo DL, Quackenbush J, Glass K: **Regulatory network changes between cell lines and their tissues of origin**. *BMC Genomics* 2017, **18**(1):723.
82. Kapopoulou A, Mathew L, Wong A, Trono D, Jensen JD: **The evolution of gene expression and binding specificity of the largest transcription factor family in primates.** *Evolution; international journal of organic evolution* 2016, **70**:167-180.
83. Imbeault M, Helleboid P-Y, Trono D: **KRAB zinc-finger proteins contribute to the evolution of gene regulatory networks**. *Nature* 2017, **543**:550-554.
84. Mitsis T, Efthimiadou A, Bacopoulou F, Vlachakis D, Chrousos G, Eliopoulos E: **Transcription factors and evolution: An integral part of gene expression (Review)**. *World Academy of Sciences Journal* 2020, **2**(1):3-8.

85. Diehl AG, Boyle AP: **Conserved and species-specific transcription factor co-binding patterns drive divergent gene regulation in human and mouse**. *Nucleic Acids Res* 2018, **46**(4):1878-1894.
86. Dukler N, Huang YF, Siepel A: **Phylogenetic Modeling of Regulatory Element Turnover Based on Epigenomic Data**. *Mol Biol Evol* 2020, **37**(7):2137-2152.
87. Madisen L, Zwingman TA, Sunkin SM, Oh SW, Zariwala HA, Gu H, Ng LL, Palmiter RD, Hawrylycz MJ, Jones AR *et al*: **A robust and high-throughput Cre reporting and characterization system for the whole mouse brain**. *Nat Neurosci* 2010, **13**(1):133-140.
88. Buenrostro JD, Giresi PG, Zaba LC, Chang HY, Greenleaf WJ: **Transposition of native chromatin for fast and sensitive epigenomic profiling of open chromatin, DNA-binding proteins and nucleosome position.** *Nature Methods* 2013, **10**:1213-1218.
89. Buenrostro JD, Wu B, Chang HY, Greenleaf WJ: **ATAC-seq: A Method for Assaying Chromatin Accessibility Genome-Wide**. *Curr Protoc Mol Biol* 2015, **109**:21.29.21-29.
90. ENCODE Project Consortium: **An integrated encyclopedia of DNA elements in the human genome.** *Nature* 2012, **489**:57-74.
91. Halstead MM, Kern C, Saelao P, Wang Y, Chanthavixay G, Medrano JF, Van Eenennaam AL, Korf I, Tuggle CK, Ernst CW *et al*: **A comparative analysis of chromatin accessibility in cattle, pig, and mouse tissues**. *BMC Genomics* 2020, **21**(1):698.
92. Lee JW, Foo CS, Kim D, Boley N, Kundaje A: **ATAC-Seq / DNase-Seq Pipeline**. https://github.com/kundajelab/atac_dnase_pipelines (2015). Accessed December 2017.
93. Lander ES, Linton LM, Birren B, Nusbaum C, Zody MC, Baldwin J, Devon K, Dewar K, Doyle M, FitzHugh W *et al*: **Initial sequencing and analysis of the human genome**. *Nature* 2001, **409**:860-921.
94. Amemiya HM, Kundaje A, Boyle AP: **The ENCODE Blacklist: Identification of Problematic Regions of the Genome**. *Sci Rep* 2019, **9**(1):9354.
95. Davis CA, Hitz BC, Sloan CA, Chan ET, Davidson JM, Gabdank I, Hilton JA, Jain K, Baymuradov K, Narayanan AK: **The Encyclopedia of DNA elements (ENCODE): data portal update**. *Nucleic Acids Res* 2018, 46(D1):D794-D801.
96. Li Q, Brown JB, Huang H, Bickel PJ: **Measuring reproducibility of high-throughput experiments**. *Annals of Applied Statistics* 2011, **5**:1752-1779.
97. **ENCODE ATAC-seq Pipeline**. https://github.com/ENCODE-DCC/atac-seq-pipeline (2018). Accessed June 2019.
98. Gibbs RA, Rogers J, Katze MG, Bumgarner R, Weinstock GM, Mardis ER, Remington KA, Strausberg RL, Venter JC, Wilson RK *et al*: **Evolutionary and biomedical insights from the rhesus macaque genome**. *Science* 2007, **316**(5822):222-234.
99. Waterston RH, Lindblad-Toh K, Birney E, Rogers J, Abril JF, Agarwal P, Agarwala R, Ainscough R, Alexandersson M, An P *et al*: **Initial sequencing and comparative analysis of the mouse genome**. *Nature* 2002, **420**:520-562.
100. Gibbs RA, Weinstock GM, Metzker ML, Muzny DM, Sodergren EJ, Scherer S, Scott G, Steffen D, Worley KC, Burch PE *et al*: **Genome sequence of the Brown Norway rat yields insights into mammalian evolution**. *Nature* 2004, **428**(6982):493-521.
101. Hughes JF, Skaletsky H, Pyntikova T, Koutseva N, Raudsepp T, Brown LG, Bellott DW, Cho TJ, Dugan-Rocha S, Khan Z *et al*: **Sequence analysis in**. *Genome Res* 2020, **30**(12):1716-1726.
102. Groenen MA, Archibald AL, Uenishi H, Tuggle CK, Takeuchi Y, Rothschild MF, Rogel-Gaillard C, Park C, Milan D, Megens HJ *et al*: **Analyses of pig genomes provide insight into porcine demography and evolution**. *Nature* 2012, **491**(7424):393-398.
103. Landt SG, Marinov GK, Kundaje A, Kheradpour P, Pauli F, Batzoglou S, Bernstein BE, Bickel P, Brown JB, Cayting P *et al*: **ChIP-seq guidelines and practices of the ENCODE and modENCODE consortia**. *Genome Research* 2012, **22**(9):1813-1831.

104. John S, Sabo PJ, Canfield TK, Lee K, Vong S, Weaver M, Wang H, Vierstra J, Reynolds AP, Thurman RE *et al*: **Genome-scale mapping of DNase I hypersensitivity**. *Curr Protoc Mol Biol* 2013, **Chapter 27**:Unit 21.27.
105. Quinlan AR, Hall IM: **BEDTools: a flexible suite of utilities for comparing genomic features.** *Bioinformatics* 2010, **26**:841-842.
106. Harrow J, Frankish A, Gonzalez JM, Tapanari E, Diekhans M, Kokocinski F, Aken BL, Barrell D, Zadissa A, Searle S *et al*: **GENCODE: The reference human genome annotation for the ENCODE project**. *Genome Research* 2012, **22**(9):1760-1774.
107. Frankish A, Diekhans M, Ferreira AM, Johnson R, Jungreis I, Loveland J, Mudge JM, Sisu C, Wright J, Armstrong J *et al*: **GENCODE reference annotation for the human and mouse genomes**. *Nucleic Acids Res* 2019, **47**(D1):D766-D773.
108. O'Leary NA, Wright MW, Brister JR, Ciufo S, Haddad D, McVeigh R, Rajput B, Robbertse B, Smith-White B, Ako-Adjei D *et al*: **Reference sequence (RefSeq) database at NCBI: current status, taxonomic expansion, and functional annotation**. *Nucleic Acids Res* 2016, **44**(D1):D733-745.
109. Kent WJ, Baertsch R, Hinrichs A, Miller W, Haussler D: **Evolution's cauldron: duplication, deletion, and rearrangement in the mouse and human genomes**. *Proc Natl Acad Sci U S A* 2003, **100**(20):11484-11489.
110. Hickey G, Paten B, Earl D, Zerbino D, Haussler D: **HAL: a hierarchical format for storing and analyzing multiple genome alignments**. *Bioinformatics* 2013, **29**(10):1341-1342.
111. Armstrong J, Hickey G, Diekhans M, Fiddes IT, Novak AM, Deran A, Fang Q, Xie D, Feng S, Stiller J *et al*: **Progressive Cactus is a multiple-genome aligner for the thousand-genome era**. *Nature* 2020, **587**(7833):246-251.
112. Zhang X, Kaplow IM, Wirthlin M, Park TY, Pfenning AR: **HALPER facilitates the identification of regulatory element orthologs across species**. *Bioinformatics* 2020, **36**(15):4339-4340.
113. Zhang Y, Liu T, Meyer CA, Eeckhoute J, Johnson DS, Bernstein BE, Nussbaum C, Myers RM, Brown M, Li W *et al*: **Model-based Analysis of ChIP-Seq (MACS)**. *Genome Biol* 2008, **9**:R137.
114. Bailey TL, Machanick P: **Inferring direct DNA binding from ChIP-seq**. *Nucleic Acids Res* 2012, **40**(17):e128.
115. Huh I, Mendizabal I, Park T, Yi SV: **Functional conservation of sequence determinants at rapidly evolving regulatory regions across mammals**. *PLoS Comput Biol* 2018, **14**(10):e1006451.
116. Minnoye L, Taskiran II, Mauduit D, Fazio M, Van Aerschot L, Hulselmans G, Christiaens V, Makhzami S, Seltenhammer M, Karras P *et al*: **Cross-species analysis of enhancer logic using deep learning**. *Genome Research* 2020, **30**(12):1815-1834.
117. Dale RK, Pedersen BS, Quinlan AR: **Pybedtools: A flexible Python library for manipulating genomic datasets and annotations**. *Bioinformatics* 2011, **27**(24):3423-3424.
118. Stamatoyannopoulos JA, Snyder M, Hardison R, Ren B, Gingeras T, Gilbert DM, Groudine M, Bender M, Kaul R, Canfield T *et al*: **An encyclopedia of mouse DNA elements (Mouse ENCODE)**. *Genome Biol* 2012, **13**(8):418.
119. Pagès H: ***BSgenome: Software infrastructure for efficient representation of full genomes and their SNPs.*** https://bioconductor.org/packages/release/bioc/html/BSgenome.html (2006). Accessed January 2020.
120. Kent WJ, Sugnet CW, Furey TS, Roskin KM, Pringle TH, Zahler AM, Haussler D: **The Human Genome Browser at UCSC**. *Genome Research* 2002: **12**(6):996-1006.
121. Ghandi M, Mohammad-Noori M, Ghareghani N, Lee D, Garraway L, Beer MA: **gkmSVM: an R package for gapped-kmer SVM**. *Bioinformatics* 2016, **32**(14):2205-2207.
122. Bailey TL, Boden M, Buske FA, Frith M, Grant CE, Clementi L, Ren J, Li WW, Noble WS: **MEME Suite: Tools for motif discovery and searching**. *Nucleic Acids Res* 2009, **37**:W202-W208.

123. Patrushev LI, Kovalenko TF: **Functions of noncoding sequences in mammalian genomes**. *Biochemistry (Mosc)* 2014, **79**(13):1442-1469.
124. Chollet F: **Keras**. https://keras.io (2015). Accessed August 2018.
125. Bergstra J, Breuleux O, Bastien F, Lamblin P, Pascanu R, Desjardins G, Turian D, Warde-Farley D, Bengio: **Theano: A CPU and GPU Math Expression Compiler**. *Proceedings of the Python for Scientific Computing Conference* 2010.
126. Pedregosa F, Varoquaux G: **Scikit-learn: Machine learning in Python**. *Journal of Machine Learning Research* 2011, **12**:2825-2830.
127. Grau J, Grosse I, Keilwagen J: **PRROC: Computing and visualizing Precision-recall and receiver operating characteristic curves in R**. *Bioinformatics* 2015, **31**(15):2595-2597.
128. Gupta S, Stamatoyannopoulos JA, Bailey TL, Noble W: **Quantifying similarity between motifs**. *Genome Biol* 2007, **8**:R24.
129. Weirauch MT, Yang A, Albu M, Cote AG, Montenegro-Montero A, Drewe P, Najafabadi HS, Lambert SA, Mann I, Cook K *et al*: **Determination and Inference of Eukaryotic Transcription Factor Sequence Specificity**. *Cell* 2014, **158**:1431-1443.
130. Lee JW, Boley N, Kundaje A: **AQUAS TF and histone ChIP-seq pipeline**. https://github.com/kundajelab/chipseq_pipeline (2015). Accessed September 2017.
131. Li D, Hsu S, Purushotham D, Sears RL, Wang T: **WashU Epigenome Browser update 2019**. *Nucleic Acids Res* 2019, **47**(W1):W158-W165.
132. Keesey TM: **PhyloPic**. http://phylopic.org/ (2019). Accessed May 2019.
133. Blanchette M, Kent WJ, Riemer C, Elnitski L, Smit AF, Roskin KM, Baertsch R, Rosenbloom K, Clawson H, Green ED *et al*: **Aligning multiple genomic sequences with the threaded blockset aligner**. *Genome Research* 2004, **14**(4):708-715.
134. Tyner C, Barber GP, Casper J, Clawson H, Diekhans M, Eisenhart C, Fischer CM, Gibson D, Gonzalez JN, Guruvadoo L *et al*: **The UCSC Genome Browser database: 2017 update**. *Nucleic Acids Res* 2017, **45**(D1):D626-D634.

Supplemental Figure 1

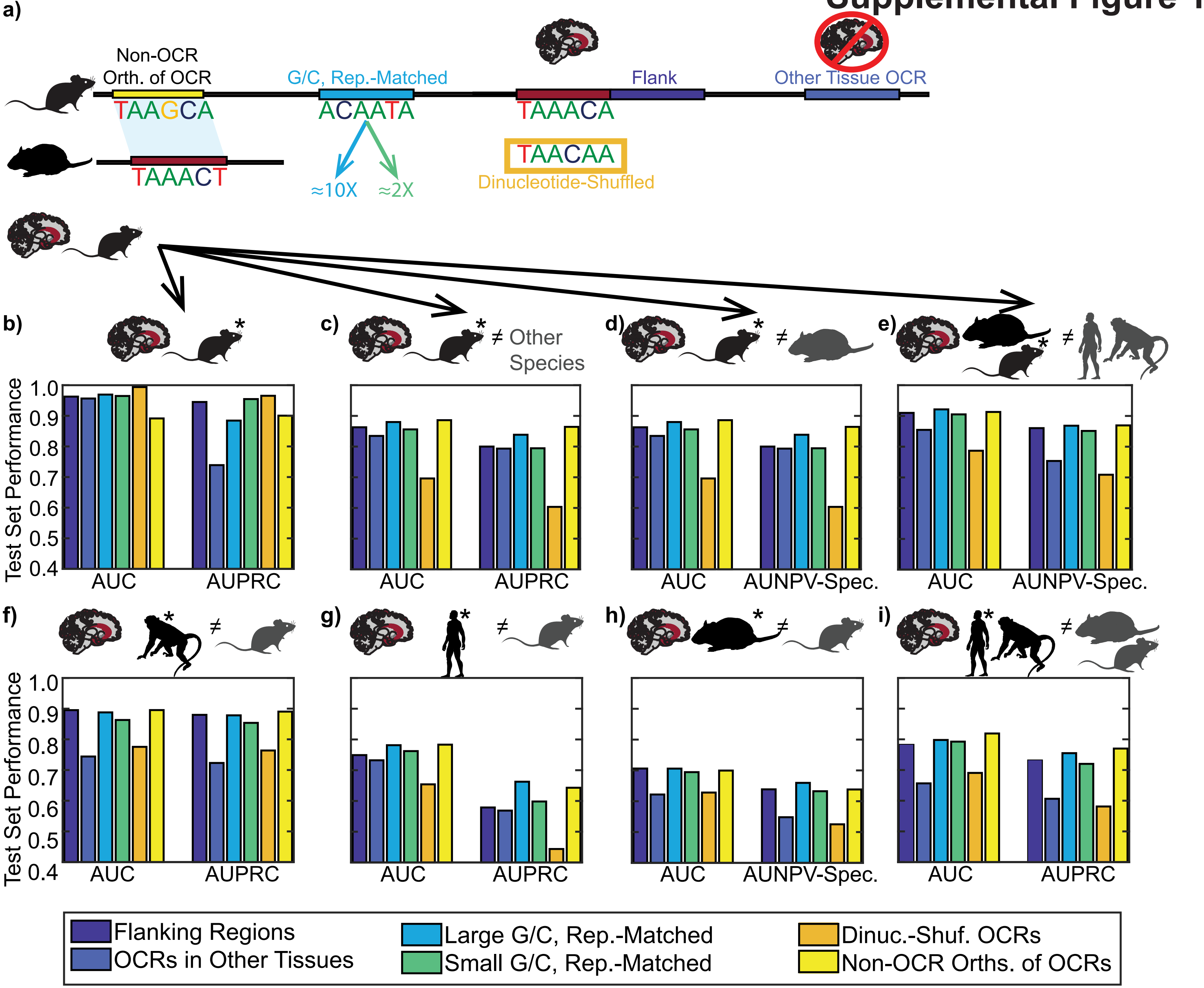

Supplemental Figure 2

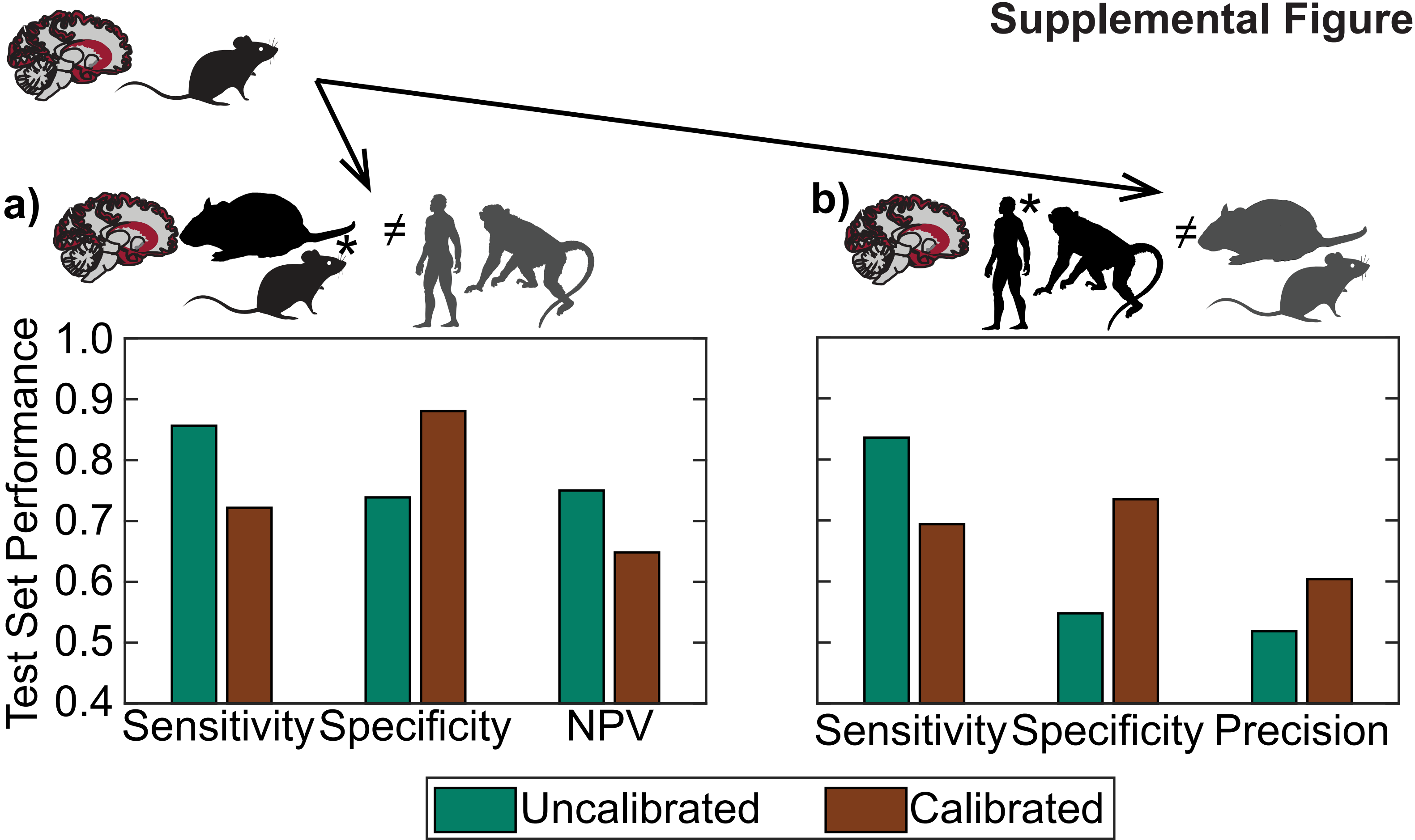

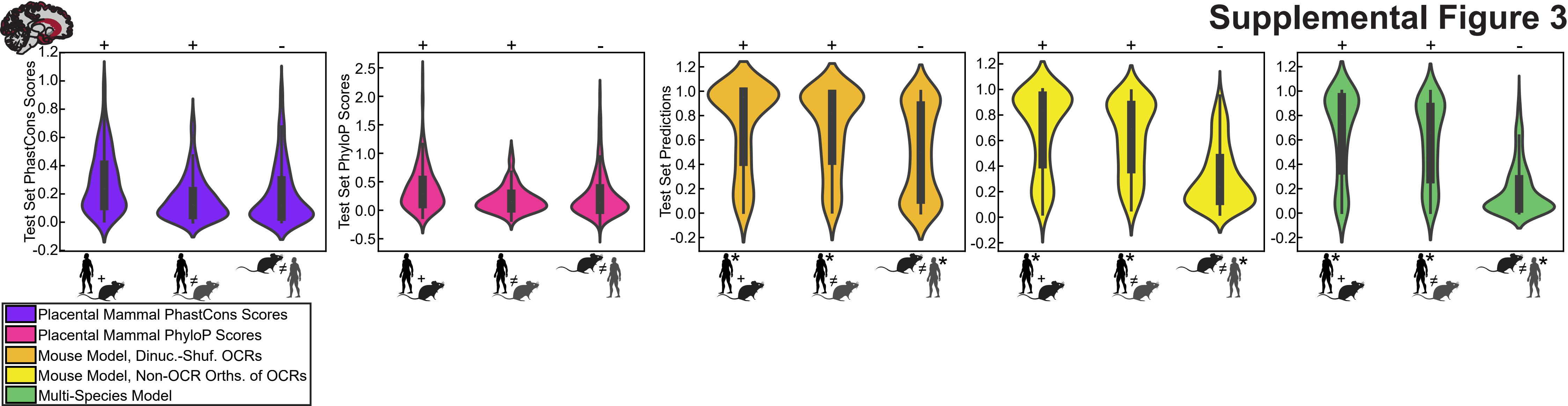
Supplemental Figure 3
Test Set PhastCons Scores
Test Set PhyloP Scores
Test Set Predictions
Placental Mammal PhastCons Scores
Placental Mammal PhyloP Scores
Mouse Model, Dinuc.-Shuf. OCRs
Mouse Model, Non-OCR Orths. of OCRs
Multi-Species Model

Supplemental Figure 4

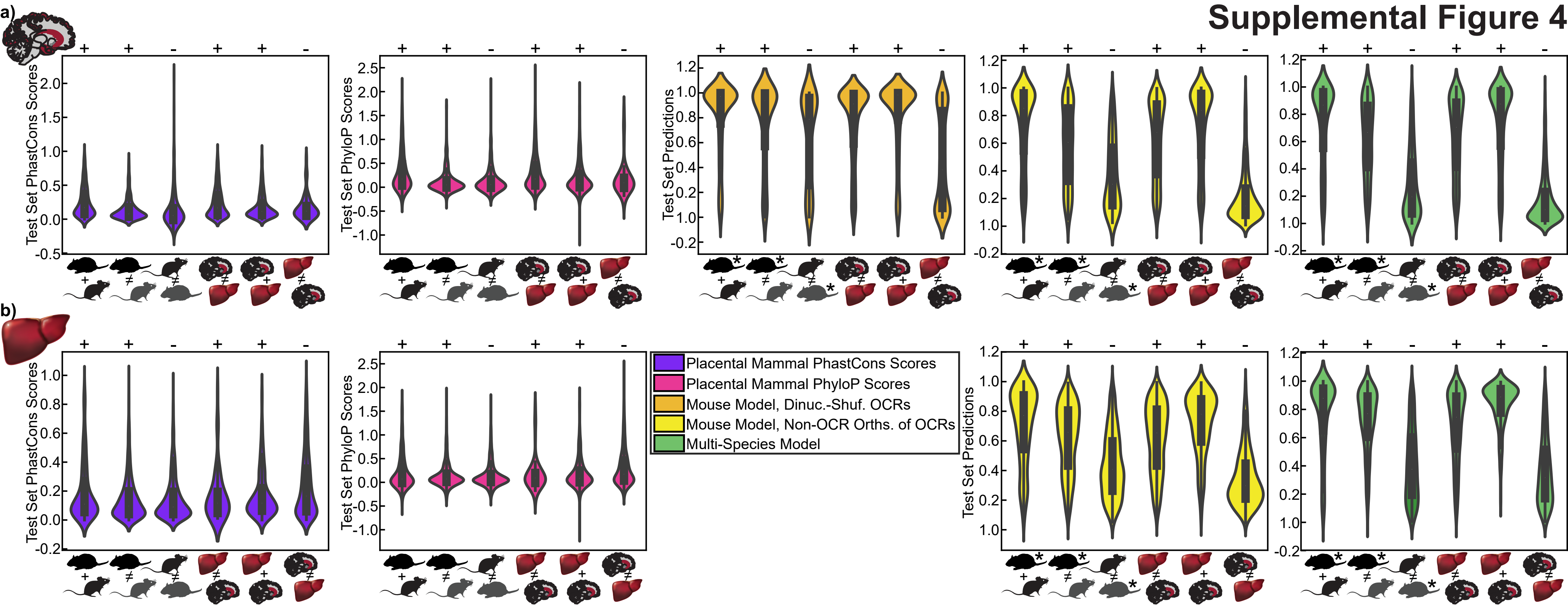

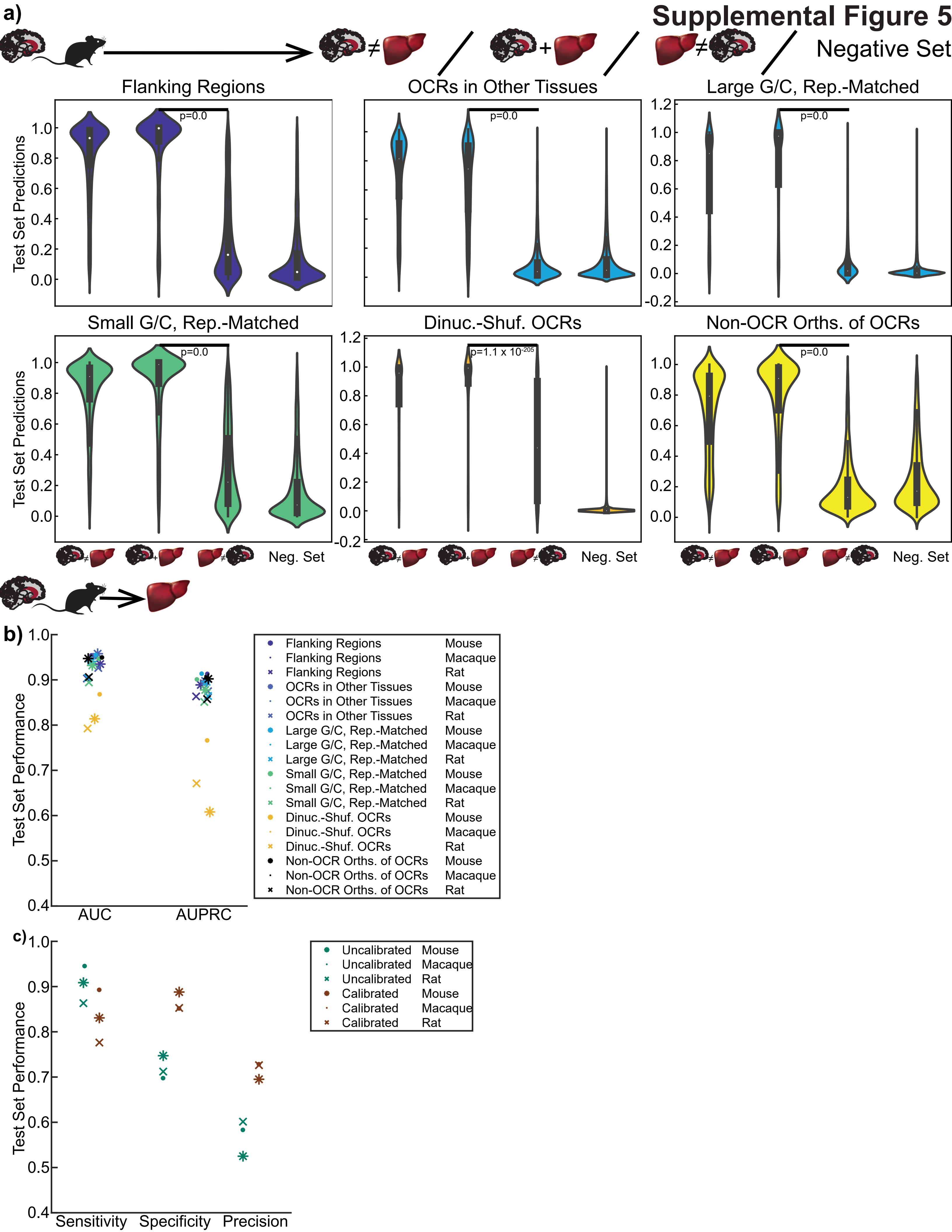
Supplemental Figure 5
a)
Negative Set
Flanking Regions
OCRs in Other Tissues
Large G/C, Rep.-Matched
Small G/C, Rep.-Matched
Dinuc.-Shuf. OCRs
Non-OCR Orths. of OCRs
p=0.0
p=1.1 x 10-205
Test Set Predictions
Neg. Set
b)
Test Set Performance
AUC
AUPRC
Flanking Regions Mouse
Flanking Regions Macaque
Flanking Regions Rat
OCRs in Other Tissues Mouse
OCRs in Other Tissues Macaque
OCRs in Other Tissues Rat
Large G/C, Rep.-Matched Mouse
Large G/C, Rep.-Matched Macaque
Large G/C, Rep.-Matched Rat
Small G/C, Rep.-Matched Mouse
Small G/C, Rep.-Matched Macaque
Small G/C, Rep.-Matched Rat
Dinuc.-Shuf. OCRs Mouse
Dinuc.-Shuf. OCRs Macaque
Dinuc.-Shuf. OCRs Rat
Non-OCR Orths. of OCRs Mouse
Non-OCR Orths. of OCRs Macaque
Non-OCR Orths. of OCRs Rat
c)
Test Set Performance
Sensitivity
Specificity
Precision
Uncalibrated Mouse
Uncalibrated Macaque
Uncalibrated Rat
Calibrated Mouse
Calibrated Macaque
Calibrated Rat

Supplemental Figure 6

a)

TF-MoDISco Motif	TFs with Similar Motif	Seqlets
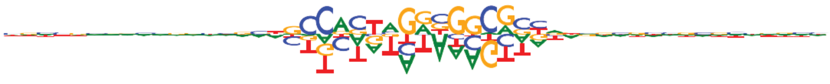	Ctcf, Ctcfl	267
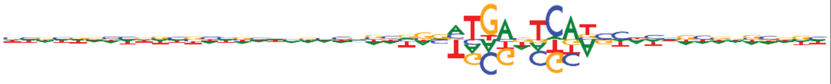	Fos, Smarcc1, Fosb, Jund	143
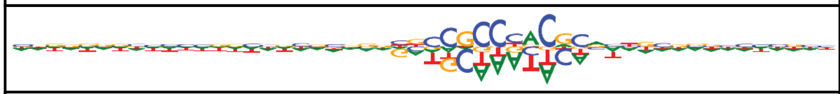	Egr2	137
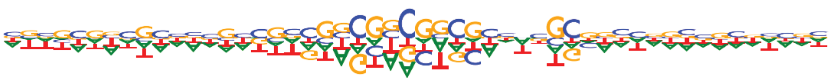		97
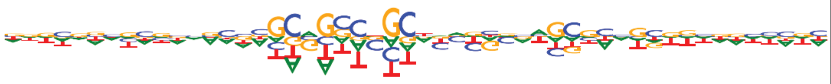		77
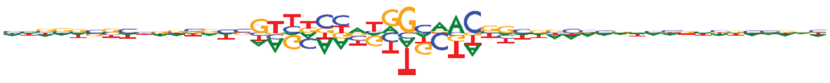	Rfx1, Arid2, Rfx2, Rfx4, Rfx7, Rfx5, Rfx3	35
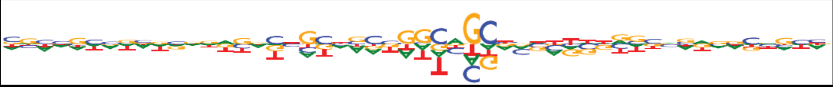		31
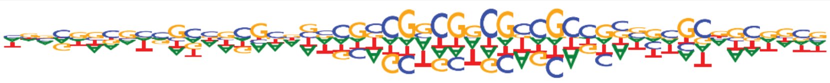		28

b)

TF-MoDISco Motif	TFs with Similar Motif	Seqlets
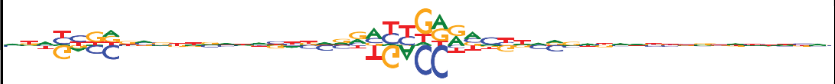	Rxra, Hnf4g, Nr4a3, Ppara, Nr1h2	340
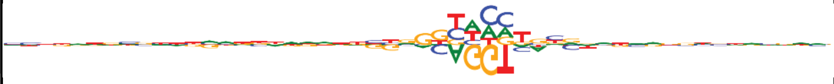		71
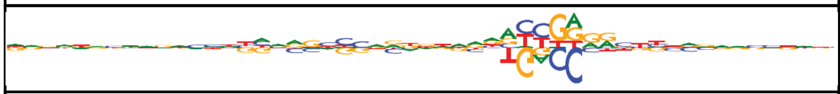	Nr4a3, Rxra, Nr4a2, Nr4a1, Ppara, Nr5a1	69
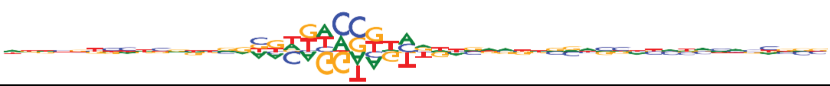	Erg, Fev, Bcl11a, Elk3, Elk1, Fli1, Erf, Etv3, Elk4, Etv6, Etv5, Elf4, Gm4881, Etv1, Etv4, Gm5454, Elf2, Etv2, Ets1, Spfi1, Zkscan5, Elf3, Ehf	39
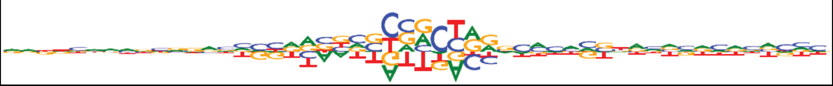	Foxc1, Foxc2, Foxl1, Foxp1, Foxb1, Foxp2, Foxj2, Foxo3, Foxj3, Foxd1, Foxk1, Foxg1, Foxf2, Foxo4, Foxd2, ENSMUSG00000090020, Foxn3, Foxo6, Foxa2, Gm5294, Foxp4, Tbpl2	44
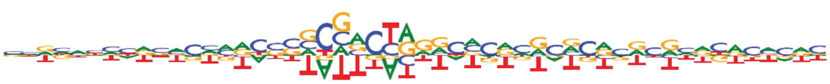		35
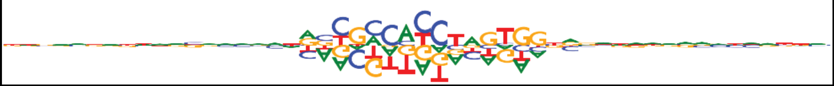	Ctcf, Ctcfl	169
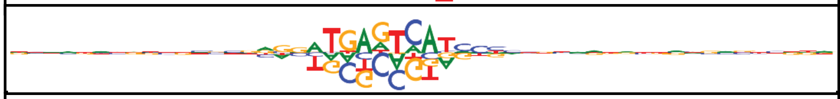	Fos, Jund, Smarcc1, Fosb	143
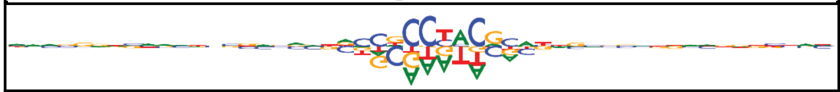		101
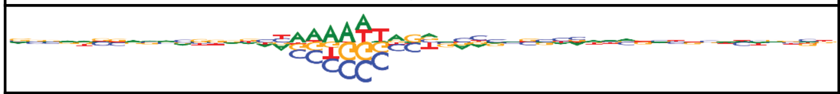	Mef2c, Mef2a, Mef2d, Mef2b	36
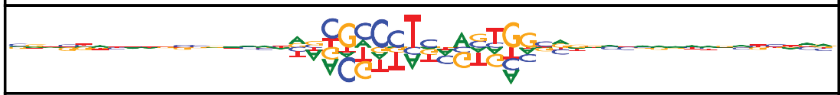	Ctcf, Ctcfl, Twist1	29
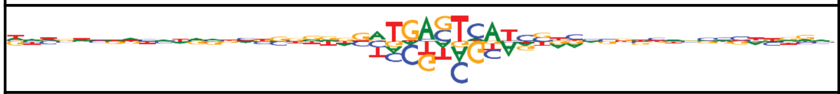		24
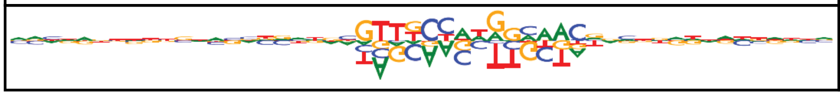	Rfx1, Rfx2, Rfx4, Arid2, Rfx7, Rfx5, Rfx3	38

c)

TF-MoDISco Motif	TFs with Similar Motif	Seqlets
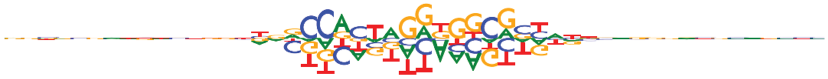	Ctcf, Ctcfl	275
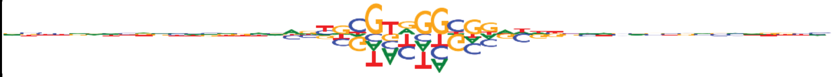	Egr3, Egr2, Bcl6	190
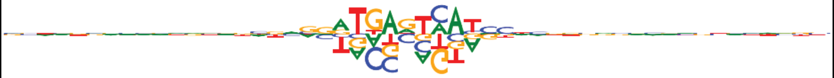	Fos, Jund, Fosb, Smarcc1	160
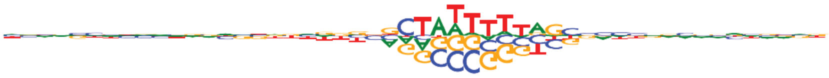	Mef2a, Mef2c, Mef2d, Mef2b	109
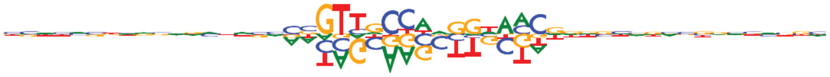	Rfx1, Rfx5, Rfx4, Arid2, Rfx2, Rfx7, Rfx3	43
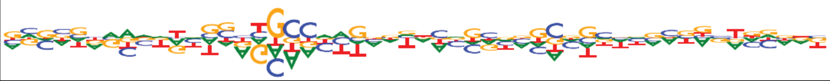		32
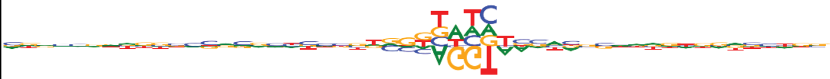		38
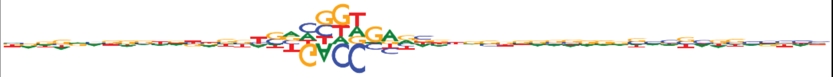	Ppara, Rxrb, Nr2f2, Rara, Nr2f1, Nr2c1, Rxrg, Esrra, Nr2f6	24

d)

TF-MoDISco Motif	TFs with Similar Motif	Seqlets
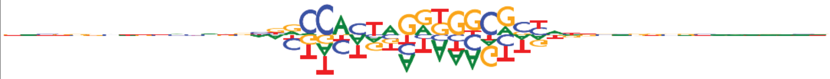	Ctcf, Ctcfl	301
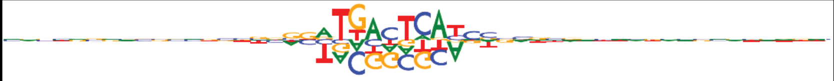	Fos, Jund, Fosb, Smarcc1	175
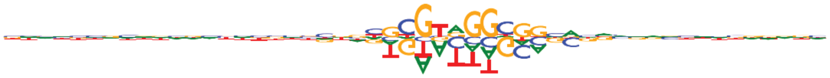	Egr2, Hif3a, Egr3, Bcl6, E2f3, Ets1	167
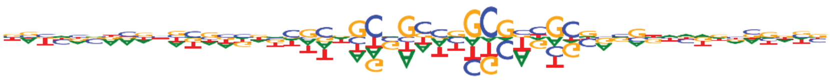		53
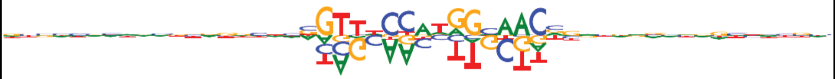	Rfx1, Rfx2, Arid2, Rfx4, Rfx7, Rfx5, Rfx3	47
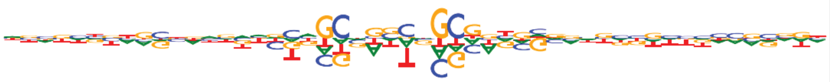		31
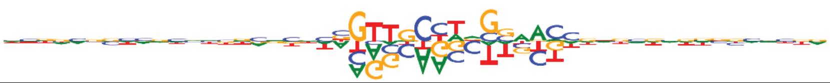	Rfx5, Rfx4, Rfx7, Rfx8, Rfx3, Rfx1, Stat1	29
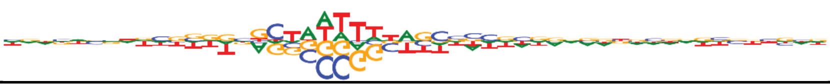	Mef2c, Mef2a, Mef2d, Mef2b	55

e)

TF-MoDISco Motif	TFs with Similar Motif	Seqlets
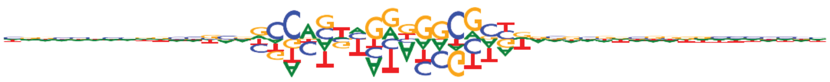	Ctcf, Ctcfl	250
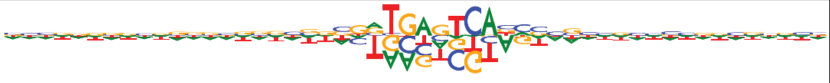	Fos, Fosb, Jund, Smarcc1	110
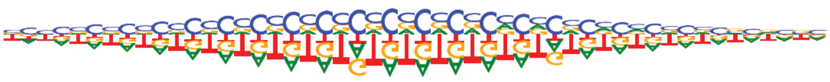		80
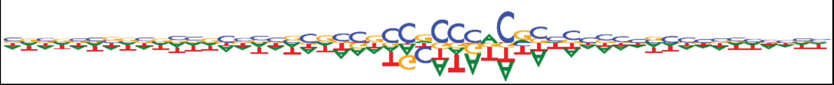	Egr2, Egr3, Zfp148, Maz, E2f3, Zfp281	58
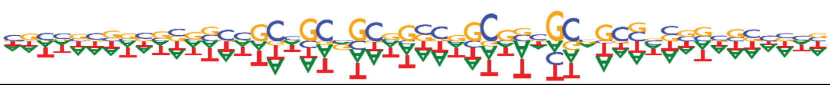		75
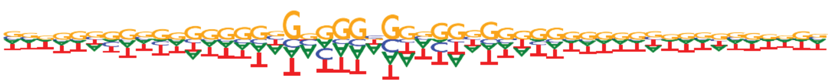	Maz, Bcl6, Sp3, Zfp148, Zfp281, Sp2, Wt1, E2f1, E2f3, Rreb1, Zbtb7a, Zfp219, E2f6, Klf15, Plag1	54
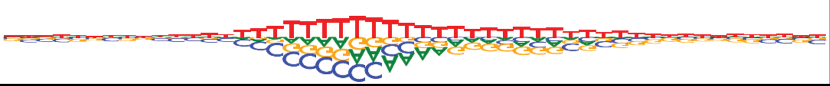		28
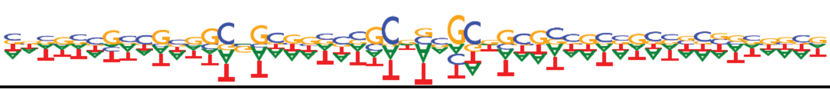		35
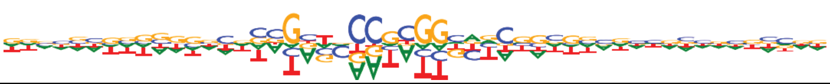	Rfx1, Arid2, Rfx2, Rfx4, Rfx5, Rfx7	27
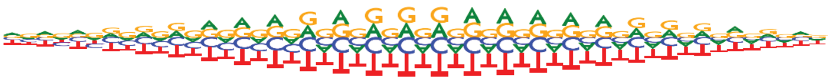	Bcl6	22

f)

TF-MoDISco Motif	TFs with Similar Motif	Seqlets
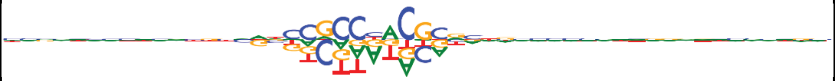	Egr2, Bcl6, Maz, Hif3a, Egr3, Rreb1, Zpf148, Zfp281, Ets1, Egr1, E2f3, Sp3, Sp1, Sp4, Zpf740	250
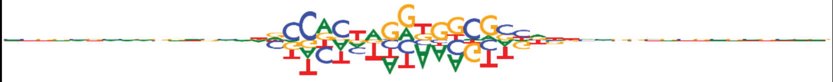	Ctcf, Ctcfl	110
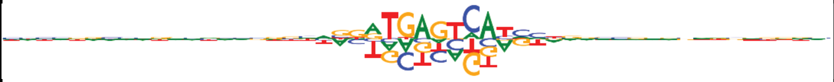	Fos, Fosb, Jund, Smarcc1, Junb, Bach1	80
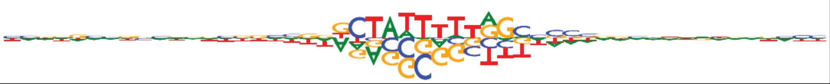	Mef2a, Mef2c, Mef2d, Mef2b	58
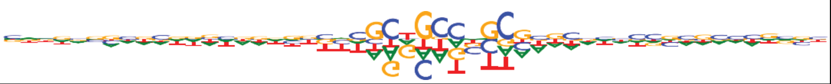		75
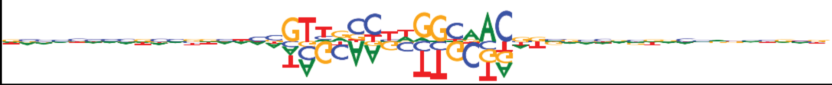	Rfx1, Rfx2, Arid2, Rfx4, Rfx7, Rfx5, Rfx3	54
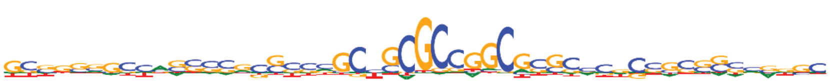		28
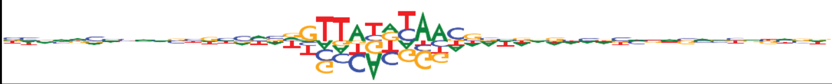	Dbp, Atf4, Tef, Nfil3	35
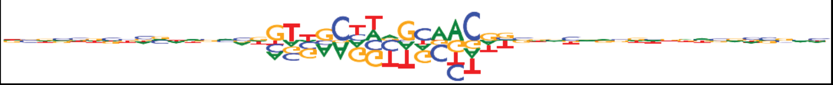	Rfx5, Rfx8, Rfx4, Rfx7	27
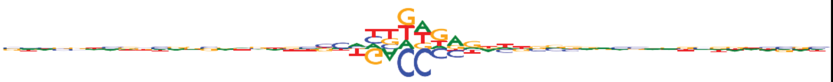	Thra	22

Supplemental Figure 7

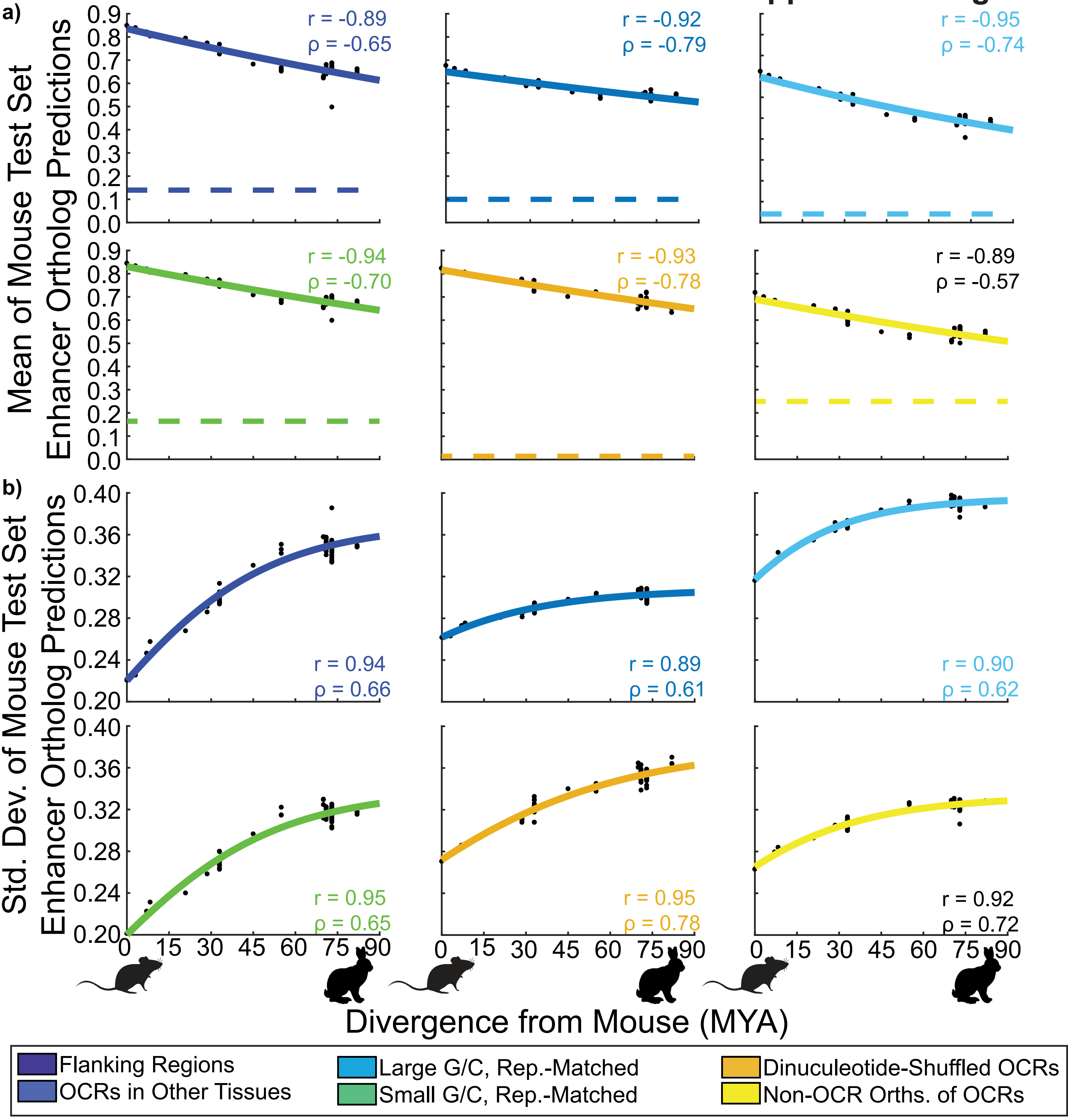

Supplemental Figure 8

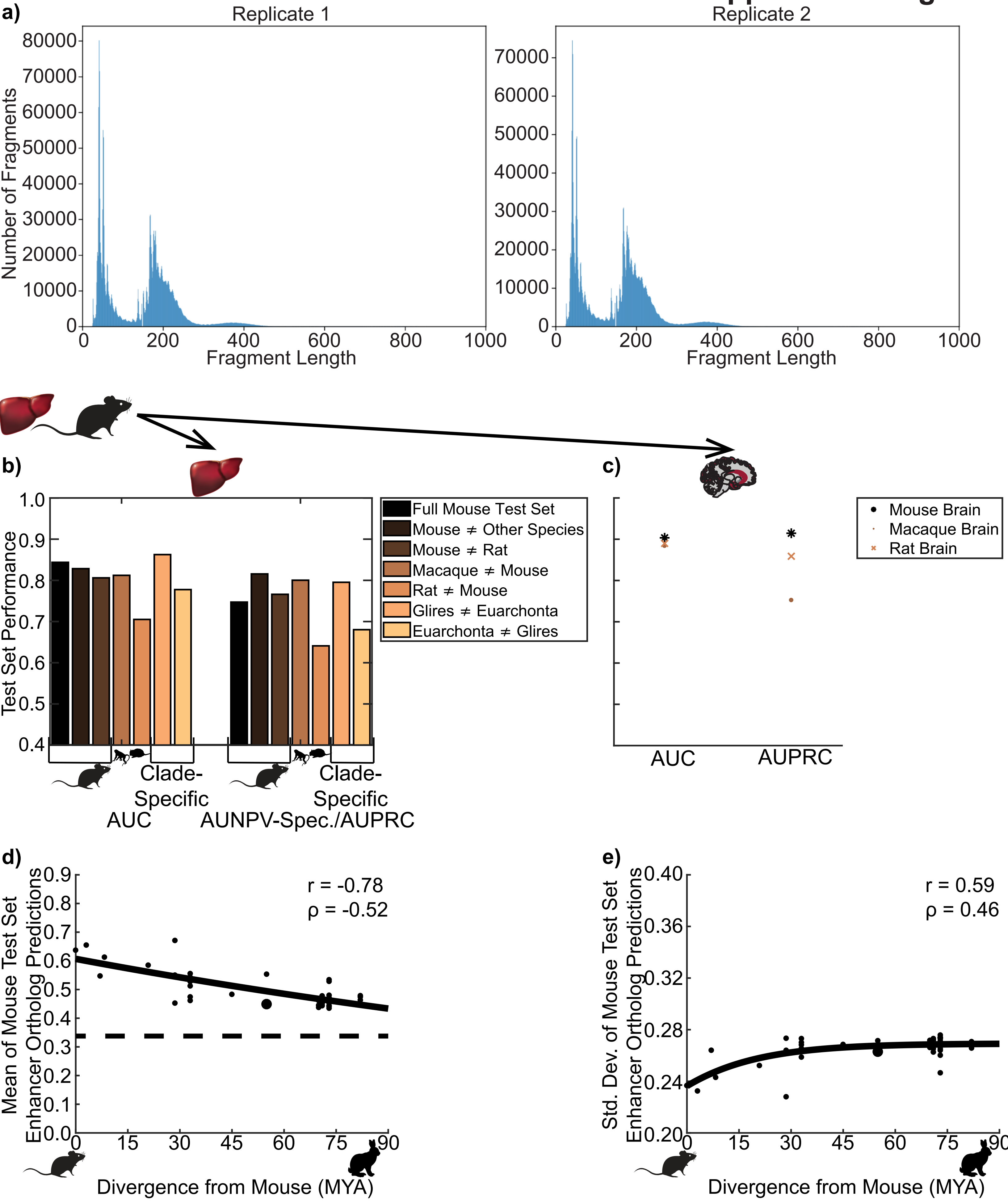

f)

TF-MoDISco Motif	TFs with Similar Motif	Seqlets
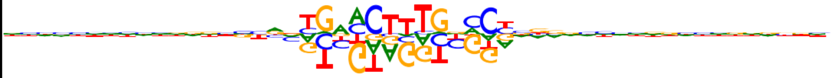	Hnf4g, Ppard, Nr2c2, Hnf4a, Nr4a2	612
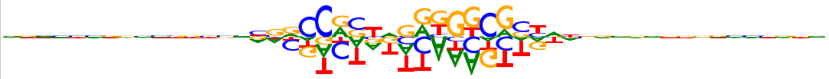	Ctcf, Zfp661	420
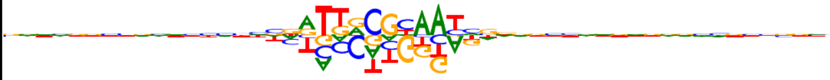	Cebpb, Cebpg, Cebpe, Tef, Cebpa	329
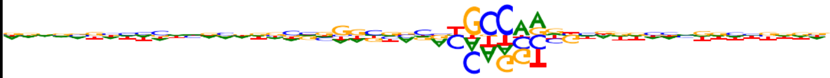		176
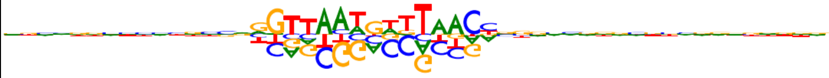		163
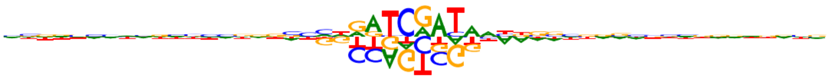	Onecut3, Onecut1, Onecut2	126
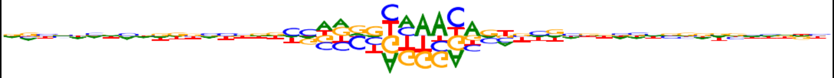	Foxa2, Foxi1, Foxp4, Foxc1, Foxc2, Foxb1, Foxn3	110
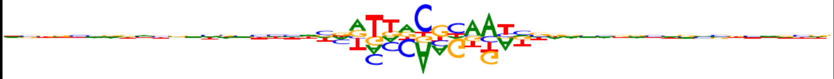	Cebpg, Cebpb, Cebpa	50
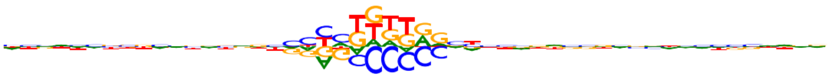		52
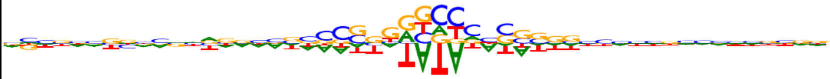		46
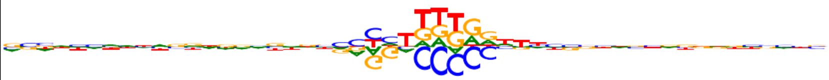	Klf15, Zfp281, Zfp341, Zfp770, Zpf641, Zfp467, Wt1, Rreb1, Plag1	40
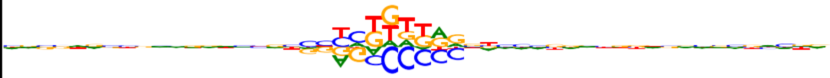		46
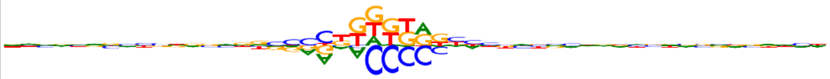	Plagl2, Zfp64, Plag1, Plagl1	30

Supplemental Figure 9

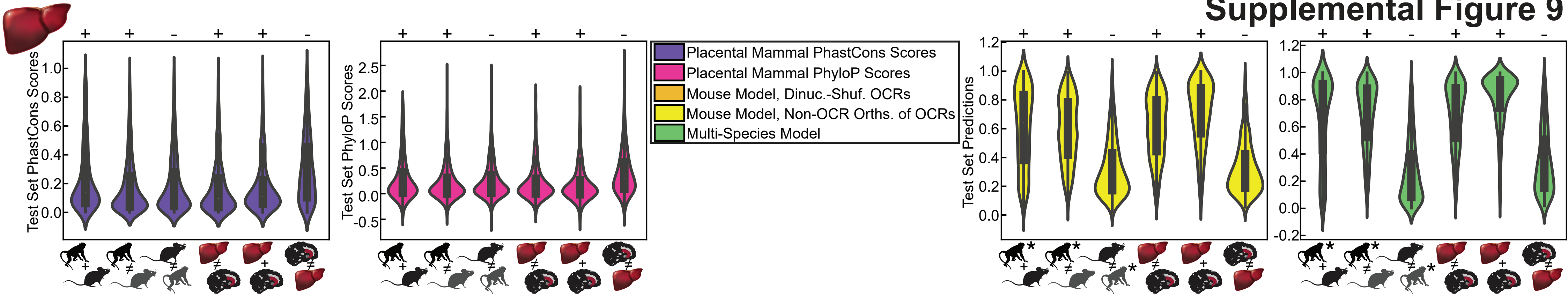

Supplemental Figure 10

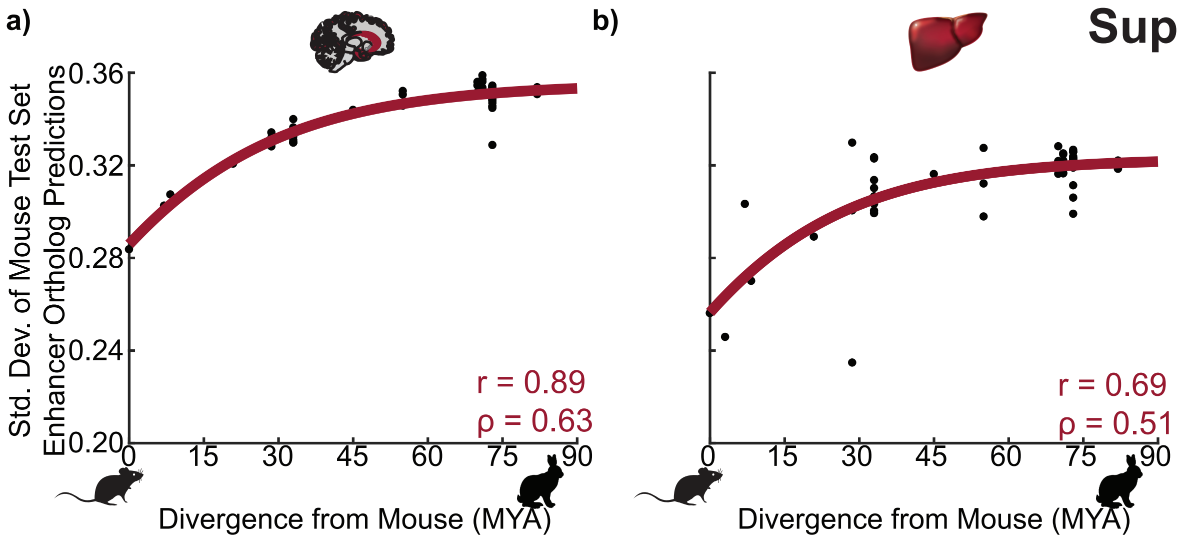

c)

Brain TF-MoDISco Motif	TFs with Similar Motif	Seqlets
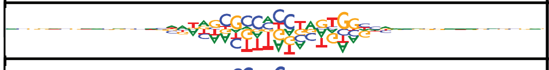	Ctcf, Ctcfl	1052
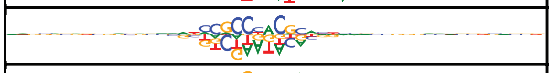	Egr2, Egr3, Bcl6, Hif3a	668
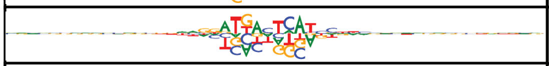	Fos, Smarcc1, Fosb	515
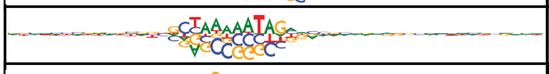	Mef2a, Mef2c, Mef2d, Mef2b, Tead4	220
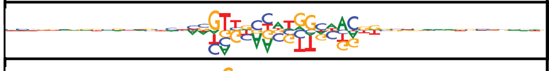	Rfx1, Rfx2, Arid2, Rfx5, Rfx4, Rfx7	156
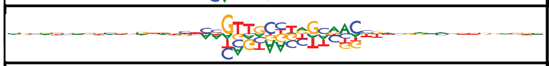	Rfx8, Rfx5, Rfx7	72
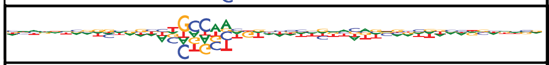		57
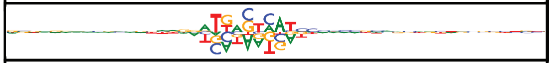		51
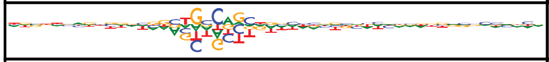		48
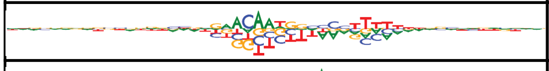		34
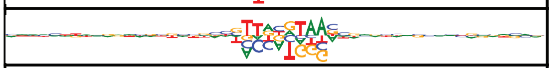	Hlf, Tef, Dbp, Nfil3, Cebpb	44
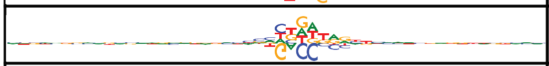	Gli3, Glis3, Rxrb, Gli1, Rxrg, Nr1i3, Zic5, Gli2	93
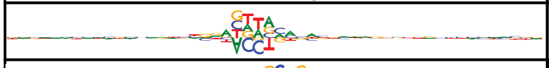		82
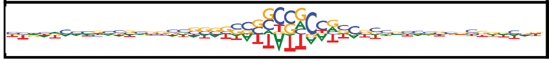	Onecut1, Onecut3, Hmg20b, Foxl1, Pit1	52

d)

Liver TF-MoDISco Motif	TFs with Similar Motif	Seqlets
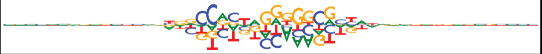	Ctcf, Ctcfl	1161
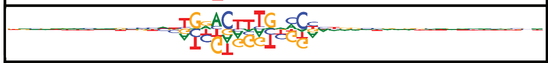	Hnf4g, Nr1h2, Rxra, Hnf4a, Nr4a3, Pparg	839
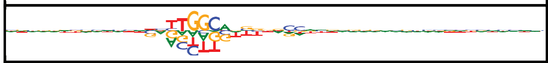		665
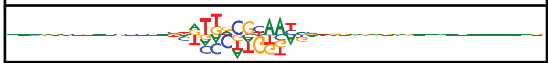	Cebpg, Cebpb, Cepbd, Cebpa, Cebpe, Dbp, Tef, Hlf, Nfil3	663
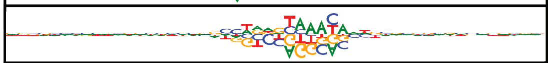	Foxa2, Foxf2, Foxp4, Foxc1, Foxc2, Foxb1, Foxa3, Foxa1, Foxj3, Foxd1, Foxp2, Foxo3, Foxn3 Foxl1, Foxp1, Foxk1, Foxj2, ENSMUSG00000090020, Foxg1, Foxd2, Foxd3, Gm5294, Foxj1	496
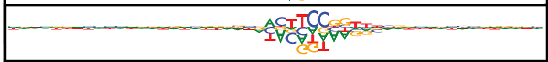	Ets1, Erg, Sfpi1, Bcl6, Spib, Fli1, Bcl11a, Elk1, Etv6, Etv1, Elk3, Etv4, Erf, Etv3, Gm5454, Gm4881, Elk4, Etv5, Elf4, Fev, Etv2, Elf2, Gabpa, Ets2, Spic, Elf3, Ehf, Maz, Prdm1, Elf5	356
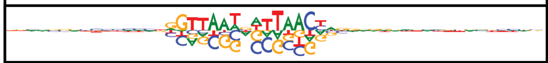		278
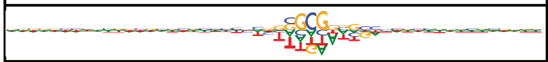	Sp3, Sp2, Zbtb7a, Zfp148, Wt1, Maz, Zfp281, Klf16, Klf5, Egr4, Sp1, Sp5, Zfx, E2f1, Klf6, Sp8, Klf2, Klf7, Klf14, Klf4, Klf15, Zfp219, Klf8, Klf12, Sp4, Egr2, E2f3, Tcfap2c, Mbd2, Rreb1	266
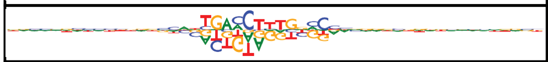	Hnf4g, Nr4a3, Ppara, Nr4a2, Rxra, Nr1h2, Nr4a1, Ppard, Pparg	215
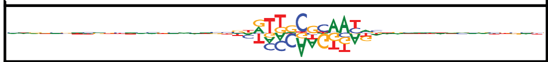		181
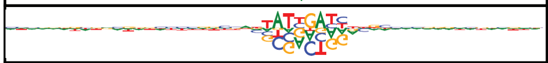	Onecut3, Onecut1	178
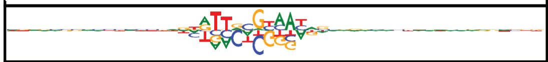	Bcl6	138
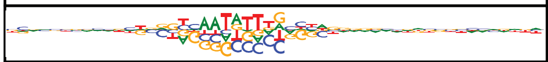		119
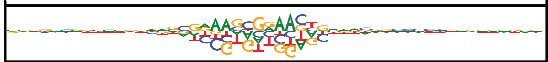	Irf1, Stat2, Prdm1, Bcl11a, Bcl6, Sfpi1, Irf2	95
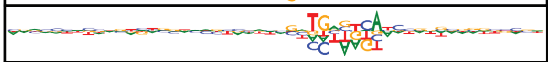	Bach1, Fosb, Nfe2l2	35
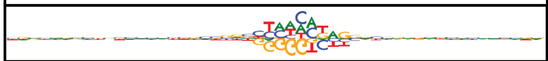		132
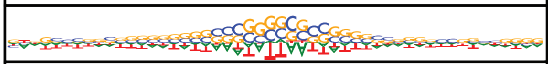	Phf21a, Arid3b, Dbx1, Tlx2, Lhx3, Pou3f4, Pit1, Lhx5, Onecut1, Pou3f1, Lmx1b, Lhx1, Pouf43, Pou1f1, Hmg20b, Onecut3, Pou2f1, Lmx1a, Pou4f1	90
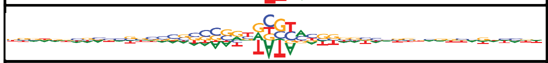	Zfp637	38
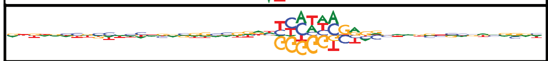		35
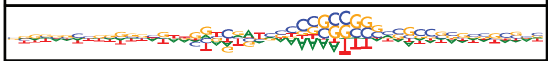		28

Supplemental Figure 11

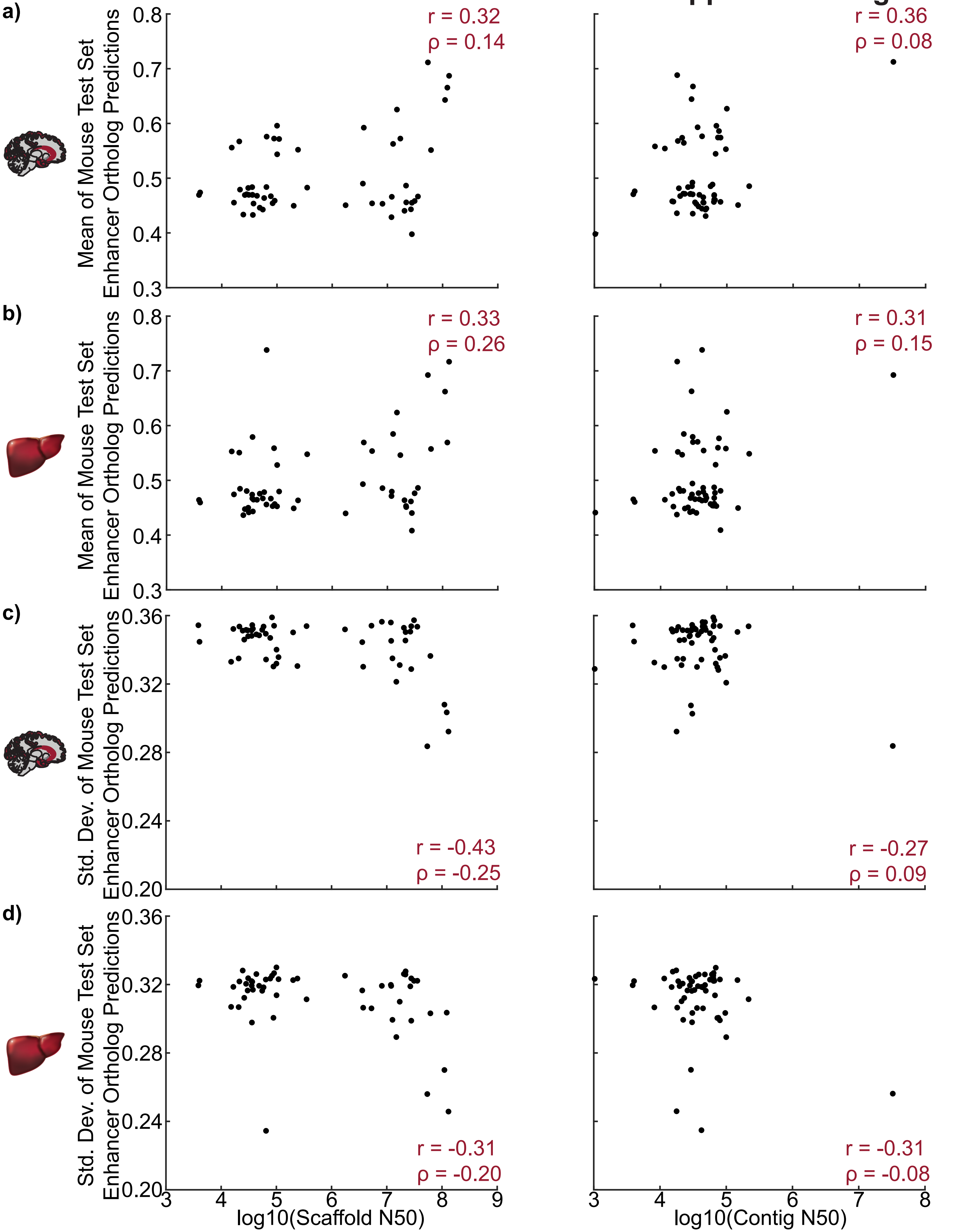

Supplemental Figure 12

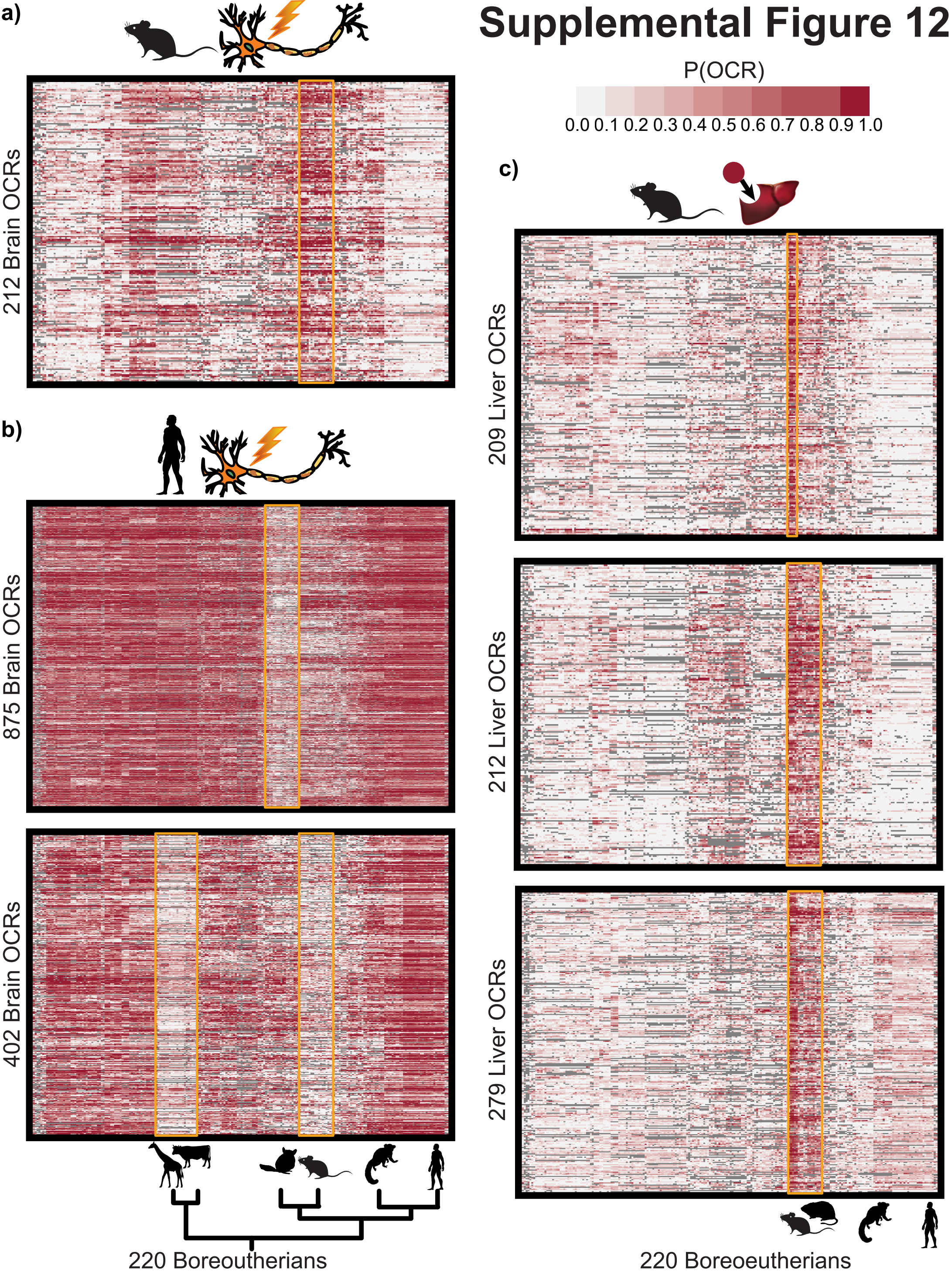

Open Chromatin from Tissue

Supplemental Figure 13

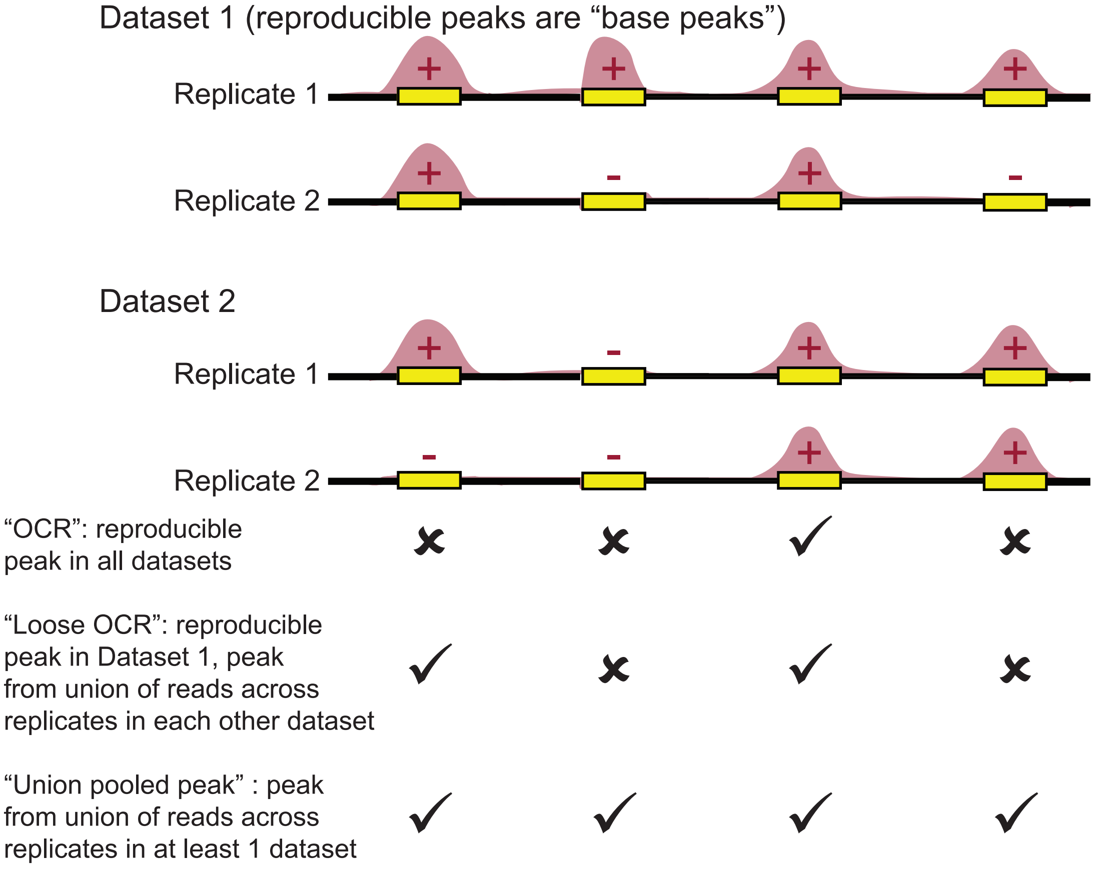

Supplement: Supplementary file 1 — Additional file 1. [file 12864_2022_8450_MOESM1_ESM.pdf]
